# Supplementary material for: Repetitive neuronal activation regulates cellular maturation state via nuclear reprogramming
Source: Nat Commun. 2026 Jul 17;17:5881. doi: 10.1038/s41467-026-74202-w (PMC13379389; doi:10.1038/s41467-026-74202-w)
Supplement: Supplementary file 1 — Supplementary Information [file 41467_2026_74202_MOESM1_ESM.pdf]

Repetitive Neuronal Activation Regulates Cellular Maturation State via Nuclear Reprogramming

Tomoyuki Murano[1], Hideo Hagihara[1], Katsunori Tajinda[2], Keizo Takao[3,4], Yoshihiro Takamiya[1], Kaoru Katoh[5,6,7,8,9], Alfred J. Robison[10], Mitsuyuki Matsumoto[2,11], Masakazu Namihira[5,8,12], Tsuyoshi Miyakawa[1]*

[1]Division of Systems Medical Science, Center for Medical Science, Fujita Health University, Toyoake, Japan
[2]Astellas Research Institute of America, San Diego, CA, USA
[3]Department of Behavioral Physiology, Faculty of Medicine, University of Toyama, Toyama, Japan
[4]Research Center for Idling Brain Science, University of Toyama, Toyama, Japan
[5]Artificial Intelligence Research Center, National Institute of Advanced Industrial Science and Technology (AIST), Tsukuba, Japan
[6]Ph.D. Program in Humanics, School of Integrative and Global Majors, University of Tsukuba, Tsukuba, Japan
[7]The Exploratory Research Center on Life and Living Systems (ExCELLS), National Institutes of Natural Sciences (NINS), Okazaki, Japan
[8]Molecular Biosystem Research Institute, National Institute of Advanced Industrial Science and Technology (AIST), Tsukuba, Japan
[9]Department of Biochemistry and Cell Biology, National Institute of Infectious Diseases (NIID), Tokyo, Japan
[10]Department of Physiology and Neuroscience Program, Michigan State University, East Lansing, MI, USA
[11]Arialys Therapeutics Inc., La Jolla, CA, USA
[12]Laboratory of Neural Regeneration and Brain Repair, Division of Biological Science, Graduate School of Science and Technology, Nara Institute of Science and Technology (NAIST), Nara, Japan

***Correspondence:**
Tsuyoshi Miyakawa

Division of Systems Medical Science, Center for Medical Science, Fujita Health University, 1-98 Dengakugakubo, Kutsukake-cho, Toyoake, Aichi 470-1192, Japan
Telephone: +81-562-93-9376
Fax: +81-562-92-5382
E-mail: miyakawa@fujita-hu.ac.jp

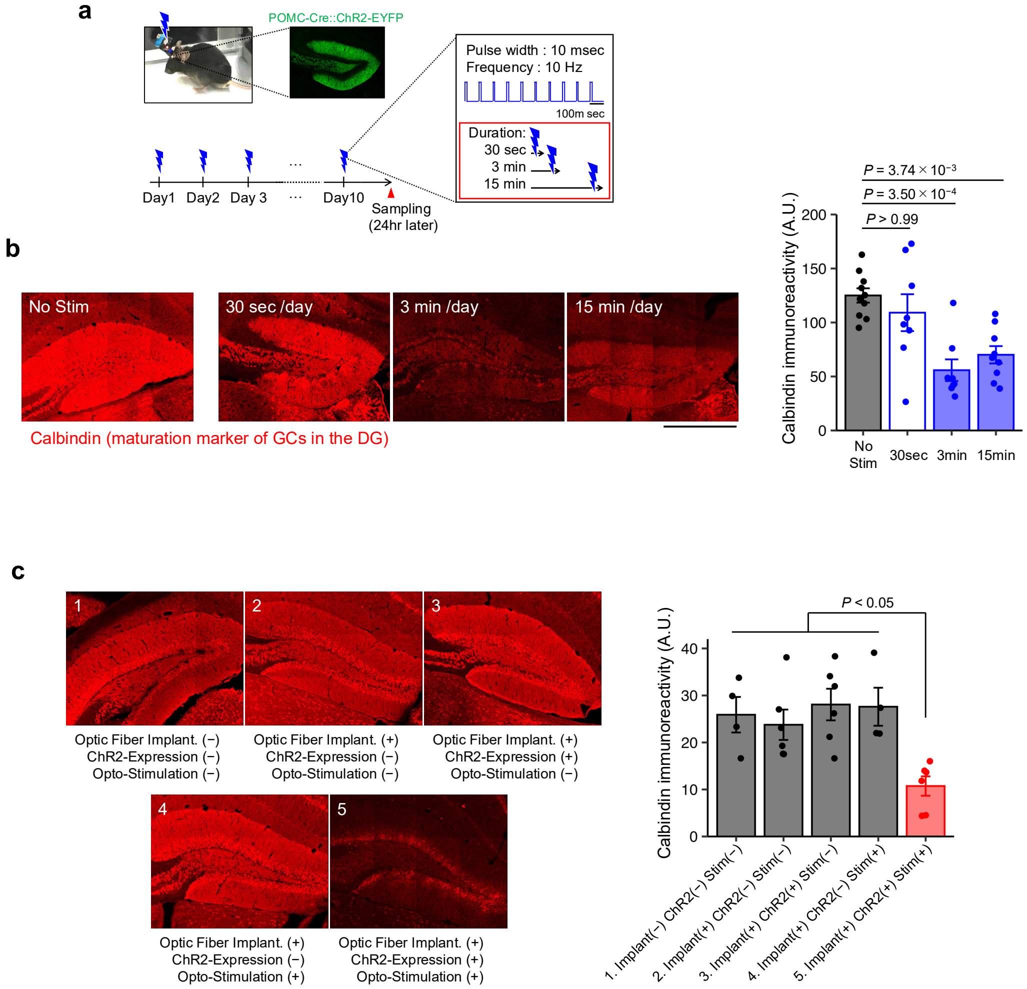

Supplementary Figure 1. Sustained high-frequency neuronal firing induces dematuration in dentate gyrus.

a, REPetitive OPtogenetic Stimulation (REPOPS). ChR2 is exclusively expressed in granule cells (GCs) of the dentate gyrus (DG) under control of the POMC promoter. REPOPS enables frequency- and duration-controlled activation of GCs.

b, Calbindin staining (red) in the DG, 24 hours after 10-day REPOPS at various stimulation durations (30 sec, 3 min, 15 min). Scale bar, 500 µm. Bar graph indicates calbindin immunoreactivity. Mean ± s.e.m. n = 10, 8, 8, and 9 sections from 4 mice/group for No Stim, 30 sec, 3 min, and 15 min, respectively. One-way ANOVA, $F_{(3,31)} = 9.75$, $P = 1.35 \times 10^{-4}$; Bonferroni correction was applied for multiple comparisons. Post hoc P values vs. No Stim: 30 sec, $P > 0.99$; 3 min, $P = 3.50 \times 10^{-4}$; 15 min, $P = 3.74 \times 10^{-3}$. Calbindin expression decreased after 3- or 15-minute duration stimulation but not after 30-sec, suggesting that prolonged neural activation is crucial for dematuration.

c, Calbindin staining in the DG under five experimental conditions (1−5), representing combinations of optic fiber implantation, ChR2 expression in GCs, and optogenetic stimulation. The bar graph shows calbindin immunoreactivity. n = 4, 6, 6, 4, and 6 sections from 4 mice/group for conditions 1–5. One-way ANOVA; $F_{(4, 21)}$ = 5.38, P = 0.0038; Tukey's HSD post hoc test was applied for multiple comparisons. Post hoc *P* values for condition 5 (Implant+/ChR2+/OptStim+) vs. each control: vs. condition 1 (Implant−/ChR2−/OptStim−), *P* = 0.033; vs. condition 2 (Implant +/ChR2−/OptStim−), P = 0.043; vs. condition 3 (Implant +/ChR2+/OptStim−), *P* = 0.0046; vs. condition 4 (Implant +/ChR2−/OptStim+), *P* = 0.015. All other comparisons among conditions 1–4 were not significant (*P* > 0.84). Source data are provided as a Source Data file.

Calbindin expression was significantly reduced only when all three factors—optic fiber implantation, ChR2 expression, and light stimulation—were combined, indicating that the REPOPS-induced reduction in calbindin was not attributable to surgical inflammation, ChR2 expression alone, or light exposure alone.

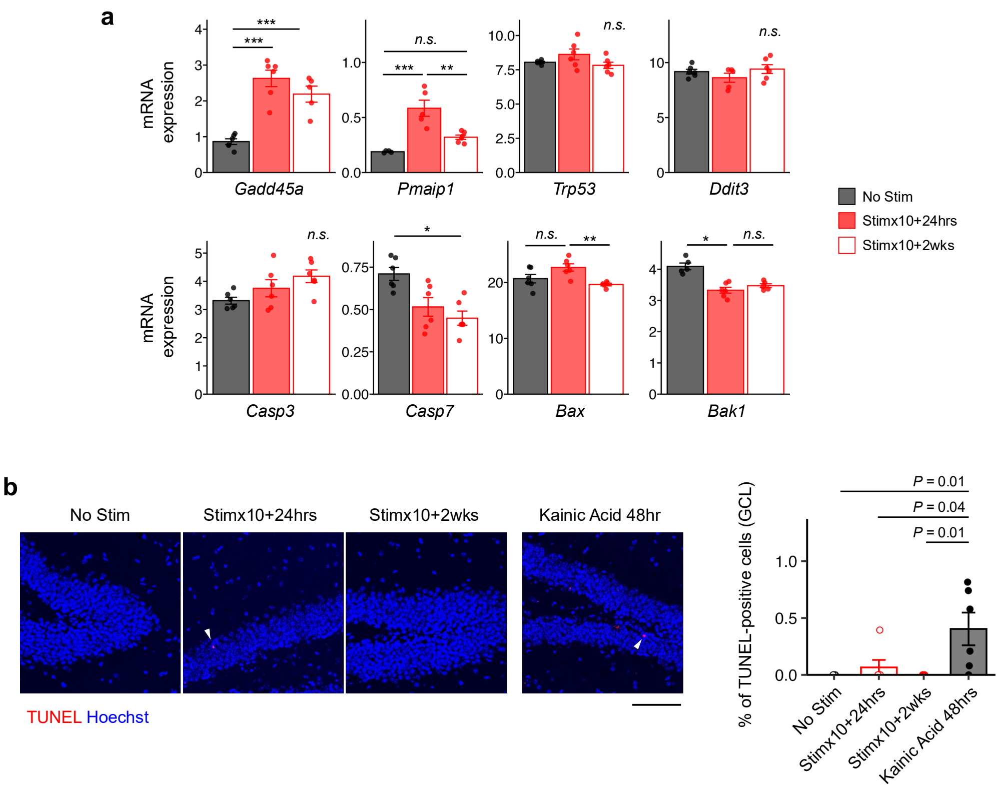

Supplementary Figure 2. REPOPS for 10 days does not induce apoptosis.

a, Expression of apoptosis-related genes (upstream regulators: Gadd45a, Pmaip1, Trp53, and Ddit3; execution markers: Casp3, Casp7, Bax, and Bak1). Data are shown as mean ± s.e.m. (n = 6 mice per group). One-way ANOVA followed by Tukey's HSD post hoc test. $*P < 0.05$, $**P < 0.01$, $***P < 0.001$. Post hoc P values for significant comparisons (vs. No Stim unless specified): *Gadd45a*: Stim×10+24hrs, $P = 2.78\times10^{-4}$; Stim×10+2wks, $P = 6.89\times10^{-4}$. *Pmaip1*: Stim×10+24hrs, $P = 1.01\times10^{-3}$; Stim×10+24hrs vs. Stim×10+2wks, $P = 0.0087$. *Casp7*: Stim×10+24hrs, $P = 0.022$; Stim×10+2wks, $P = 0.0028$. *Bax*: Stim×10+24hrs vs. Stim×10+2wks, $P = 0.0042$. *Bak1*: Stim×10+24hrs, $P = 0.0038$. *Trp53*, *Ddit3, Casp3*: all comparisons not significant.

b, TUNEL staining (red) in the DG of mice treated with 10-day REPOPS or kainic acid (20 mg/kg, 48 hours after intraperitoneal injection). Arrowheads: TUNEL-positive cells. Scale bar, 500 μm. Bar graph shows the percentage of TUNEL-positive cells in the granule cell layer. Mean ± s.e.m. of 6 mice/group. One-way ANOVA; $F_{(3, 20)} = 6.00$, $P = 4.37 \times 10^{-3}$; followed by pairwise *t*-tests with Bonferroni correction. Post hoc P values vs. Kainic Acid: No Stim, $P = 0.01$; Stim×10+24hrs, $P = 0.04$; Stim×10+2wks, $P = 0.01$. All other comparisons not significant. Source data are provided as a Source Data file.

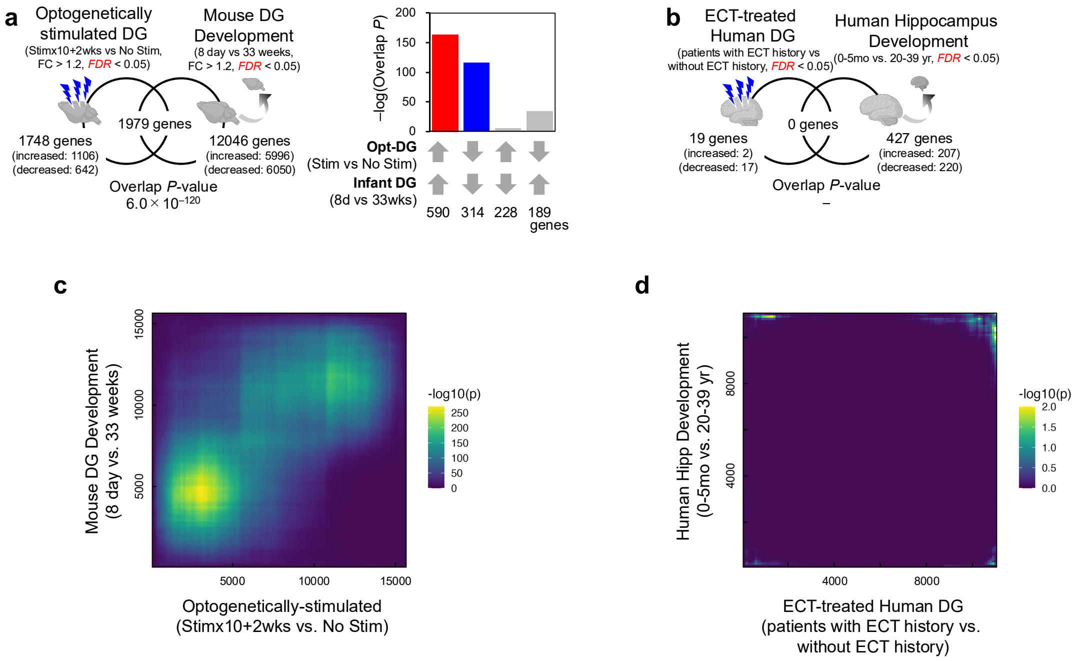

Supplementary Figure 3. Comparison of REPOPS/ECT-induced transcriptional changes with developmental datasets (related to Fig. 1d, f).

a, FDR-based gene overlap analysis. Significant overlap between mouse REPOPS and mouse DG development. Created in BioRender. Murano, T. (2026) https://BioRender.com/p6ed2mm

b, FDR-based gene overlap analysis. No overlap between human DG after ECT treatment and human hippocampal development. Only 19 differentially expressed genes were identified in human DG after ECT treatment (FDR < 0.05), likely due to high inter-individual heterogeneity. Created in BioRender. Murano, T. (2026) https://BioRender.com/p6ed2mm

c, Rank-rank hypergeometric overlap (RRHO) analysis comparing mouse REPOPS and mouse DG development. RRHO heatmaps illustrate genome-wide concordance between datasets. The x- and y-axes indicate rank positions defining up- or down-regulated cutoffs in each dataset, and color intensity represents $-\log_{10}(P)$ values from Fisher's exact tests. Strong concordance was observed for genes at both up-regulated (lower left) and down-regulated (upper right) ends of the ranked lists, compared with the opposite combinations (upper left, lower right).

d, Same as **c**, but comparing human DG after ECT treatment and human hippocampal development.

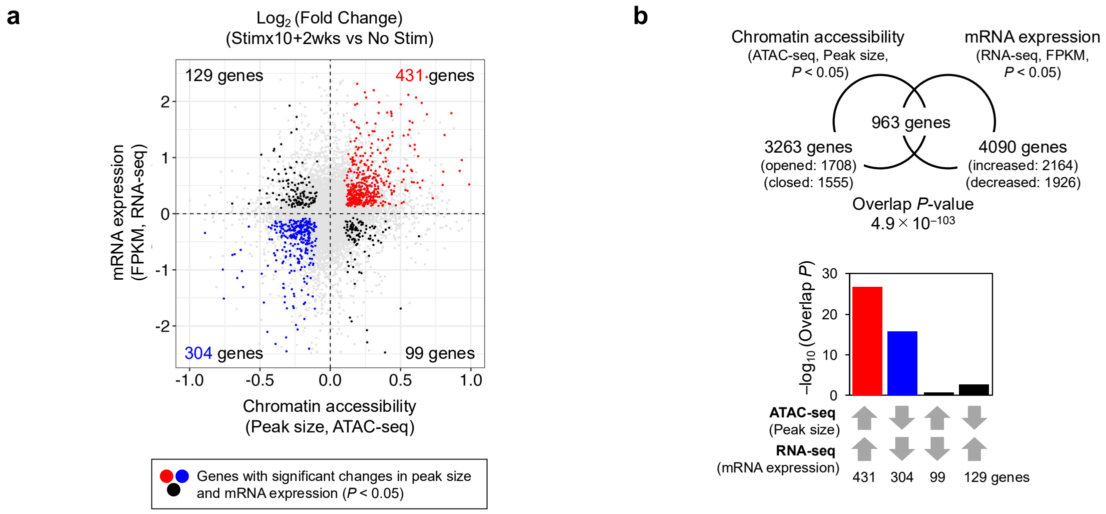

Supplementary Figure 4. Concordance between changes in gene expression and chromatin accessibility.

a, Scatter plot showing the fold change in chromatin accessibility (ATAC-seq peak size, Stim×10+2wks vs. No Stim) versus mRNA expression (RNA-seq, Stim×10+2wks vs. No Stim). Peak size for each gene was calculated as the sum of read counts mapped to the genebody ± 1 kb. Red, blue, and black dots represent genes showing significant increases, decreases, or no change in both chromatin accessibility and mRNA expression ($P < 0.05$).

b, Overlap between genes with significant changes in chromatin accessibility and mRNA expression (Stim×10+2wks vs. No Stim). The bar graph shows the $-\log_{10}$ of overlap *P*-values for up- and down-regulated genes, corresponding to the color scheme in **a**.

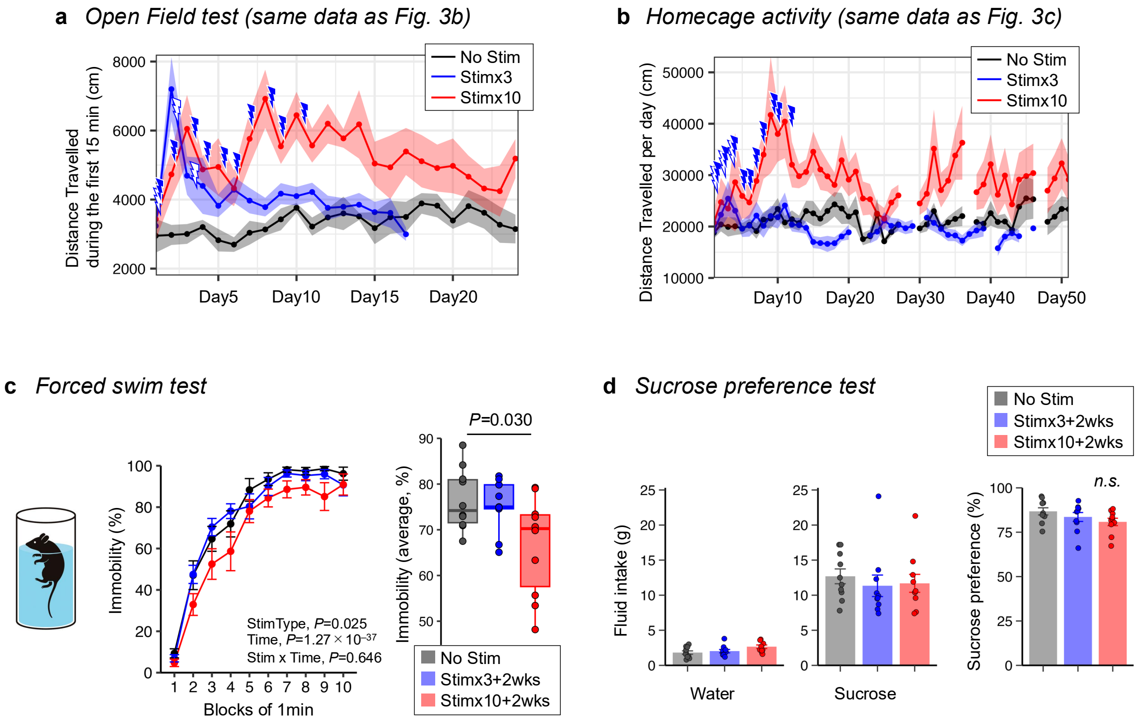

Supplementary Figure 5. REPOPS for 10 days induces anti-depressive behavioral changes in mice.

a, Open field test. Same data as in Fig. 3b, but realigned by the start of the stimulation period.

b, Home cage activity. Same data as in Fig. 3c, but realigned by the start of the stimulation period

c, Immobility (%) in the forced swim test. Left, line plot of immobility per 1-min block; mean ± s.e.m. (n = 10, 9, and 10 mice for No Stim, Stim×3+2wks, and Stim×10+2wks groups, respectively). Two-way repeated-measures ANOVA: stimulation group, $F_{(2, 26)} = 4.28$, $P = 0.025$; Timebin: $F_{(3.61, 93.81)} = 143.73$, $P < 0.001$; interaction: $F_{(7.22, 93.81)} = 0.74$, $P = 0.65$. Observed powers (1−β) were 0.82, >0.99, and 0.30, respectively. Right, box plot summarizing average immobility over 10 min: center line, median; box bounds, 25th and 75th percentiles; whiskers extend to the most extreme data points within 1.5 × IQR from the box; dots, individual mice. One-way ANOVA followed by Tukey's HSD post hoc test. Exact *P* values are provided in the Source Data file.

d, Sucrose preference test. Water and sucrose solution intake (g) and sucrose preference (%). Data are presented as mean ± s.e.m. n = 10 mice/group. One-way ANOVA followed by Tukey's HSD post hoc test. Water intake: $F_{(2, 27)} = 3.14$, $P = 0.060$. Sucrose intake: $F_{(2, 27)} = 0.29$, $P = 0.75$. Sucrose preference: $F_{(2, 27)} = 1.82$, $P = 0.18$. All pairwise comparisons were not significant (all $P > 0.058$). Source data are provided as a Source Data file.

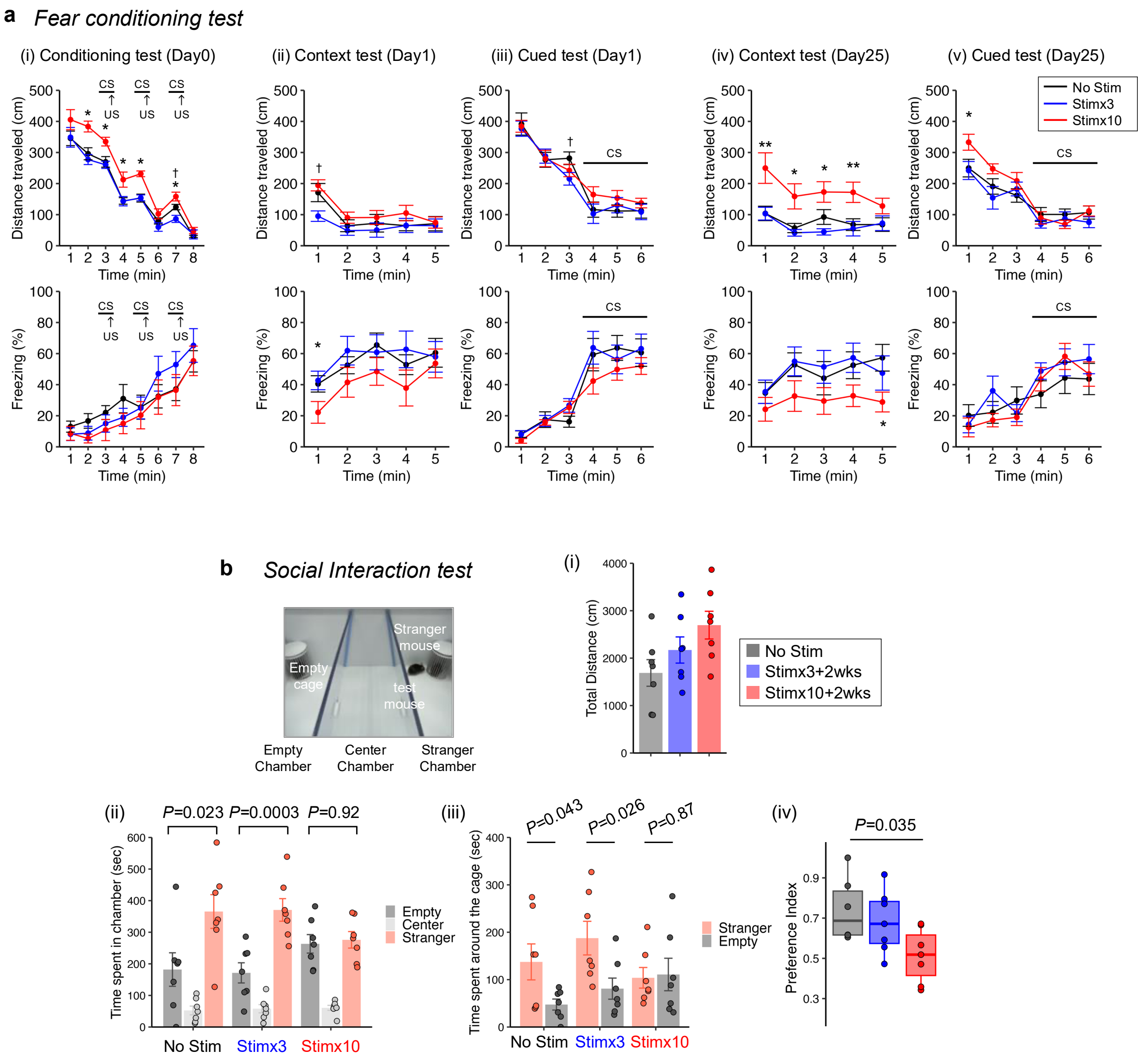

Supplementary Figure 6. Impaired memory and social behavior following repeated optogenetic stimulation.

a, Fear conditioning test. Distance traveled and freezing behavior were assessed during (i) conditioning (Day 0), (ii) context test (Day 1), (iii) cued test (Day 1), (iv) context test (Day 25), and (v) cued tests (Day 25). Data are shown as mean ± s.e.m. for No Stim (n = 10), Stim×3 (n = 8), and Stim×10 (n = 10) groups. Two-way repeated-measures ANOVA followed by Bonferroni correction for multiple comparisons. Asterisks indicate Stim×10 vs. No Stim: *$P < 0.05$, **$P < 0.01$. Daggers indicate Stim×3 vs. No Stim: †$P < 0.05$.

b, Social interaction test. (i) Locomotor activity (n = 7 mice per group). One-way ANOVA: No Stim, $F_{(2, 18)} = 3.172$, $P = 0.0661$. (ii) Time spent in each chamber (empty, center, stranger mouse) (n = 7 mice/group). One-way ANOVA: No Stim, $F_{(2, 18)} = 12.7$, $P = 3.63 \times 10^{-4}$; Stim×3, $F_{(2, 18)} = 30.42$, $P = 1.69 \times 10^{-6}$; Stim×10, $F_{(2, 18)} = 27.33$, $P = 3.51 \times 10^{-6}$. P-values in the graphs

were Bonferroni corrected for multiple comparisons. Observed powers (1−β) were 0.96 (No Stim), 0.99 (Stim×3), and 0.99 (Stim×10). (iii) Time spent around the empty cage vs. the cage with a stranger mouse. Student *t*-test: No Stim, $t_{(12)} = 2.27$, $P = 0.043$; Stim×3, $t_{(12)} = 2.54$, $P = 0.026$; Stim×10, $t_{(12)} = 0.17$, $P = 0.87$. Observed powers (1−β) were 0.78 (No Stim), 0.85 (Stim×3), and 0.06 (Stim×10). (iv) Preference index, calculated as time spent near the stranger mouse divided by total time spent near both cages. One-way ANOVA, $F_{(2, 17)} = 4.18$, $P = 0.0333$, with Bonferroni correction.

Source data are provided as a Source Data file.

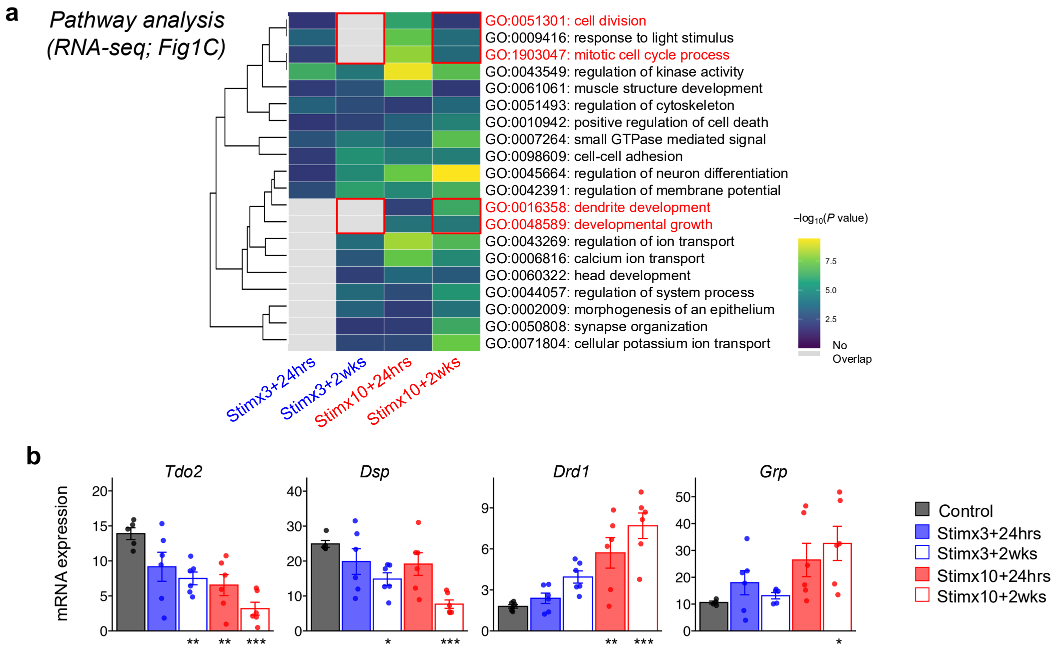

c

Stimx10+24hrs vs. *Stimx3*+24hrs (Fig1c, left)

	Biogroup name	common genes	direction	p-value
1	nuclear envelope	35	down	2.90E-23
2	angiogenesis	20	up	2.90E-18
3	blood vessel morphogenesis	21	up	4.50E-17
4	cell morphogenesis involved in neuron differentiation	24	down	5.60E-16
5	mitochondrion organization	25	up	7.00E-16
6	transcription coregulator activity	28	down	1.20E-15
7	DNA repair	26	down	3.70E-15
8	regulation of intracellular transport	25	down	5.40E-15
9	cell part morphogenesis	25	down	1.90E-14
10	extracellular matrix	14	up	2.70E-14

Stimx10+2wks vs. *Stimx3*+2wks (Fig1c, right)

Rank	Biogroup name	common genes	direction	p-value
1	presynapse	102	up	9.00E-76
2	distal axon	89	up	4.90E-64
3	ribonucleoprotein complex biogenesis	83	down	5.30E-51
4	mitochondrial inner membrane	81	up	6.90E-50
5	cell part morphogenesis	84	up	3.00E-45
6	nuclear outer membrane-endoplasmic reticulum membrane network	84	up	4.00E-45
7	endoplasmic reticulum membrane	82	up	6.60E-45
8	ATP metabolic process	50	up	1.20E-41
9	DNA repair	73	down	1.70E-41
10	secretion	79	up	1.70E-40

Supplementary Figure 7. Expression analysis of transcriptional changes following REPOPS.
a, Gene Ontology (GO) analysis of RNA-seq data from the optogenetically-stimulated DG (Fig. 1c) using Metascape[1]. Top 20 enriched biogroups are shown. Five biogroups were changed in the Stim×10+2wks group but not in Stim×3+2wks group: Two of them were cell-cycle related (*"cell division"* and *"mitotic cell cycle process"*) and the other two were development-related (*"dendrite development"* and *"developmental growth"*).
b, Expression levels of representative markers of mature (Tdo2, Dsp) and immature (Drd1, Grp) DG neurons, which were previously shown to change during development (2.2-, 3.0-, 0.28-, and 0.77-fold; postnatal day 8 vs 33 weeks)[2] are shown. One-way ANOVA followed by Tukey's post hoc test; asterisks indicate significance vs. control (*$P < 0.05$, **$P < 0.01$, ***$P < 0.001$).
c, Pathway analysis comparing Stim×3 and Stim×10 at both 24 hours and 2 weeks after the last stimulation. "Nuclear envelope" was among the top downregulated categories at 24 hours, whereas "presynapse" was prominently altered at 2 weeks. Source data are provided as a

Source Data file.

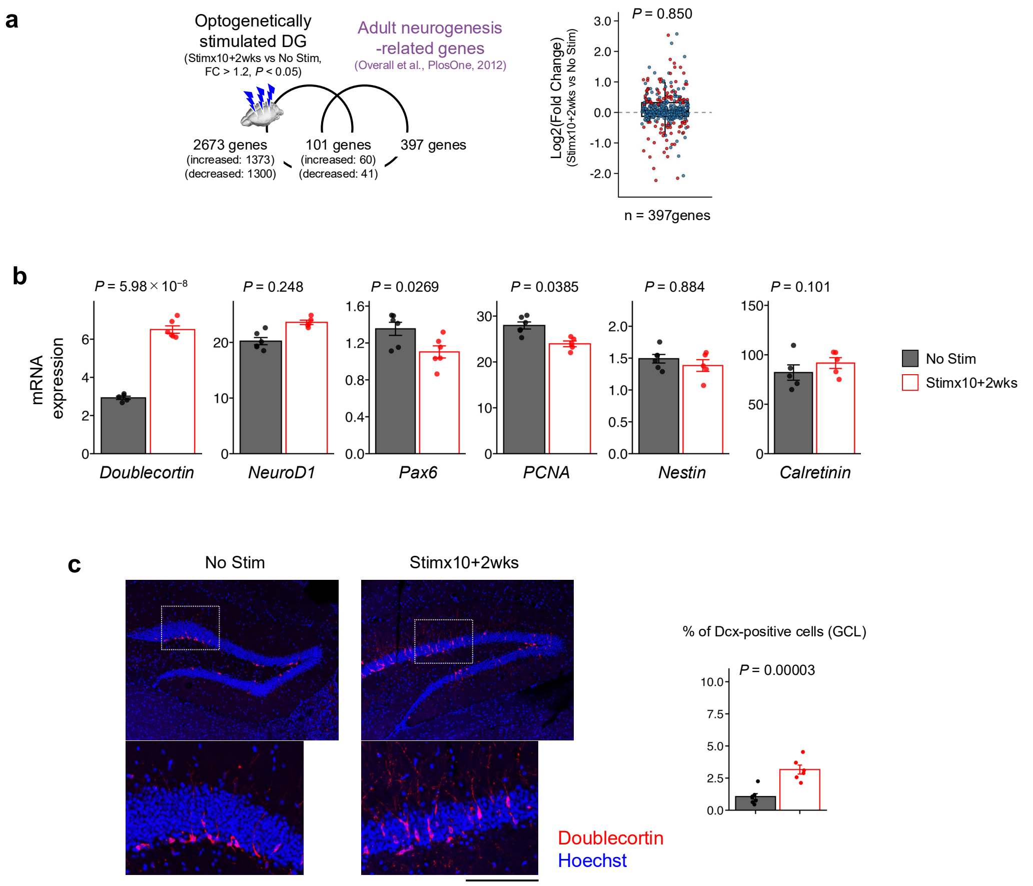

Supplementary Figure 8. REPOPS-induced changes in neurogenesis-related genes.

a, Overlap between genes altered by REPOPS (Stim×10+2wks vs. No Stim) and neurogenesis-related genes[3]. The mean $\log_2$(fold change) did not differ from zero (one-sample t-test, $t_{(391)} = -1.04$, $P = 0.85$). Red and blue dots represent genes with or without significant ($P < 0.05$) expression changes. Created in BioRender. Murano, T. (2026) https://BioRender.com/p6ed2mm

b, Expression of representative neurogenesis-related genes. Data are shown as mean ± s.e.m. (n = 6 mice per group).

c, Immunostaining of Doublecortin in the DG. Scale bar, 100 µm. Representative images from 6 mice per group are shown. Bar graphs show the percentage of Doublecortin-positive cells in the granule cell layer (GCL). Data are shown as mean ± s.e.m. (n = 6 mice per group). Welch's t-test: $t_{(11)} = 5.19$, $P = 3.00 \times 10^{-4}$.

Doublecortin-positive cells were broadly distributed throughout the GCL rather than being confined to the subgranular zone, where adult-born neurons normally appear, implying re-expression of Doublecortin in mature neurons rather than newly generated cells. Doublecortin-positive cells accounted for 1.1% and 3.2% of all GCs in the No Stim and Stim×10+2wks groups, respectively, whereas approximately 90 % of GCs expressed Cyclin B (Supplementary

Fig. 11). These results suggest that the contribution of enhanced adult neurogenesis to REPOPS-induced changes is minimal, and instead, these changes primarily reflect partial reactivation of immature-like transcriptional programs within pre-existing mature neurons. Source data are provided as a Source Data file.

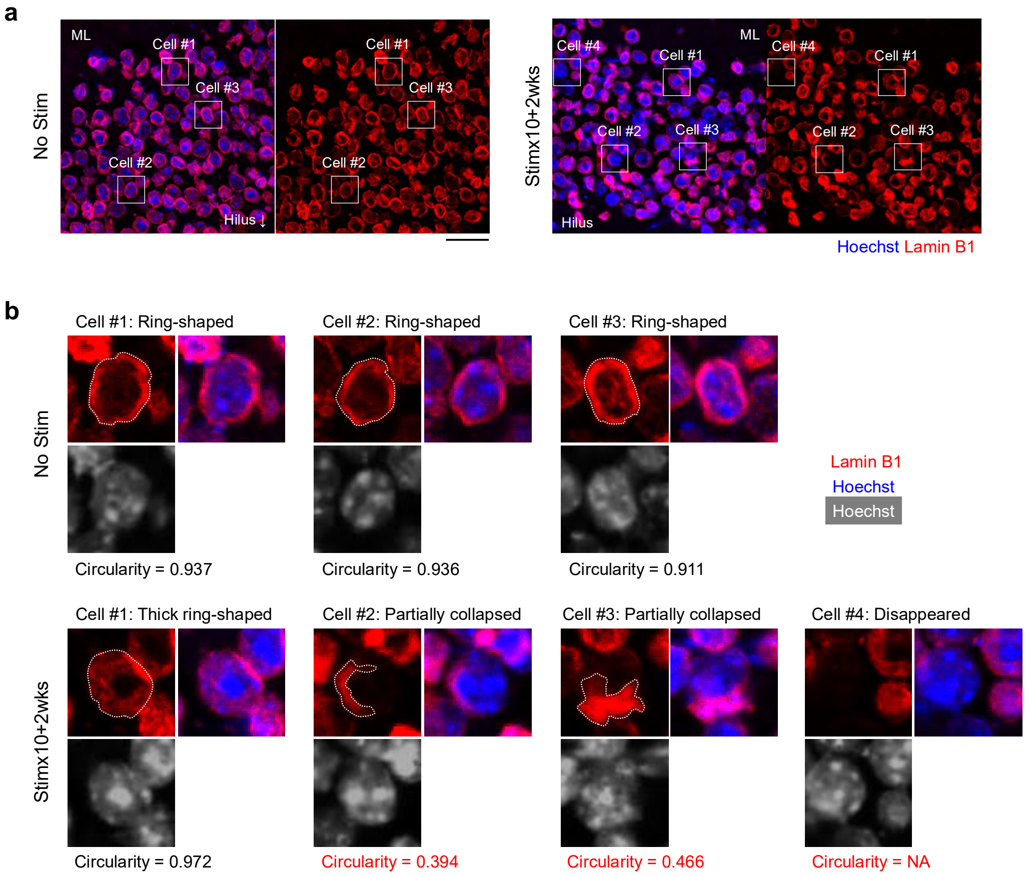

Supplementary Figure 9. Changes in lamin B1 expression and circularity after REPOPS.

a, Lamin B1 (red) and Hoechst (blue) staining in the DG (same data as Fig. 5e). Scale bar, 20 µm.

b, Enlarged images of representative neurons (white boxes in **a**). Red, lamin B1; blue/white, Hoechst.

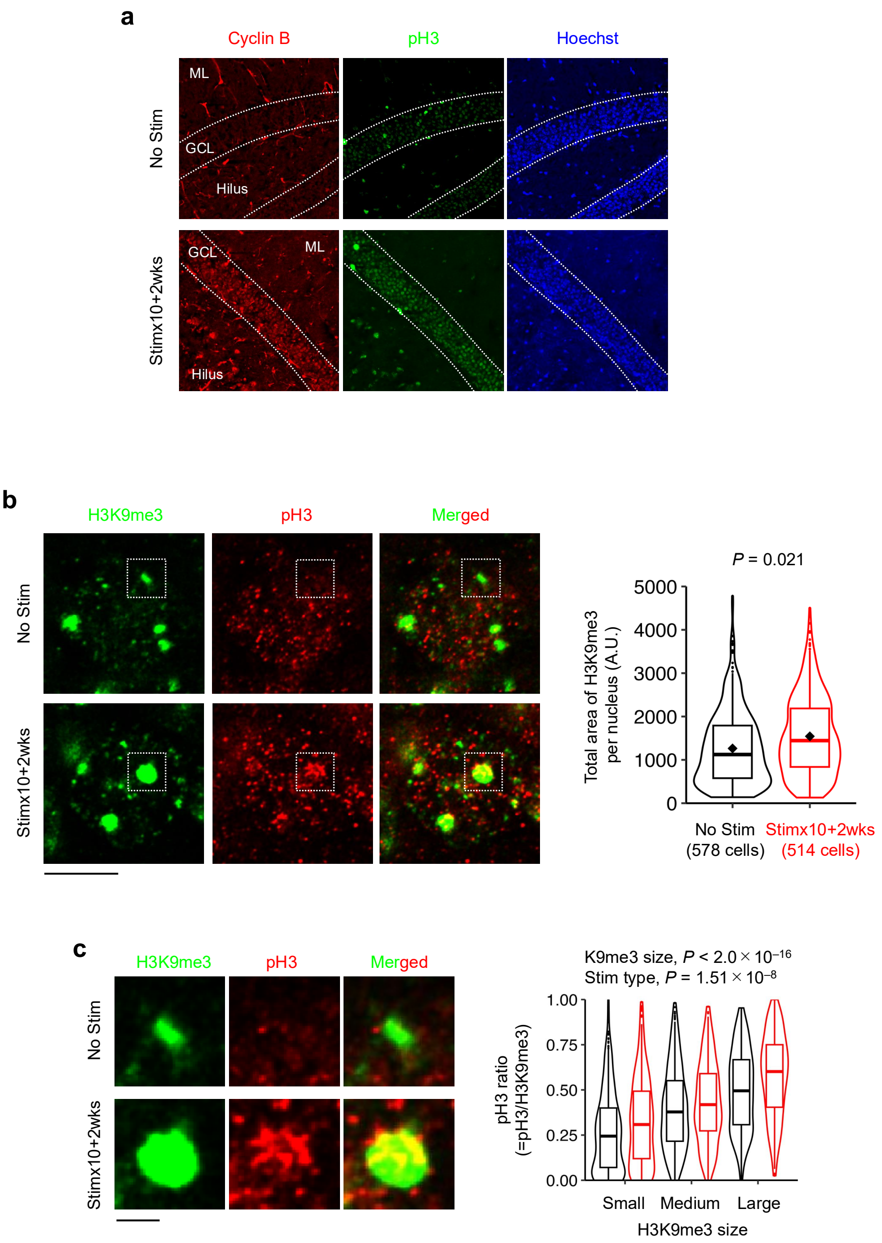

Supplementary Figure 10. Mitosis-like epigenetic modifications are associated with heterochromatin enlargement.

a, Immunostaining of Cyclin B and pH3 in the DG (related to Fig. 5b, f, g). Scale bar, 20 μm. ML, molecular layer; GCL, granule cell layer.

b, STED microscopy images showing phospho-histone H3 (pH3; red) and histone H3 lysine 9 tri-methylation (H3K9me3; green), a marker of highly condensed heterochromatin domains[4]. Scale bar, 5 μm. The violin-box plot shows the size distribution of H3K9me3-positive regions. Center lines represent medians; box boundaries show upper and lower quartiles; whiskers represent maxima and minima; and diamonds denote mean values. 578 and 514 neurons from

four biologically independent samples in the No Stim and Stim×10+2wks groups, respectively. LMM (two-sided): $t_{(5.70)} = 3.17$, $P = 0.021$.

c, Enlarged views of the white boxed regions in **a**. Scale bar, 1 μm. The box plot shows the ratio of pH3 signal coverage per each H3K9me3 dot (i.e., the area of $pH3^{+}H3K9me3^{+}$ double-positive regions normalized by the H3K9me3 dot size). H3K9me3 dots were categorized into Small (< 500 A.U.), Medium (500–1000 A.U.), and Large (>1000 A.U.). A total of 2,177 H3K9me3 dots from 4 mice per group were analysed. Two-way ANOVA (two-sided): H3K9me3 size, $F_{(2, 2171)} = 186.8$, $P < 2.0 \times 10^{-16}$; stimulation type, $F_{(1, 2171)} = 32.3$, $P = 1.51 \times 10^{-8}$, interaction, $F_{(2, 2171)} = 1.51$, $P = 0.22$.

H3K9me3, a marker of condensed heterochromatin, was enlarged by repeated neuronal activation, and its domain size was positively correlated with the pH3 (mitotic marker) signal. These observations suggest that the increase in the mitotic marker and the enlargement of heterochromatin occur in a coordinated manner. Source data are provided as a Source Data file.

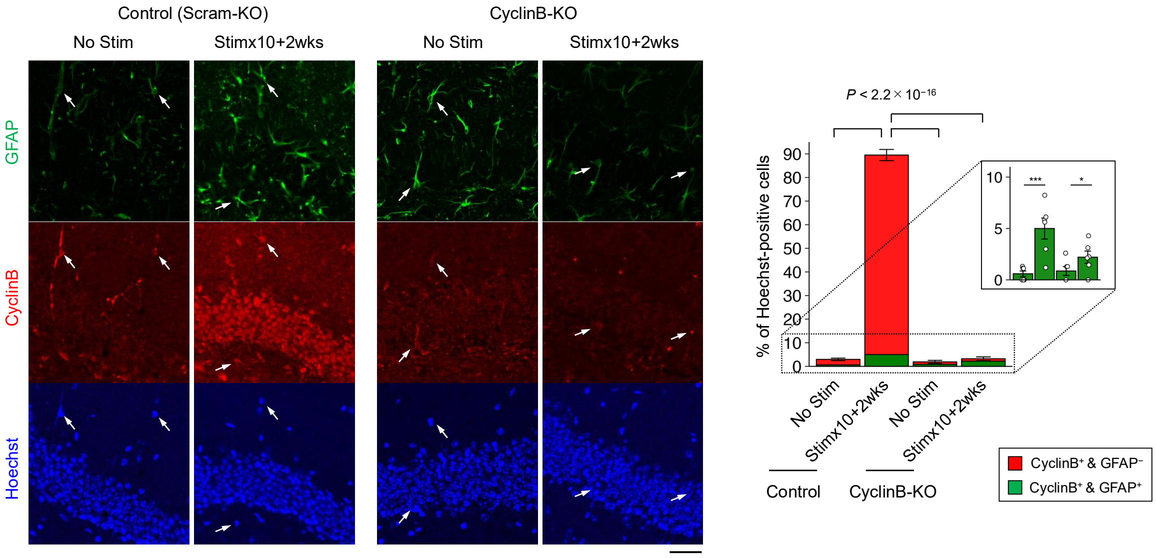

Supplementary Figure 11. Efficient knockout of REPOPS-induced Cyclin B expression.
Confocal images showing co-staining of GFAP and Cyclin B. Representative images from 6 mice per group are shown. Arrows indicate GFAP$^+$/Cyclin B$^+$ astrocytes. Scale bar, 20 µm. Bar graphs show the percentage of Cyclin B$^+$ cells among Hoechst$^+$ cells (left), and GFAP$^+$/Cyclin B$^+$ cells (right). n = 6 mice per group. One-way ANOVA (two-sided); $F_{(3, 20)} = 1092$ and $F_{(3, 20)} = 24.4$, $P < 2.0 \times 10^{-16}$ and $P = 3.45 \times 10^{-4}$, followed by Bonferroni-corrected pairwise comparisons. *$P < 0.05$, **$P < 0.01$, ***$P < 0.001$.

Cyclin B$^+$ cells were markedly increased in the control group following REPOPS but were effectively eliminated in the CyclinB-KO group. The reduction was observed uniformly across the dentate gyrus, confirming efficient and spatially consistent knockout of Cyclin B. Source data are provided as a Source Data file.

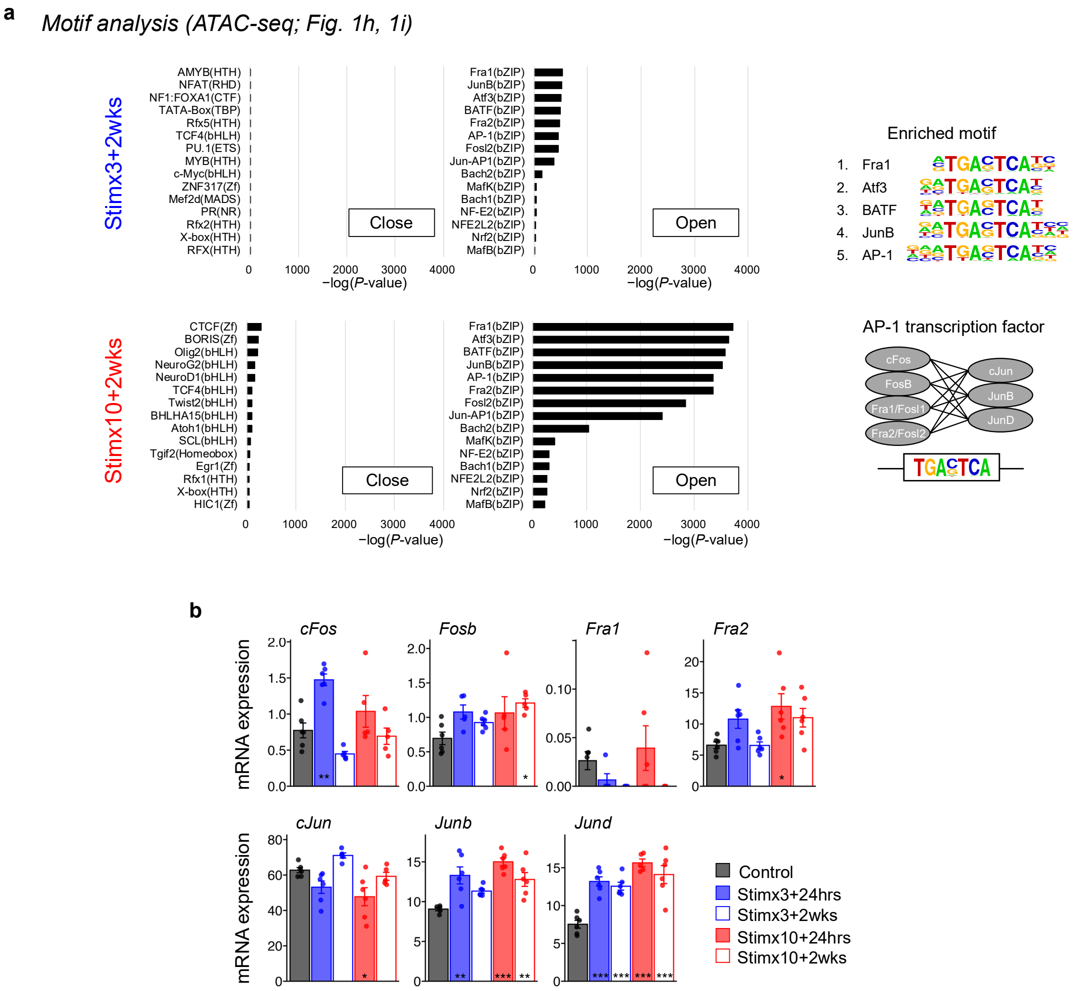

Supplementary Figure 12. AP-1 motifs are enriched in chromatin regions opened by REPOPS.

a, Motif enrichment analysis of ATAC-seq peaks (Fig. 1h, 1i) performed using HOMER. The bar graph shows $-\log_{10}(P\text{-value})$ for similarity between altered chromatin regions in ATAC-seq data and known ChIP-seq profiles. The enriched motifs of the top five in Stim×10+2wks group are shown on the right.

b, Expression of AP-1 genes. Data are shown as mean ± s.e.m. (n = 6 mice per group). One-way ANOVA with Bonferroni correction. Asterisks indicate significance vs. control: $*P < 0.05$, $**P < 0.01$, $***P < 0.001$. Source data are provided as a Source Data file.

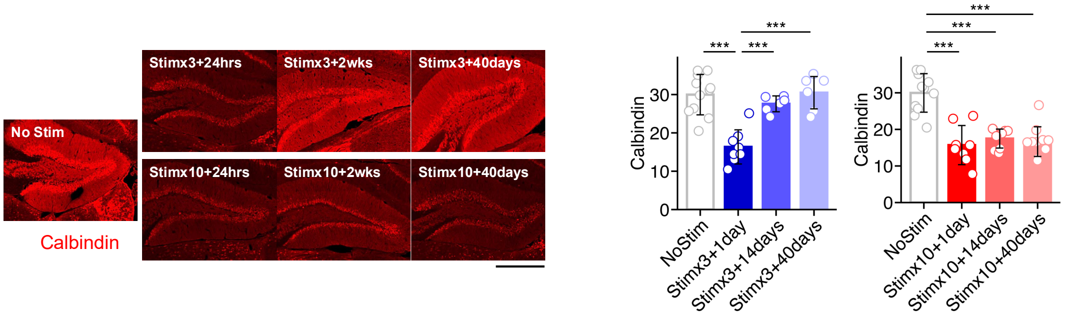

Supplementary Figure 13. Long-term reduction of Calbindin after REPOPS (related to Fig. 7a, b).

Calbindin immunostaining in the dorsal DG. Scale bar, 500 µm. Bar graphs show Calbindin immunoreactivity. n = 11, 8, 6, 7, 8, 9, and 11 sections from 3 mice/group for No Stim, Stim×3+1day, Stim×3+14days, Stim×3+40days, Stim×10+1day, Stim×10+14days, and Stim×10+40days, respectively. Error bars represent mean ± s.e.m. One-way ANOVA: Stim×3, $F_{(3, 28)} = 18.4$, $P = 8.5 \times 10^{-7}$; Stim×10, $F_{(3, 35)} = 23.7$, $P = 1.5 \times 10^{-8}$. Multiple comparisons were performed using Tukey's post hoc test. ***$P < 0.001$. Source data are provided as a Source Data file.

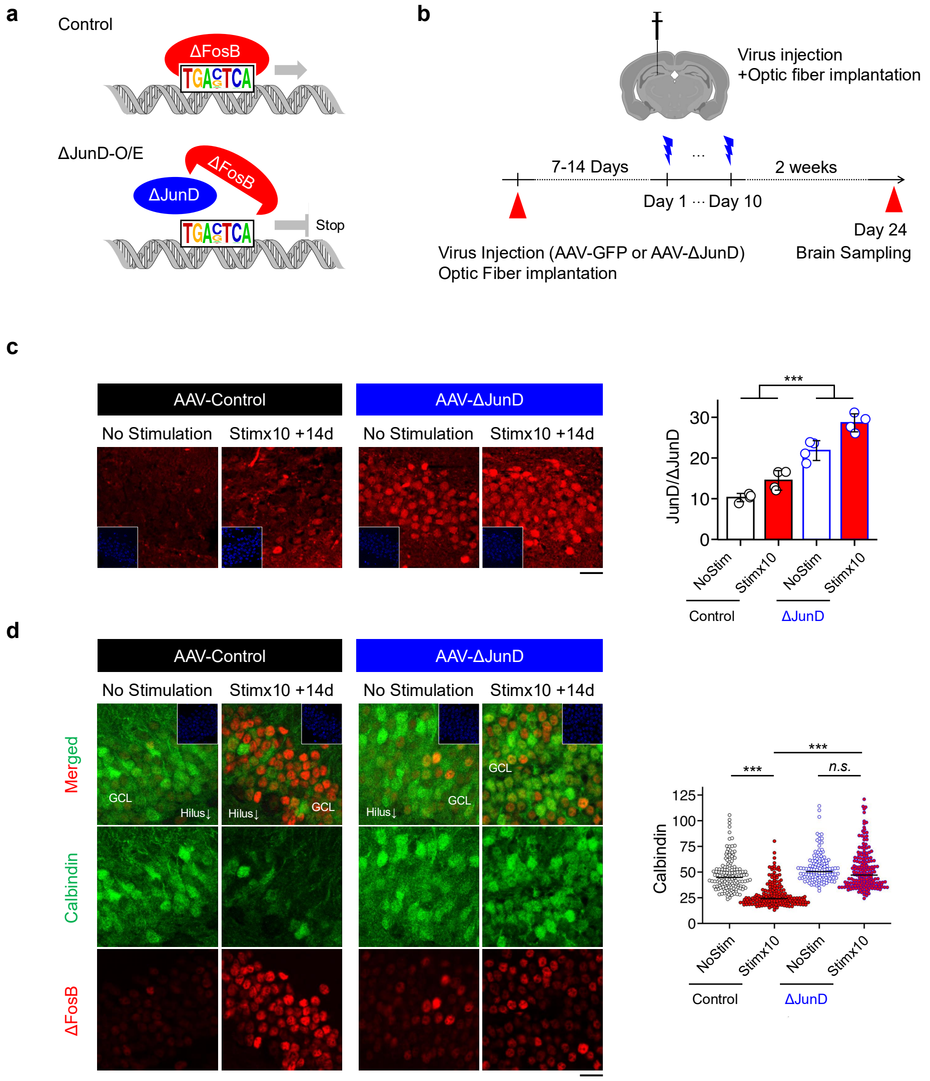

Supplementary Figure 14. ΔFosB reduces calbindin expression in DG neurons.

a, ΔJunD: mutant form of JunD that retains DNA-binding ability but lacks transactivation potential, acting as a dominant-negative inhibitor of AP-1 by forming non-functional dimers with AP-1 family members.

b, Schematic of the experimental design. Created in BioRender. Murano, T. (2026) https://BioRender.com/p6ed2mm

c, Immunostaining of JunD/ΔJunD in the DG; representative images from 4 mice per group are shown. Scale bar, 20 µm. Bar graph shows JunD/ΔJunD immunoreactivity. n = 4 mice per group. Data are mean ± s.e.m. One-way ANOVA: $F_{(3,\ 12)} = 59.56$, $P = 1.77 \times 10^{-7}$; post hoc comparisons using Tukey's test, ***$P < 0.001$.

d, Immunostaining of calbindin and ΔFosB in the DG; representative images from 3 mice per group are shown. Scale bar, 20 µm. Beeswarm plot shows calbindin immunoreactivity in

individual neurons (n = 152, 179, 139, and 179 neurons for AAV-Control/NoStim, AAV-Control/Stimx10, AAV-ΔJunD/NoStim, and AAV-ΔJunD/Stimx10 groups, respectively, from 3 mice per group). Black bars indicate the median. LMM: $F_{(3, 7.81)} = 48.8$, $P = 2.7 \times 10^{-5}$; post hoc pairwise comparisons: $^{*}P < 0.05$, $^{**}P < 0.01$, $^{***}P < 0.001$. Source data are provided as a Source Data file.

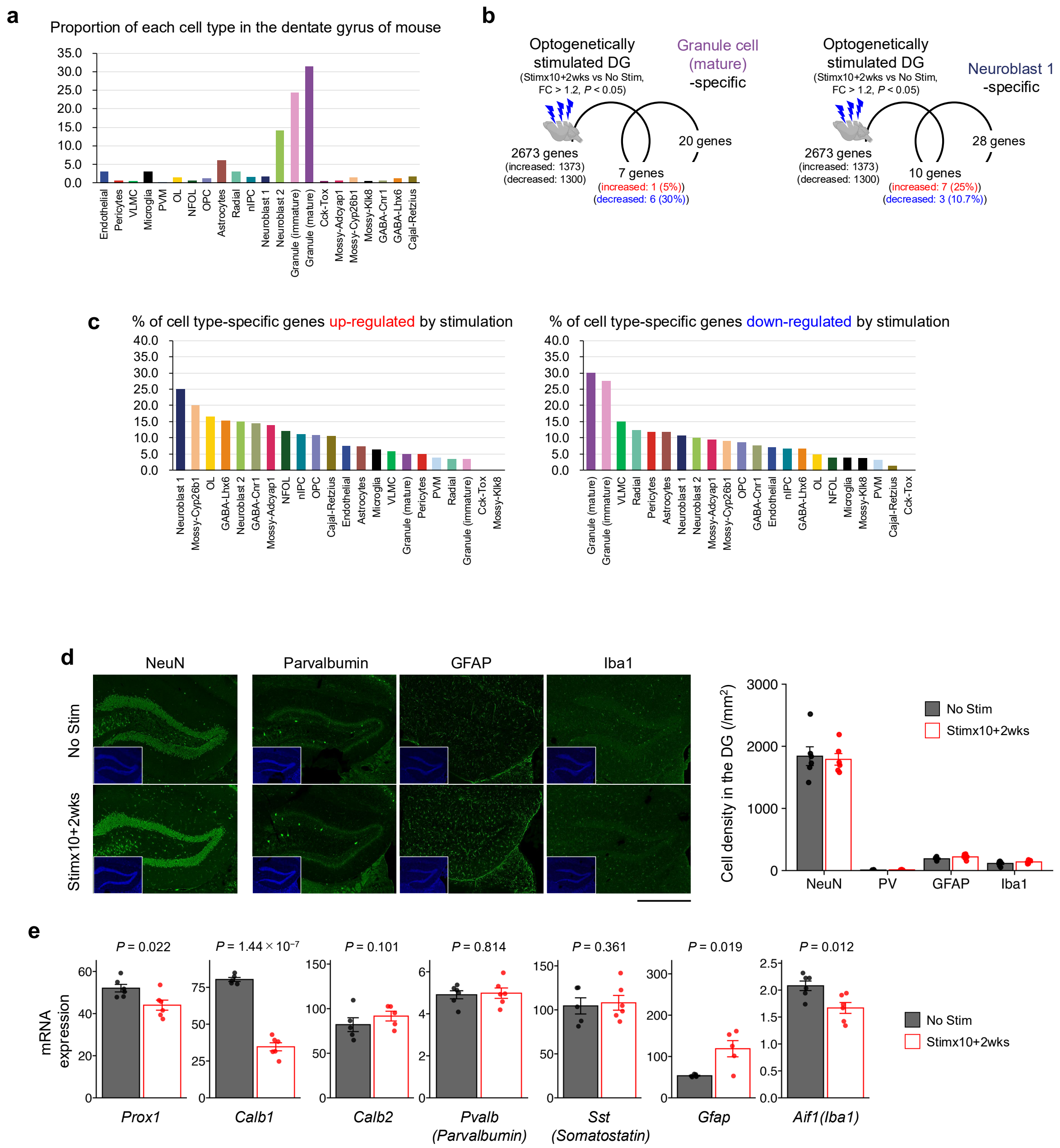

Supplementary Figure 15. REPOPS induces transcriptomic changes across multiple cell types, with predominant effects on granule cells.

a, Proportion of each cell type relative to the total cell population in the mouse dentate gyrus, calculated from the publicly available single-cell RNA-seq dataset of Hochgerner *et al.*, *Nat. Neurosci.*, 2018.

b, Venn diagram showing the overlap between granule cell (mature)– or neuroblast-specific genes and genes significantly altered in the Stim×10+2wks group. Created in BioRender. Murano, T. (2026) https://BioRender.com/p6ed2mm

c, Bar plot illustrating the proportion of genes significantly up- or down-regulated in the

Stim×10+2wks group within each cell type–specific gene set. Cell type classifications on the x-axis are based on Hochgerner et al., 2018.

d, Immunostaining for NeuN (excitatory neurons), PV (interneurons), GFAP (astrocytes), and Iba1 (microglia) in the DG. Scale bar, 500 μm. The bar graph shows the density of marker-positive cells.

e, mRNA expression of representative cell-type marker genes. Data are shown as mean ± s.e.m. (n = 6 mice per group). *P* values were calculated using Welch's *t*-test (two-sided). Source data are provided as a Source Data file.

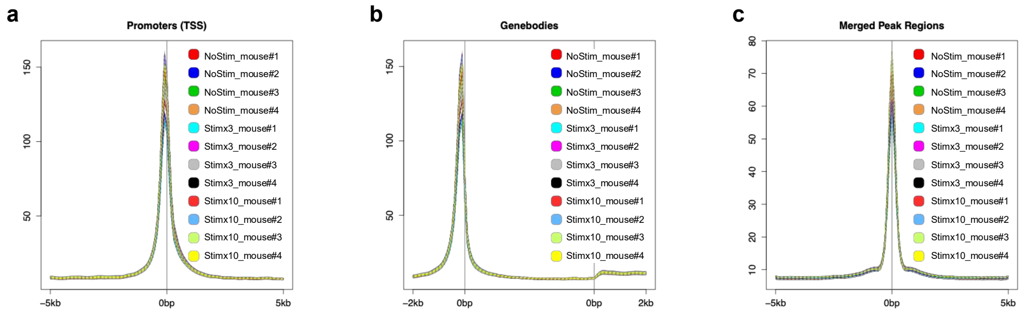

Supplementary Figure 16. ATAC-seq signal enrichment and distribution across genomic features.

a, Aggregate accessibility profiles centered on transcription start sites (TSS ± 5 kb) for all samples.

b, Aggregate accessibility profiles aligned to genebodies (±2 kb), showing the signal distribution along gene bodies.

c, Aggregate accessibility profiles centered on merged peak regions (±5 kb), illustrating chromatin accessibility around consensus peak centers.

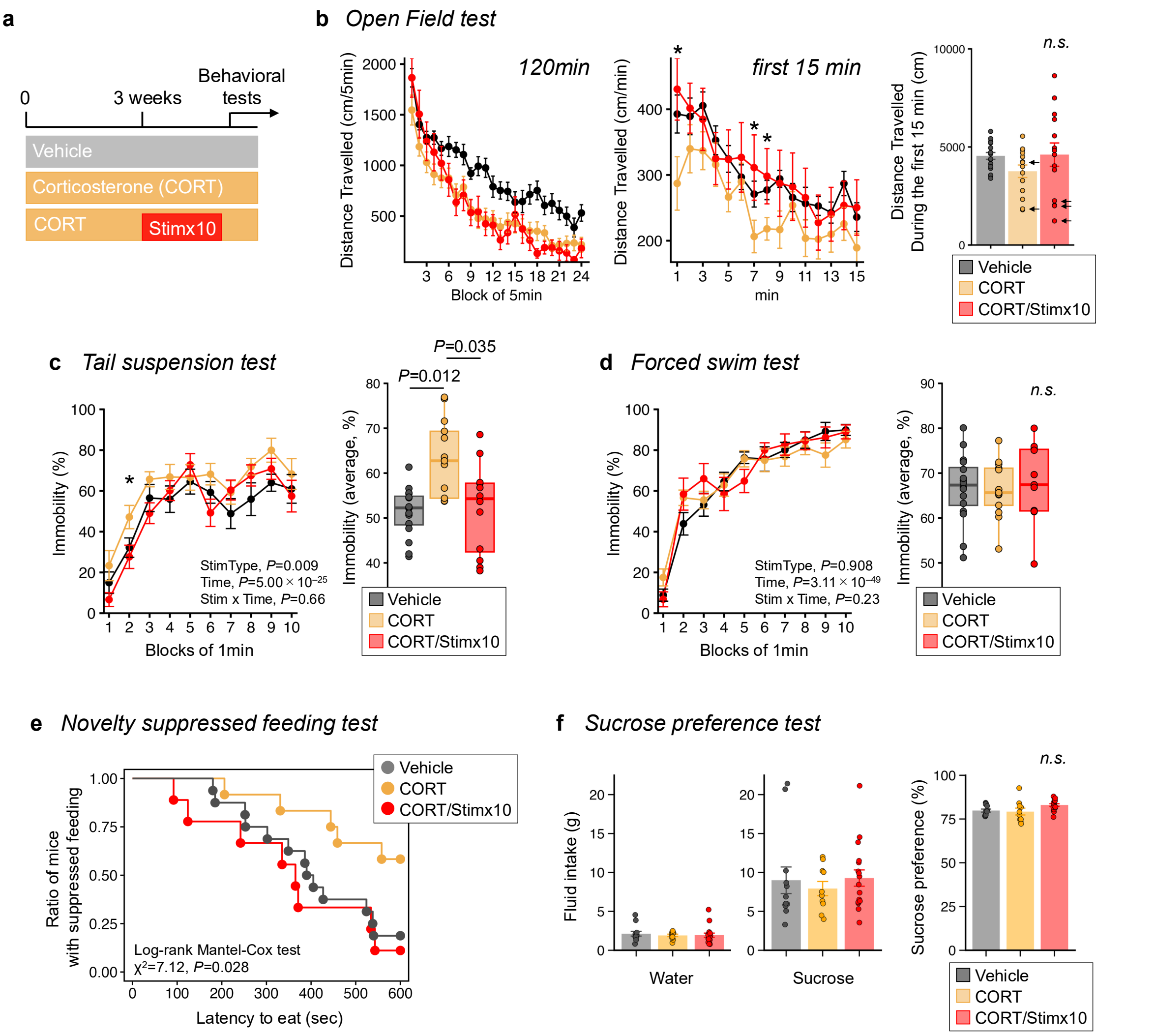

Supplementary Figure 17. REPOPS induces anti-depressive behavioral changes in chronic corticosterone-treated mice.

a, Experimental design: corticosterone (CORT) or vehicle was administered in drinking water for three weeks, followed by REPOPS.

b, Distance traveled in the open field test. Time-course data (120 min) were analyzed by two-way repeated-measures ANOVA with Bonferroni correction; **P* < 0.05. Vehicle: 16 mice; CORT: 14 mice; CORT/Stim×10: 14 mice. Total distance traveled in the first 15 min was compared by one-way ANOVA followed by Tukey's HSD test. Mice that became debilitated during CORT administration and died shortly after the open field test are indicated by arrows (CORT n=2, CORT/Stim×10 n=3); results excluding these mice are reported in Supplementary Results 3 (*P* = 0.0166).

c, Immobility (%) in the tail suspension test. Mean ± s.e.m. Two-way repeated-measures ANOVA results shown in the panel. Box plot summarizes average immobility over 10 min. One-way ANOVA with Bonferroni correction. Vehicle: 16 mice; CORT: 13 mice;

CORT/Stim×10: 12 mice.

d, Same as **c**, for immobility in the forced swim test. Vehicle: 16 mice; CORT: 13 mice; CORT/Stim×10: 11 mice.

e, Novelty-suppressed feeding test. Latency to feed is shown as Kaplan–Meier survival curves depicting the probability of not eating over time. Log-rank Mantel–Cox test: $\chi^2 = 7.12$, $P = 0.028$. Pairwise comparisons revealed significant differences between the Vehicle and CORT groups ($P = 0.025$) and between the CORT and CORT/Stim×10 groups ($P = 0.014$). Vehicle: 16 mice; CORT: 14 mice; CORT/Stim×10: 14 mice.

f, Sucrose preference test. Water and sucrose solution intake (g) and percentage of sucrose preference (%). Mean ± s.e.m. Source data are provided as a Source Data file.

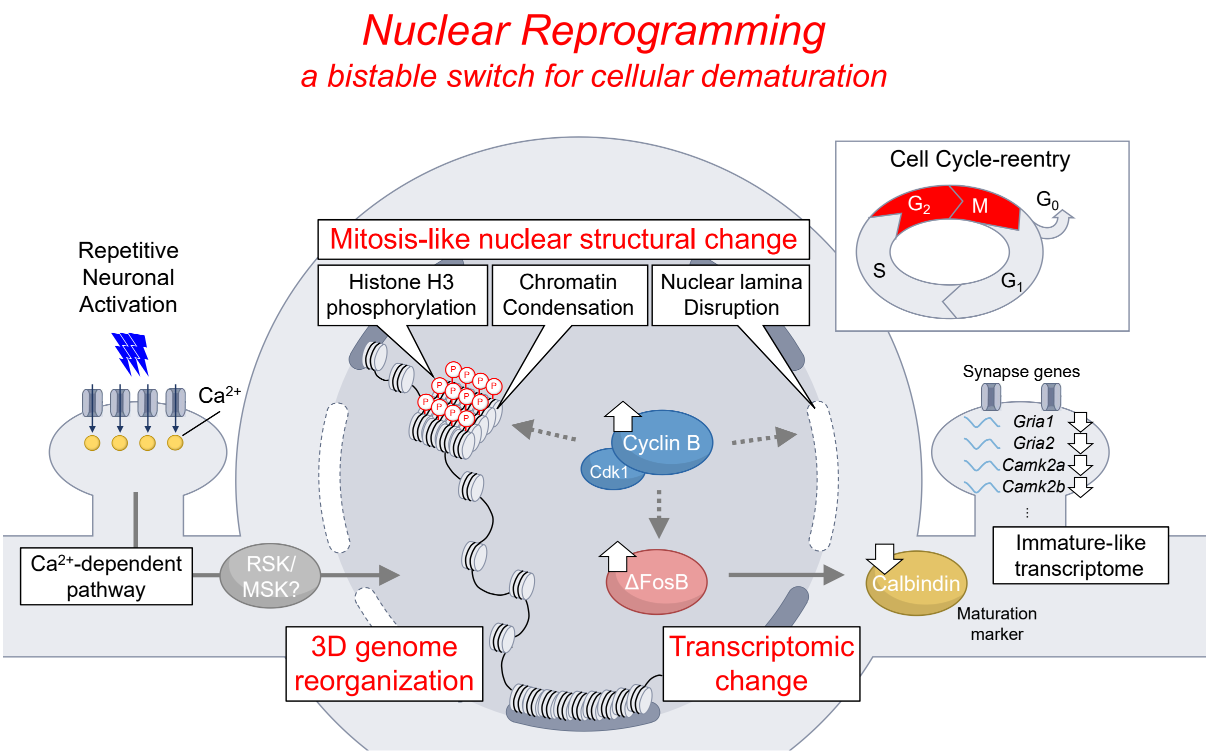

Supplementary Figure 18. Nuclear reprogramming: a bistable switch for cellular dematuration.

Repeated neuronal activation leads to Ca^{2+} influx and subsequent activation of intracellular signaling pathways, inducing a G_2/M-like transcriptional program that includes Ccnb/Cdk1, a key driver of the G_2/M phase transition. This process triggers mitosis-like nuclear structural changes, including nuclear lamina disruption, histone H3 phosphorylation, and chromatin condensation. Repeated activation also induces the expression of AP-1 transcription factors, particularly ΔFosB. Under conditions of sustained Ccnb/Cdk1 activity, ΔFosB expression remains persistently elevated, leading to an immature-like transcriptional signature, including decreased expression of calbindin, a marker of neuronal maturation. This series of processes is referred to as nuclear reprogramming, which may underlie activity-dependent alterations in neural information coding, and it can also serve as a reversible therapeutic intermediate underlying the antidepressant effects of brain stimulation therapies.

Supplementary Data 1. Genes altered by REPOPS that are annotated to the GO term "positive regulation of programmed cell death."

Supplementary Data 2. Genes and related GO terms commonly altered in REPOPS (Stim×10+24hrs and Stim×10+2wks) mice and ECT patients. *P* values for individual genes (Genes sheet) were derived from differential transcript abundance analysis using DESeq2 (Wald test, two-sided). *P* values for GO terms (GO terms sheet) were derived from the Running Fisher test based on Fisher's exact test (one-sided), as implemented in BaseSpace. The Bonferroni correction was used to adjust the significance level according to the number of dataset pairs.

Supplementary Data 3. Pathway analysis of gene sets altered by REPOPS (Stim×10+2wks): genes identified from RNA-seq, genes associated with ATAC-seq peak changes, and their combined gene set. *P* values were derived from the Running Fisher test based on Fisher's exact test (one-sided), as implemented in BaseSpace. The Bonferroni correction was used to adjust the significance level according to the number of dataset pairs.

Supplementary Results

Supplementary Result 1. Behavioral analysis of optogenetically stimulated mice (related to Supplementary Figs. 5 and 6)

We assessed the impact of REPOPS on memory function, which can be impaired in ECT patients by conducting contextual and cued fear-conditioning tests (Supplementary Fig. 6a). In the conditioning session, there was a significant effect of stimulation type on the distance traveled ($P = 5.43 \times 10^{-5}$), but freezing time did not differ significantly ($P = 0.631$; Supplementary Fig. 6a (i)). In the context test 24 hours after the conditioning test, there was no significant effect of stimulation type on the distance traveled or freezing, but mice in the Stim×10 group exhibited a shorter freezing time than No Stim group in the first minute ($P = 0.0394$; Supplementary Fig. 6a (ii)). In the cued test with different contexts, there was no significant effect of stimulation type on the distance traveled and freezing (Supplementary Fig. 6a (iii)). In the context test one month after the conditioning, there was a significant effect of stimulation type on the distance traveled ($P = 0.006$), and mice in the Stim×10 group traveled a significantly longer distance than the No Stim group during minutes 1–4 ($P < 0.05$; Supplementary Fig. 6a (iv)). Mice in the Stim×10 group exhibited a significantly shorter freezing time than the No Stim group in the last minutes ($P = 0.0223$). In the cued test one month after the conditioning test, there was no significant effect of the stimulation type on the distance traveled or freezing time (Supplementary Fig. 6a (v)). Together, these data suggest that REPOPS impairs contextual memory, consistent with known ECT side effects.

We also assessed the impact of REPOPS on sociability using a social interaction test (Supplementary Fig. 6b). Mice in the No Stim and Stim×3 groups spent significantly more time in the chamber with stranger mice than in the chamber with empty cages (Supplementary Fig. 6b (i)). In contrast, no significant difference was observed in the time spent in the stranger and empty chambers in the Stim×10 group (Supplementary Fig. 6b (i)). A similar trend was observed for time spent around the cages (Supplementary Fig. 6b (ii)). The preference index (i.e., the ratio of time spent in the stranger cage to the total time) was significantly smaller in Stim×10 group than that in No Stim group ($P = 0.034$; Supplementary Fig. 6b (iii)). These observations suggest that REPOPS reduces a preference for social novelty in mice. However, it is also possible that increased locomotor activity influenced the social interaction measures. As previously suggested, an enhanced exploratory drive may outweigh attraction to social novelty, leading to an apparent reduction in social preference indices[6]. Considering that

locomotor activity during the social interaction test was moderately elevated in the REPOPS group (Supplementary Fig. 6b(i)), both interpretations remain plausible and the results should be interpreted with caution.

Supplementary Results 2. REPOPS induces transcriptomic changes across multiple cell types, with predominant effects on granule cells (related to Supplementary Fig. 15).

Our bulk RNA-seq analysis (Fig. 1c–f) captured gene expression changes not only in granule cells (GCs) but also in other cell types, including mossy cells, interneurons, and glial cells. To estimate the relative contribution of each cell type, we performed a cell-type enrichment analysis using the dentate gyrus single-cell RNA-seq dataset reported by Hochgerner et al.[5]. Supplementary Fig. 15a shows the cell-type annotations and their relative proportions within the DG, identical to those in the original reference. Marker genes for each cell type were defined as those expressed >10-fold higher in that specific cell type than the mean across all others. We compared these marker genes with the differentially expressed genes identified in the Stim×10+2wks group and calculated the proportion of marker genes that were significantly up- or down-regulated for each cell type (Supplementary Fig. 15b). Genes associated with immature excitatory neurons (Neuroblast1 cluster) were most prominently upregulated, whereas those associated with mature granule cells were most downregulated (Supplementary Fig. 15c). Cell-type-specific genes for other populations (e.g., glial and mossy cells) also showed moderate alterations, indicating that REPOPS broadly influences multiple cellular populations within the DG.

To further validate these findings, we performed immunostaining for NeuN, parvalbumin (PV), GFAP, and Iba1 and quantified the density of marker-positive cells (Supplementary Fig. 15d). None of these markers showed significant differences in the number of positive cells after REPOPS, indicating that the bulk-level transcriptomic changes are unlikely to be explained simply by large shifts in cell numbers. We also analyzed the mRNA expression of representative cell-type marker genes (Prox1 and Calb1 for granule cells, Calb2 for mossy cells, Pvalb and Sst for interneurons, Gfap for astrocytes, and Aif1 (Iba1) for microglia). REPOPS for 10 days significantly reduced the expression of Prox1, Calb1, and Iba1, increased Gfap expression, and did not change the expression of Calb2, Pvalb, or Sst (Supplementary Fig. 15e). Given that granule cells are overwhelmingly predominant within the DG cell composition (Supplementary Fig. 15a, d), these results support the conclusion that, although REPOPS affects multiple cell types, the majority of transcriptomic alterations

observed in our bulk RNA-seq data are primarily driven by granule cells.

Supplementary Results 3. REPOPS reduces depression-like behavior in chronic corticosterone-treated mice (related to Supplementary Fig. 17).

To examine whether the antidepressant-like behavioral effects of REPOPS could be reproduced under depression-like conditions, we used a chronic corticosterone (CORT) administration model**,** a validated paradigm that reliably induces depression-like phenotypes via sustained activation of the hypothalamic–pituitary–adrenal (HPA) axis[7]. Mice received chronic CORT treatment for three weeks, followed by ten consecutive days of REPOPS (CORT/Stim×10 group) or no stimulation (CORT group) (Supplementary Fig. 17a).

In the open field test, mice in the CORT and CORT/Stim×10 groups exhibited markedly reduced locomotor activity compared with controls (Supplementary Fig. 17b). Immediately after exposure to the open field, mice in the CORT/Stim×10 group showed higher locomotor activity than in the CORT group at 1, 7, and 8 min of the test (Supplementary Fig. 17b). There was no significant difference in total distance traveled in the first 15 min among the three groups. However, after excluding mice that showed severe CORT-related debilitation and died shortly after the open field test (arrows in Supplementary Fig. 17b; CORT n=2, CORT/Stim×10 n=3), locomotor activity in the CORT/Stim×10 group was significantly greater than in the CORT group (P = 0.0166; one-way ANOVA followed by Tukey's HSD test). These results suggest that REPOPS increased locomotor activity under CORT-induced conditions (Supplementary Fig. 17b). In the tail suspension test, CORT-treated mice showed significantly increased immobility relative to vehicle controls (P = 0.012), whereas immobility was significantly decreased in the CORT/Stim×10 group compared to CORT-treated mice (P = 0.035) (Supplementary Fig. 17c). When sex was included as a covariate, ANCOVA still confirmed a significant main effect of the stimulation group on locomotor activity in the open field test ($F_{(2,\ 34)}$ = 6.39, P = 0.0044) and on immobility in the tail suspension test ($F_{(2,\ 35)}$ = 5.97, P = 0.0059). There were no significant changes in immobility in the forced swim test (Supplementary Fig. 17d).

In the novelty-suppressed feeding test, feeding latency differed significantly among the three groups (log-rank Mantel–Cox test: χ^2 = 7.12, P = 0.028). Post hoc pairwise comparisons revealed that feeding latency was significantly longer in the CORT group than in controls (χ^2 = 5.00, P = 0.025), whereas the CORT/Stim×10 group exhibited a significantly shorter latency than in the CORT group (χ^2 = 6.01, P = 0.014) (Supplementary

Fig. 17e). When sex was included as a covariate, ANCOVA still confirmed a significant main effect of the stimulation group on feeding latency ($F_{(2, 35)} = 3.41$, $P = 0.046$). Together, these data demonstrate that REPOPS partially reverses CORT-induced depression- and anxiety-like behavioral deficits and supports the notion that REPOPS recapitulates key therapeutic effects of ECT under pathological conditions.

Supplementary References

1. Zhou, Y. *et al.* Metascape provides a biologist-oriented resource for the analysis of systems-level datasets. *Nat Commun* **10**, 1523 (2019).

2. Murano, T., Hagihara, H., Tajinda, K., Matsumoto, M. & Miyakawa, T. Transcriptomic immaturity inducible by neural hyperexcitation is shared by multiple neuropsychiatric disorders. *Commun Biol* **2**, 1–11 (2019).

3. Overall, R. W., Paszkowski-Rogacz, M. & Kempermann, G. The Mammalian Adult Neurogenesis Gene Ontology (MANGO) Provides a Structural Framework for Published Information on Genes Regulating Adult Hippocampal Neurogenesis. *PLOS ONE* **7**, e48527 (2012).

4. Padeken, J., Methot, S. P. & Gasser, S. M. Establishment of H3K9-methylated heterochromatin and its functions in tissue differentiation and maintenance. *Nat Rev Mol Cell Biol* **23**, 623–640 (2022).

5. Hochgerner, H., Zeisel, A., Lönnerberg, P. & Linnarsson, S. Conserved properties of dentate gyrus neurogenesis across postnatal development revealed by single-cell RNA sequencing. *Nature Neuroscience* **21**, 290 (2018).

6. Kim, D. G. *et al.* Social Interaction Test in Home Cage as a Novel and Ethological Measure of Social Behavior in Mice. *Experimental Neurobiology* **28**, 247–260 (2019).

7. Shoji, H., Maeda, Y. & Miyakawa, T. Chronic corticosterone exposure causes anxiety- and depression-related behaviors with altered gut microbial and brain metabolomic profiles in adult male C57BL/6J mice. *Molecular Brain* **17**, 79 (2024).
