# Supplementary material for: Repetitive neuronal activation regulates cellular maturation state via nuclear reprogramming
Source: Nat Commun. 2026 Jul 17;17:5881. doi: 10.1038/s41467-026-74202-w (PMC13379389; doi:10.1038/s41467-026-74202-w)
Supplement: Supplementary file 5 — Reporting Summary [file 41467_2026_74202_MOESM5_ESM.pdf]

nature portfolio

Corresponding author(s): Tsuyoshi Miyakawa

Last updated by author(s): 2026 05 03

Reporting Summary

Nature Portfolio wishes to improve the reproducibility of the work that we publish. This form provides structure for consistency and transparency in reporting. For further information on Nature Portfolio policies, see our Editorial Policies and the Editorial Policy Checklist.

Statistics

For all statistical analyses, confirm that the following items are present in the figure legend, table legend, main text, or Methods section.

n/a	Confirmed	
☐	☒	The exact sample size (*n*) for each experimental group/condition, given as a discrete number and unit of measurement
☐	☒	A statement on whether measurements were taken from distinct samples or whether the same sample was measured repeatedly
☐	☒	The statistical test(s) used AND whether they are one- or two-sided<br>*Only common tests should be described solely by name; describe more complex techniques in the Methods section.*
☐	☒	A description of all covariates tested
☐	☒	A description of any assumptions or corrections, such as tests of normality and adjustment for multiple comparisons
☐	☒	A full description of the statistical parameters including central tendency (e.g. means) or other basic estimates (e.g. regression coefficient) AND variation (e.g. standard deviation) or associated estimates of uncertainty (e.g. confidence intervals)
☐	☒	For null hypothesis testing, the test statistic (e.g. *F*, *t*, *r*) with confidence intervals, effect sizes, degrees of freedom and *P* value noted<br>*Give P values as exact values whenever suitable.*
☒	☐	For Bayesian analysis, information on the choice of priors and Markov chain Monte Carlo settings
☐	☒	For hierarchical and complex designs, identification of the appropriate level for tests and full reporting of outcomes
☐	☒	Estimates of effect sizes (e.g. Cohen's *d*, Pearson's *r*), indicating how they were calculated

Our web collection on statistics for biologists contains articles on many of the points above.

Software and code

Policy information about availability of computer code

Data collection	Confocal and STED images were acquired with a Zeiss LSM 700 (ZEN, Zeiss) and a Leica TCS SP8 STED 3X system (Leica Microsystems) using the manufacturers' acquisition software. Behavioral tests (open field, home-cage monitoring, tail suspension, forced swim, fear conditioning, social interaction and NSFT) were video-recorded in O'Hara behavioral apparatus with overhead CCD cameras. In vivo Ca^{2+} imaging was performed with a miniature microscope (nVoke, Inscopix) after GRIN-lens implantation. No custom/in-house software was used for data acquisition.
Data analysis	Confocal ROIs were delineated and quantified in ZEN and ImageJ/Fiji v1.54p. STED images (20-nm pixel size) were deconvolved in Huygens and further processed with a custom MATLAB script (R2022a, Image Processing Toolbox). Behavioral videos were analyzed automatically with ImageJ-based plugins (Image OF, ImageCSI). Ca^{2+} movies were processed in Inscopix Data Processing Software (IDPS 1.8.0) (motion correction, ΔF/F, PCA/ICA, event detection). RNA-seq reads were mapped to mm10 with TopHat v2.0.14, summarized with Genedata Profiler Genome v10.1.12, and differential expression was performed in R using DESeq2 v1.50.2. ATAC-seq reads were aligned with BWA, peaks were called with MACS2 v2.1.0 ($P = 1\times10^{-7}$, --nomodel), counts were obtained with Subread, and differential accessibility was tested with DESeq2 v1.50.2 (adjusted $P < 0.05$); motif enrichment used HOMER. Public transcriptomic datasets for meta-analysis were obtained via the Illumina BaseSpace Correlation Engine. The kinome dendrogram in Fig. 2f was generated with KinMap (Eid et al., 2017; http://www.kinhub.org/kinmap/), and schematic illustrations in Figs. 1f, 5c, 6a and Extended Data Figs. 3a, 3b, 8a, 14b, 15b were created with BioRender (https://www.biorender.com). All analysis scripts and processed data supporting this study are available on the authors' GitHub (https://github.com/tmurano/REPeatedOPtogeneticStimulation) and archived at Zenodo (https://doi.org/10.5281/zenodo.19904295).

For manuscripts utilizing custom algorithms or software that are central to the research but not yet described in published literature, software must be made available to editors and reviewers. We strongly encourage code deposition in a community repository (e.g. GitHub). See the Nature Portfolio guidelines for submitting code & software for further information.

Data

Policy information about availability of data

All manuscripts must include a data availability statement. This statement should provide the following information, where applicable:

- Accession codes, unique identifiers, or web links for publicly available datasets
- A description of any restrictions on data availability
- For clinical datasets or third party data, please ensure that the statement adheres to our policy

RNA-seq data generated in this study from mouse dentate gyrus are available in GEO under accession GSE227200. ATAC-seq data are available in GEO under accession GSE227201. Post-mortem human dentate gyrus RNA-seq data were obtained from the SRA (SRP241159). Public transcriptomic datasets for meta-analysis were retrieved from the Illumina BaseSpace Correlation Engine. De-identified ECT-related clinical information was provided by Astellas Pharma Inc. under their internal data-protection procedures and cannot be publicly redistributed. Raw imaging data are available from the RIKEN SSBD repository (ID 335), and confocal images are available on figshare (DOI https://doi.org/10.6084/m9.figshare.28853303

Research involving human participants, their data, or biological material

Policy information about studies with human participants or human data. See also policy information about sex, gender (identity/presentation), and sexual orientation and race, ethnicity and racism.

Reporting on sex and gender	The de-identified clinical datasets related to electroconvulsive therapy (ECT) were provided by Astellas Pharma Inc. Sex was not used as an analytical variable in this study, because the analytical focus was on disorder- and ECT-history-related transcriptomic comparisons, and the available subgroup sizes were too small for adequately powered sex-stratified analyses.
Reporting on race, ethnicity, or other socially relevant groupings	The de-identified dataset also included race/ethnicity information. These variables were not analyzed or reported in this study because the analytical focus was on ECT-history-based comparisons, and sample sizes were insufficient for race/ ethnicity-stratified analyses.
Population characteristics	Human-related data in this study consisted of two sources: (i) a publicly available post-mortem dentate gyrus RNA-seq dataset from the Sequence Read Archive (SRP241159) and (ii) a de-identified ECT-related clinical dataset provided by Astellas Pharma Inc. None of these datasets contained directly identifying personal information for the authors.
Recruitment	No human participants were directly recruited; analyses used pre-existing de-identified data.
Ethics oversight	Analysis of publicly available, de-identified RNA-seq data (SRA SRP241159) did not require additional ethics approval. Access to the de-identified ECT-related datasets (including sex and race/ethnicity) was granted by Astellas Pharma Inc. under their ethical and data-protection framework. No identifiable human data were handled by the authors, and individual-level records obtained from these third parties cannot be publicly redistributed.

Note that full information on the approval of the study protocol must also be provided in the manuscript.

Field-specific reporting

Please select the one below that is the best fit for your research. If you are not sure, read the appropriate sections before making your selection.

☒ Life sciences ☐ Behavioural & social sciences ☐ Ecological, evolutionary & environmental sciences

For a reference copy of the document with all sections, see nature.com/documents/nr-reporting-summary-flat.pdf

Life sciences study design

All studies must disclose on these points even when the disclosure is negative.

Sample size	Sample sizes were determined by reference to similar previous publications from this and related laboratories using comparable dentate-gyrus paradigms (e.g., refs. 27, 28 in the Methods); for sequencing experiments, the smallest group sizes commonly accepted in the field for mouse RNA-seq/ATAC-seq were used, also constrained by cost. No formal a priori power calculation was performed. Post hoc power analyses confirmed that the achieved statistical power (1−β) exceeded 0.80 for the main effects in most tests, indicating that the sample sizes used were sufficient. Exact sample sizes for each experiment are reported in the figure legends.
Data exclusions	Pre-established exclusion criteria were applied: (1) for in vivo Ca^{2+} imaging, mice were excluded if viral expression was insufficient or mistargeted, the optic fiber/GRIN lens was detached, or there were serious health problems unrelated to the protocol; (2) in the chronic corticosterone (CORT) experiment, 2 CORT-treated and 3 CORT/Stim×10-treated mice died shortly after the open field test due to CORT-related debilitation, and analyses excluding these animals are reported in Extended Data Fig. 17 and Supplementary Result 3; (3) for RNA-seq, genes with FPKM < 0.1 were excluded to avoid inflated FDR; (4) for ATAC-seq, peaks overlapping the ENCODE blacklist were excluded; (5) for population decoding analyses, sessions with fewer than 10 simultaneously recorded neurons were excluded. Any case-by-case exclusion is stated in the corresponding figure legend.
Replication	Reproducibility was supported by independent biological replicates (n ≥ 3 per group) within each experiment, and by cross-modality convergence of findings (RNA-seq, ATAC-seq, immunostaining, and calcium imaging all pointing to the same conclusions). The antidepressant-like effect was independently reproduced in the chronic corticosterone model (Extended Data Fig. 17). Large-scale replication across two fully independent cohorts was not performed for every assay; however, representative sequencing-based, behavioural and histological

	experiments were repeated to confirm that the procedures worked as intended.
Randomization	Mice of the same age and sex, housed under identical conditions, were randomly allocated to control/non-stimulated, virus-only, or virus + stimulation groups.
Blinding	Complete blinding during data collection was not always possible because experimenters had to control the optogenetic stimulation for each animal. To minimize bias, behavioral data were acquired and quantified automatically using ImageJ-based plugins, and imaging data were processed with predefined pipelines (IDPS, Huygens + MATLAB). When manual quantification was required, group IDs were concealed as far as feasible.

Reporting for specific materials, systems and methods

We require information from authors about some types of materials, experimental systems and methods used in many studies. Here, indicate whether each material, system or method listed is relevant to your study. If you are not sure if a list item applies to your research, read the appropriate section before selecting a response.

Materials & experimental systems

n/a	Involved in the study
☐	☒ Antibodies
☒	☐ Eukaryotic cell lines
☒	☐ Palaeontology and archaeology
☐	☒ Animals and other organisms
☐	☒ Clinical data
☒	☐ Dual use research of concern
☒	☐ Plants

Methods

n/a	Involved in the study
☒	☐ ChIP-seq
☒	☐ Flow cytometry
☒	☐ MRI-based neuroimaging

Antibodies

Antibodies used	Primary antibodies used:<br>Calbindin (rabbit, polyclonal, 1:1,000; Synaptic Systems, Cat# 214 002, RRID:AB_2068199).<br>Calbindin (goat, polyclonal, 1:500; Frontier Institute, Cat# MSFR100410, RRID: AB_2571569).<br>Cyclin B (V152) (mouse, monoclonal, 1:500; Thermo Fisher Scientific, Cat# MA1-155, RRID:AB_2536863).<br>Phospho-Histone H3 (Ser10) (rabbit, polyclonal, 1:500; Millipore, Cat# 06-570, RRID:AB_310177).<br>HA-tag (rabbit, polyclonal, 1:500; Cell Signaling Technology, Cat# 3724, RRID:AB_1549585).<br>ΔFosB (D3S8R) (rabbit, monoclonal, 1:1,000; Cell Signaling Technology, Cat# 14695, RRID:AB_2798577).<br>NeuN (mouse, monoclonal, 1:500; Millipore, Cat# MAB377X, RRID:AB_2149209).<br>GFAP (rabbit, polyclonal, 1:500; Sigma-Aldrich, Cat# G9269, RRID:AB_477035).<br>Iba1 (rabbit, polyclonal, 1:500; FUJIFILM Wako, Cat# 019-19741, RRID:AB_839504).<br>JunD (rabbit, polyclonal, 1:500; Abcam, Cat# ab28837, RRID:AB_2130167).<br>Tri-methyl-Histone H3 (Lys9) (H3K9me3) (mouse, monoclonal, 1:500; FUJIFILM Wako, Cat# MABI0308, RRID: AB_2887767)<br>"Lamin B1 (mouse, monoclonal, 1:200; Thermo Fisher Scientific, Cat# MA1-06103, RRID:AB_2281281)."<br><br>Secondary antibodies:<br>Fluorophore-conjugated anti-rabbit and anti-mouse secondary antibodies (Alexa Fluor series; Thermo Fisher Scientific).
Validation	All antibodies were used according to the manufacturers' datasheets. For widely used markers (e.g., Calbindin (rb), Calbindin (goat), NeuN, GFAP, Iba1), specificity has been established in the literature and was confirmed in our hands by the expected labeling pattern in mouse hippocampus. The ΔFosB antibody specifically recognises the ΔFosB isoform without cross-reactivity to full-length FosB, as verified by both manufacturer validation and independent reports. For less commonly used antibodies, specificity was assessed by optimizing working dilutions, antigen retrieval methods, and blocking conditions to minimize non-specific staining and preserve the expected cellular/subcellular localization.

Animals and other research organisms

Policy information about studies involving animals; ARRIVE guidelines recommended for reporting animal research, and Sex and Gender in Research

Laboratory animals	Species: Mus musculus (mouse)<br>Strain: ROSA26-CAG-stopflox-ChR2(H134R)-EYFP (Strain#012569; RRID:IMSR_JAX:012569) and POMC-Cre (Strain #010714; RRID:IMSR_JAX:010714)<br>Source: Jackson Laboratories (Bar Harbor, Maine); F1 POMC-Cre::ChR2-EYFP mice generated in-house by crossing the two strains.<br>Age: 3–4 months for RNA-seq and ATAC-seq experiments; 6–12 months for other experiments<br>Health status: specific pathogen-free (SPF)
Wild animals	No wild animals were used in this study.

Reporting on sex	Mice of both sexes were used for behavioural and histological experiments. For these experiments, sex was included as a covariate in ANCOVA analyses where appropriate (e.g., Fig. 3e and Supplementary Result 3), and sex-disaggregated values for individual experiments are provided in the Source Data file. Sequencing experiments (RNA-seq/ATAC-seq) were performed in males only to avoid potential oestrous-cycle confounds; this single-sex design is acknowledged as a limitation. Randomization was conducted within age- and sex-matched cohorts.
Field-collected samples	No field-collected specimens were used in this study.
Ethics oversight	All animal procedures were approved by the Fujita Health University Institutional Animal Care and Use Committee (Approval No. APU22016, renewed as APU25150) and were conducted in accordance with institutional policies and applicable national guidelines for the care and use of laboratory animals.

Note that full information on the approval of the study protocol must also be provided in the manuscript.

Clinical data

Policy information about clinical studies
All manuscripts should comply with the ICMJE guidelines for publication of clinical research and a completed CONSORT checklist must be included with all submissions.

Clinical trial registration	All human-related data used here were secondary datasets downloaded from public repositories, specifically the Sequence Read Archive (SRA; SRP241159). No primary clinical data were collected by the authors.
Study protocol	All human-related data used here were secondary datasets downloaded from public repositories, specifically the Sequence Read Archive (SRA; SRP241159). No primary clinical data were collected by the authors.
Data collection	All human-related data used here were secondary datasets downloaded from public repositories, specifically the Sequence Read Archive (SRA; SRP241159). No primary clinical data were collected by the authors.
Outcomes	All human-related data used here were secondary datasets downloaded from public repositories, specifically the Sequence Read Archive (SRA; SRP241159). No primary clinical data were collected by the authors.

Plants

Seed stocks	This study did not use plants, plant-derived materials.
Novel plant genotypes	This study did not use plants, plant-derived materials.
Authentication	This study did not use plants, plant-derived materials.
