# Supplementary material for: Repetitive neuronal activation regulates cellular maturation state via nuclear reprogramming
Source: Nat Commun. 2026 Jul 17;17:5881. doi: 10.1038/s41467-026-74202-w (PMC13379389; doi:10.1038/s41467-026-74202-w)
Supplement: Supplementary file 6 — Transparent Peer Review file [file 41467_2026_74202_MOESM6_ESM.pdf]

nature portfolio

Peer Review File

Repetitive Neuronal Activation Regulates Cellular Maturation State via Nuclear Reprogramming

Corresponding Author: Professor Tsuyoshi Miyakawa

This file contains all reviewer reports in order by version, followed by all author rebuttals in order by version.

Version 0:

Reviewer comments:

Reviewer #1

(Remarks to the Author)
This manuscript by Murano and colleagues investigates the cellular mechanisms underlying brain stimulation therapies like electroconvulsive therapy (ECT). The authors introduce an optogenetic stimulation model in mice—termed REPOPS—targeting dentate gyrus granule cells to mirror ECT. They show that chronic, repeated neuronal stimulation (5 minutes per day over 10 days) leads to a persistent dematuration of neurons. This dematuration is evidenced by changes in gene expression profiles, chromatin and nuclear reorganization, and behavioral alterations. Notably, this state is associated with reduced spatial information coding and enhanced encoding of locomotor speed. Mechanistically, the authors implicate the reactivation of G2/M cell cycle-related genes, particularly Cyclin B, in mediating these long-term effects on DNA structure, maturation status, and behavior.

Overall, this study presents a compelling connection between neuronal stimulation and reprogramming, linking molecular and structural changes to behavioral outcomes. However, the manuscript would benefit from several clarifications and revisions before being suitable for publication.

Introduction:
- The authors should provide more detailed information regarding the molecular and cellular hallmarks of hippocampal neuron dematuration. Specific examples (e.g., gene markers, structural changes, or previously established pathways) are necessary to frame the biological significance of the work. This is especially important given the central aim of the study: elucidating the cellular mechanisms underlying ECT-induced dematuration.

- The introduction does not specify the experimental models (e.g., rodents, humans, or in vitro systems) used in studies referenced. Clarifying the model systems will help the reader understand the relevance of the findings.

- While the authors have a strong track record in the field, the introduction currently includes an excessive number of self-citations. A clearer distinction should be made between the authors' prior work and findings from other research groups to provide a more balanced and contextualized overview of the field.

Results:

- Figure 1C (RNAseq Data):
The heatmap and associated data emphasize the “quantity” of differentially expressed genes following Stimx10. However, the identity of these changes are more informative. The authors should conduct a comparative analysis of differentially expressed genes between the two time points and highlight those with the most significant functional or mechanistic relevance.

- Figure 2B (Timeline Clarification):
The timeline presented for the Stimx3 group is potentially misleading. Since the authors delayed stimulation in this group for tissue collection purposes, the current depiction misaligns with the Stimx10 group. For accurate interpretation, comparisons should align both groups by stimulation day. For instance, locomotor changes on day 9 in Stimx3 should be compared to day 2 of Stimx10.

- Figure 2C (Locomotor Activity):
It is unexpected that the Stimx3 group does not show increased locomotion, given that the same stimulation protocol induces hyperactivity during the early phase of Stimx10. The authors should address this apparent discrepancy and provide a mechanistic or methodological explanation.

- Figure 2D (Behavioral Tests):
The manuscript should clearly indicate in the Results section when the Tail Suspension Test (TST) and Forced Swim Test (FST) were conducted in relation to the stimulation timeline.

-Extended Figure 2:
The data presented in Extended Figure 2 are central to understanding the therapeutic potential of brain stimulation. These findings should be integrated into the main figures to enhance the impact and clarity of the results.

- Immunofluorescence quantification:
The statistical analyses involving immunofluorescence data are not appropriately conducted. The reported "n" reflects the number of cells rather than biological replicates (i.e., number of animals). This is not statistically valid, as it inflates the sample size and underestimates variability. The authors should reanalyze these datasets using the number of mice as the unit of analysis.

Discussion:

- References 57 and 58 do not substantiate the claims made in the corresponding sentence. These should be removed or replaced with more appropriate citations.

- Several studies have reported that neuronal dematuration can have detrimental effects on brain function and neuronal activity. The authors should address this apparent discrepancy by discussing why, in their model, dematuration is associated with seemingly beneficial behavioral outcomes. In particular, they should consider the timing of behavioral assessments, as this could significantly influence the observed effects (see earlier comment regarding the timeline).

- While the authors specifically targeted neurons in their manipulation, the RNA-seq analysis does not distinguish between neuronal and glial differentially expressed genes (DEGs). Given the emerging role of glial cells in brain plasticity and rejuvenation, the inability to isolate neuron-specific transcriptomic changes represents a limitation. The authors should acknowledge this and briefly discuss the potential contribution of glial responses to the observed effects.

Conclusion:

This manuscript presents innovative and valuable insights into the cellular and behavioral consequences of repeated neuronal stimulation. However, important clarifications, data reanalyses, and structural improvements are needed to enhance its clarity, scientific rigor, and overall impact.

Reviewer #2

(Remarks to the Author)
This study uses a novel optogenetic neuronal stimulation of the dentate gyrus to model neural stimulation therapies such as ECT and TMS, which are effective treatments for a variety of psychiatric disorders. The authors demonstrate that repeated application of this stimulation (REPOPS) induces a cellular dematuration phenotype and elegant mechanistic work is utilized to implicate Cyclin B, nuclear structure plasticity and elevation of FosB in persistent transcriptional changes with neural stimulation. The most significant limitation of the study is that assays for depression-related behaviors are confounded by a hyperlocomotive phenotype induced by REPOPS, making it difficult to determine whether the observed changes in cellular state or neural coding are relevant for therapeutic effects of neural stimulation therapies.

Strengths:
The mechanistic analyses (RNAseq, ATAC-seq, immunostaining, cyclin B knockdown with CRISPR) are high quality and convincingly elucidate a pathway by which REPOPS induces long-term changes in gene expression via cyclin B and FosB.

The paper makes nice use of RNAseq data from human postmortem brains to demonstrate that patients with bipolar or unipolar depression who have been treated with ECT show an immature-like gene expression pattern when compared to those who did not undergo ECT, which does suggest that some molecular phenotypes associated with ECT can be recapitulated with this model.

The use of in vivo calcium imaging in freely moving mice to evaluate changes in neural coding of navigational information with REPOPS is technically sophisticated and data quality is good.

Although it is not clear that REPOPS is a valid model of ECT specifically, it does provide a valuable tool to test how various parameters of neural stimulation modify cellular phenotypes, neural coding and behavior.

The mechanistic outline in figure 6D is much appreciated

Weaknesses:
It's arguable that the REPOPS model, which delivers local stimulation to DG granule cells is a valid model for ECT, which induces generalized seizures. It would be more appropriate to frame this as a neurostimulation protocol more generally.

The behavioral analyses (Figure 2) used to assess antidepressant-like effects (FST, TST) unfortunately are confounded by the hyperlocomotive phenotype induced by the stimulation. Additional assays that are less reliant on locomotor outputs such as sucrose preference or novelty suppressed feeding could be considered.

In establishing the model (Figure 1), it would be helpful to include a control virus to demonstrate whether changes in cellular maturity are mediated through specific activation of GC's vs nonspecific effects of optical stim of the region.

Reviewer #3

(Remarks to the Author)
The authors perform an ambitious and important study to understand the molecular mechanisms underlying electroconvulsive therapy (ECT). To simulate the effects of ECT in mice, they developed a repeated optogenetic stimulation (REPOS) protocol delivered to granule cells in the dentate gyrus (DG) of adult mice. REPOS repeated for 10 days, compared to 3 days and no stimulation, induced lasting changes (up to 2 weeks) in gene expression. Many of these genes were associated with cell division pathways and overlapped with expression profiles of healthy infant mice, suggesting that REPOS induced immature-like gene expression patterns in adult mice. An overlap of differentially expressed genes between ECT-treated patients (with bipolar disorder or depression) versus untreated patients, and those altered during early versus late hippocampal development, further supported the idea that REPOS, like ECT, may induce neuronal dematuration.

Behaviorally, 10-day REPOS enhanced speed-related activity in DG neurons, increased locomotor activity, and reduced immobility in depression-related behavioral assays such as the forced swim test (FST) and tail suspension test (TST). However, REPOS also downregulated synaptic genes, impaired positional coding and spatial map formation in the DG, and significantly disrupted contextual memory and social behavior. Finally, the authors performed mechanistic studies linking REPOS to G2/M cell cycle re-entry and nuclear structure reorganization, suggesting calcium signaling and activity-dependent factors, specifically ΔFosB, as candidate drivers of the immature-like state.

While the breadth of experiments is impressive, there are several major caveats and minor areas that require clarification:

Major Points:
1. Confounding locomotor effects in behavioral assays: Since REPOS induces speed-activity encoding in DG and significantly enhances locomotor activity, it is difficult to tease-apart whether the observed effects on FST/TST are simply an outcome of increased locomotion or an actual anti-depressant behavior. I think the reduction in immobility in FST and TST after REPOS likely reflects increased locomotor activity, rather than a true antidepressant-like effect. Standard practice is to control for locomotor activity when interpreting these assays. Therefore, the conclusion that REPOS reduces depression-like behavior is speculative and potentially misleading. Moreover, the observed impairment in social interaction contradicts the interpretation of reduced depression-like behavior. The authors could reanalyze immobility after adjusting for locomotor activity, or test whether FST/TST behavior is rescued in mice with Cyclin B knockdown, as they did for locomotor activity. If not possible, the authors should tone down their claim of 'REPOS inducing long-term anti-depressant effects' and explicitly acknowledge this as a strong limitation of their study.

2. Lack of disease-relevant models: The authors aim to model ECT, which is used clinically for patients with psychiatric disorders, yet they only test REPOS in healthy mice. To strengthen the translational value of this study, REPOS should be tested in mouse depression-like models such as chronic variable stress or social defeat stress model. Demonstrating that REPOS reduces depression-like behavior in stressed mice would provide stronger evidence that REPOS mimics ECT's therapeutic and molecular effects.

3. Cell-type heterogeneity in RNA-seq: Bulk RNA-seq and ATAC-seq of DG tissue includes multiple cell types other than granule cells (GC), the main target cells of this study. I highly recommend using an external single-cell RNA-seq dataset of mouse hippocampus (e.g. PMID: 37034735, PMID: 38217668, https://portal.brain-map.org/atlases-and-data/rnaseq/mouse-wholecortex-and-hippocampus-smart-seq) to adjust cell-type composition in differential models and use deconvolution approaches to further clarify whether dematuration signatures are specific to GC.

4. ATAC-seq quality control: No QC metrics are shown for ATAC-seq. Please provide transcription start site (TSS) enrichment plots, fragment-in-peaks (FRiP) scores, and details on peak merging and peak reproducibility across samples. Please provide details on which method or pipeline was used for transcription factor motif enrichment of differential ATAC-seq peaks in the Methods section.

5. ATAC-seq interpretation: The ATAC-seq results are not integrated with RNA-seq findings. Differential peaks should be assigned to genes (e.g. preferably using Hi-C promoter-enhancer connections or other gene assignment methods). Pathway analysis of peak-associated genes could reveal consistency with RNA-seq pathways (e.g. cell cycle/mitosis). Statistical comparison between ATAC- and RNA-seq derived gene sets should be done to provide deeper insights into chromatin-transcriptome relationships induced by REPOS.

6. Sex specific analysis: It is not clear whether the experiments performed in this study were using male or female mice or both. If experiments performed were only in the male mice, it would be a missed opportunity to not test molecular and behavioral effects of REPOS in female mice. This is important because psychiatric disorders tend to effect males and females differently. I highly recommend performing at least some of the same experiments (e.g. Calbindin expression, locomotor activity, RNA-seq) to show consistent outcomes in female mice as well.

7. Comparison to patient ECT data: The authors only compared a subset of selected synaptic genes between REPOS and ECT-treated patients. A more comprehensive comparison of differential gene expression between 10-day REPOS vs non-stimulated mice and ECT-treated vs untreated patients is required to provide unbiased insight into whether REPOS truly parallels ECT at the molecular level.

8. G2/M 're-entry' in mature neurons: I think the authors claim of REPOS induced G2/M cellcycle 're-entry' is too strong without further functional evidence (e.g., showing a full panel of mitotic markers (e.g., Ki67, Aurora kinase B, PCNA) or markers of DNA synthesis (e.g., BrdU). Was pH3 staining co-localized with neuronal markers (NeuN, Prox1) to confirm this occurs in mature neurons and not in glial or progenitor cells? Does cyclin B+, lamin-disruption, and pH3+ neurons overlap spatially? This co-localization would strengthen the argument. Currently, it may be more accurate to say, "REPOS upregulates G2/M phase associated genes" or "induces a G2/M-like transcriptional signature." If neurons truly re-entered G2/M transcriptional programs, why was there no evidence of apoptosis detected in TUNEL assay? Aberrant cell cycle re-entry is often associated with neuronal death. For example, aberrant cell cycle re-entry has been shown as a causal phenomenon in neurodegeneration and Alzheimer's disease (PMID: 20584423). This discrepancy requires further investigation and deeper discussion.

9. Adult neurogenesis markers: Authors conclude cyclin B is expressed in mature neurons rather than newborn cells. It would be valuable to test whether DE genes from REPOS overlap with known markers of adult hippocampal neurogenesis (e.g. doublecortin, nestin, Pax6, NeuroD, calretinin) and perform immunostaining of cyclin B with markers of neurogenesis. This would be important to help distinguish whether REPOS mimics early developmental dematuration or induces adult neurogenesis programs.

10. Additional cell-type markers: While NeuN, GFAP, and Iba1 staining was performed, the analysis would benefit from including inhibitory neuron markers (PV, SST) and excitatory subtypes (granule cells, mossy cells) to clarify which neuronal populations are additionally affected by REPOS treatment in mice.

Minor Points:
1. Gene overlap analyses (Fig. 1D and 1F): Currently, the authors use Fisher's test on nominally significant genes ($p < 0.05$). I recommend a rank-rank hypergeometric overlap (RRHO) analysis, which is threshold-free and more powerful, allowing the entire ranked gene list to be compared. The Fisher's test could still be included, but I would also like to see the overlap after multiple-comparison correction for each dataset (e.g. FDR 5%).
2. Long-term Calbindin expression: Calbindin expression was tested up to 2 weeks poststimulation. It would be valuable to extend the analysis to later time points (e.g., up to 1 month) to assess whether immature-like neuronal state is transient or persistent.
3. RNA-seq and ATAC-seq analysis: Important details are missing in the Methods section. Please specify the sample sizes, how genes/peaks were filtered, and whether covariates (e.g. RNA integrity number, sex) were included in differential analyses. In addition, the github code does not include the RNA/ATAC-seq differential analysis code, which should be open source for reproducibility.
4. Cell death assay: Although TUNEL staining showed no apoptosis, RNA-seq identified enrichment of 'positive regulation of cell death.' This discrepancy suggests transcriptional

activation of stress/apoptosis pathways without execution. The authors should check whether the DE genes are upstream regulators (stress response, pro-apoptotic signaling) or execution markers (caspases, BAX/BAK). Validation with Annexin V staining or caspase assays would clarify whether early apoptosis is occurring.
5. Mouse age discrepancy: RNA-seq and ATAC-seq were performed in 3-4 month old mice, while other experiments used 6-12 month old mice. The rationale for this discrepancy should be clarified, as age strongly influences gene expression and behavior.
6. Sample size inconsistencies: Please provide sample size for each experiment. It seems like different experiments use inconsistent group sizes (e.g. N=6 for RNA-seq, N=4 for ATAC-seq, N=10 for locomotor and FST/TST, N=7 for social interaction). The rationale should be explained, and power calculations would increase confidence.
7. Parallels with ECT: Typically, ECT does not induce chronic hyperlocomotion in humans. The persistent hyperactivity seen in mice raises concerns about how well REPOPS models therapeutic effects of ECT. Please explain this in Discussion section
8. 3-day and 10-day REPOPS: The rationale for these timeframes is not fully justified. Why 3 and 10? Do these correspond to clinical ECT regimens (e.g., acute vs maintenance treatments)? Please clarify why and how these timepoints were selected.
9. Knockout efficiency: The authors state cyclin B expression was "effectively eliminated," but I was not able to find quantitative data to support this (e.g., reduction in RNA or protein levels). Was knockout uniform across the DG or mosaic? Did authors validate specificity with sequencing or off-target prediction methods?
10. Specificity of detection: Since ΔFosB is a truncated isoform, the authors must clarify whether their antibody or probe distinguishes ΔFosB from full-length FosB. Without isoformspecific validation, claims about ΔFosB may be confounded.
11. Lack of transparency in ATAC-seq analysis: Authors mention that the signal maps and peak locations were used as input data for Active Motif's proprietary analysis program. While I understand that the authors used Active Motif's proprietary program, it is important for transparency and reproducibility to include more information on what algorithms and parameters were used for peak calling and gene annotation.

Reviewer #4

(Remarks to the Author)
I co-reviewed this manuscript with one of the reviewers who provided the listed reports. This is part of the Nature Communications initiative to facilitate training in peer review and to provide appropriate recognition for Early Career Researchers who co-review manuscripts.

Version 1:

Reviewer comments:

Reviewer #2

(Remarks to the Author)
This study uses a novel optogenetic neuronal stimulation of the dentate gyrus to model neural stimulation therapies such as ECT and TMS, which are effective treatments for a variety of psychiatric disorders. The authors demonstrate that repeated application of this stimulation (REPOPS) induces a cellular dematuration phenotype and elegant mechanistic work is utilized to implicate Cyclin B, nuclear structure plasticity and elevation of FosB in persistent transcriptional changes with neural stimulation. They also provide some evidence that this protocol reduces depression-related behaviors in both stress-naïve mice and a chronic corticosterone depression model, though the effect is not seen in all assays and the interpretation of some behavioral tests is confounded by hyperlocomotion. The revised manuscript is strengthened by additional behavioral testing in a depression model, new behavioral tests with fewer locomotor confounds, additional cell-type enrichment analysis of RNAseq data that point to granule cells and neuroblasts as the drivers of transcriptional changes observed, and immunohistochemistry for markers of neurogenesis as a pertinent negative and more extended timepoints to assess durability of dematuration phenotype. The authors have put in substantial new work to largely address reviewer concerns from the initial submission.

Strengths:
The mechanistic analyses (RNAseq, ATAC-seq, immunostaining, cyclin B knockdown with CRISPR) are high quality and convincingly elucidate a pathway by which REPOPS induces long-term changes in gene expression via cyclin B and FosB.

The paper makes nice use of RNAseq data from human postmortem brains to demonstrate that patients with bipolar or unipolar depression who have been treated with ECT show an immature-like gene expression pattern when compared to those who did not undergo ECT, which does suggest that some molecular phenotypes associated with ECT can be recapitulated with this model.

The use of in vivo calcium imaging in freely moving mice to evaluate changes in neural coding of navigational information with REPOPS is technically sophisticated and data quality is good.

Although it is not clear that REPOPS is a valid model of ECT specifically, it does provide a valuable tool to test how various parameters of neural stimulation modify cellular phenotypes, neural coding and behavior.

New behavioral testing strengthens claim that REPOPS increases therapeutically relevant behaviors, specifically in the NSFT, though no effect is seen in the SPT. Demonstration of a behavioral effect in a depression model is also a valuable addition.

New cell-type enrichment analysis of RNAseq data provides evidence of cell-type specificity of transcriptional changes and points to granule cells and neuroblasts as the drivers of transcriptional changes observed.

New integration of ATAC-seq and RNA-seq datasets shows concordance between these datasets, supporting validity of claims.

Additional IHC for neurogenesis markers shows no change with REPOPS, supporting claim of a dematuration-like effect rather than induction of adult neurogenesis program.

The addition of an extended timepoint (40d) for calbindin expression supports a durable effect of REPOPS.

Weaknesses:
Although the behavioral effects of REPOPS in the NSFT are convincing and nicely replicated in a depression model, no effect is seen in the SPT, and the FST and TST results remain confounded by hyperlocomotion. I would suggest moving the NSFT to Figure 3 in place of the FST or TST as this provides the clearest evidence for a therapeutically-relevant behavioral effect. The authors may consider addressing whether this model impacts specific depression-related behavioral phenotypes and not others, and whether these differential effects are related to the regional specificity of their neuronal stimulation.

The abstract states "…observed ECT-like behavioral effects, including decreased depression-like behavior and increased locomotor activity". Although the authors cite a single study of 15 patients in which psychomotor activity was increased in patients treated with ECT who achieved remission, to frame hyperlocomotion as an accepted therapeutic effect of ECT is an overstatement.

Minor comments:
Extended Data Figure 17b- the mice that died soon after behavioral assessments should be excluded from statistical analysis rather marked on the graph as the 3 in the REPOPS group exhibit a marked reduction in locomotor activity and are likely artificially reducing the group average.

(Remarks on code availability)

Reviewer #3

(Remarks to the Author)
I commend the authors for thoroughly responding to all my comments and feedback. They have incorporated all my suggestions and have performed sufficient new experiments to validate their findings, including behavioral experiments to show that REPOS-related effects are not solely due to increased locomotor activity. They have deconvoluted their gene-expression data using single-cell dataset and identified cell-types enriched for REPOS-associated genes. They have now provided sufficient information on their ATAC/RNA-seq methodology. Overall, this is an interesting and important work, and I congratulate the authors on this manuscript. I have no further comments or feedback.

(Remarks on code availability)

Reviewer #4

(Remarks to the Author)
I co-reviewed this manuscript with one of the reviewers who provided the listed reports. This is part of the Nature Communications initiative to facilitate training in peer review and to provide appropriate recognition for Early Career Researchers who co-review manuscripts.

(Remarks on code availability)

Version 2:

Reviewer comments:

Reviewer #2

(Remarks to the Author)

The authors have done an excellent job in incorporating all feedback and I have no additional comments or concerns.

(Remarks on code availability)

Open Access This Peer Review File is licensed under a Creative Commons Attribution 4.0 International License, which permits use, sharing, adaptation, distribution and reproduction in any medium or format, as long as you give appropriate credit to the original author(s) and the source, provide a link to the Creative Commons license, and indicate if changes were made.
In cases where reviewers are anonymous, credit should be given to 'Anonymous Referee' and the source.
The images or other third party material in this Peer Review File are included in the article's Creative Commons license, unless indicated otherwise in a credit line to the material. If material is not included in the article's Creative Commons license and your intended use is not permitted by statutory regulation or exceeds the permitted use, you will need to obtain permission directly from the copyright holder.
To view a copy of this license, visit https://creativecommons.org/licenses/by/4.0/

Reviewer #1

Comment 1

The authors should provide more detailed information regarding the molecular and cellular hallmarks of hippocampal neuron dematuration. Specific examples (e.g., gene markers, structural changes, or previously established pathways) are necessary to frame the biological significance of the work. This is especially important given the central aim of the study: elucidating the cellular mechanisms underlying ECT-induced dematuration.

Response:

We appreciate the reviewer's insightful comment emphasizing the need to specify the molecular and cellular hallmarks of hippocampal neuron dematuration.

To clarify this point, we added expression data for representative genes—Tdo2 and Dsp (mature markers) and Drd1 and Grp (immature markers)—which were previously shown to change during DG development (0.45-, 0.33-, 3.6-, and 1.3-fold; postnatal day 8 vs 33 weeks) (Murano et al., *Commun. Biol.* 2019). These developmental markers have also been confirmed to change in other mouse models with immature-like DG, including αCaMKII$^{+/-}$ (Yamasaki et al., *Mol. Brain* 2008; Shin et al., Bipolar Dis., 2013), fluoxetine-treated (Kobayashi et al., *PNAS* 2010), and calcineurin KO mice (Hagihara et al., *Mol. Brain* 2022).

After 10 days of REPOPS, mature markers significantly decreased while immature markers increased (Extended Data Fig. 7b), indicating a transcriptional shift toward an immature neuronal state. These results provide direct molecular evidence supporting REPOPS-induced dematuration of the DG.

Change:

[Original: ---]

[Revised: Extended Data Fig. 7b] added

b

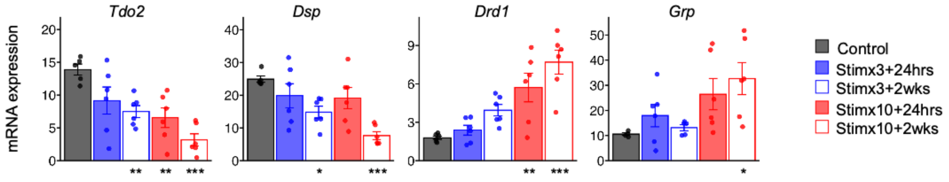

[Original: Extended Data Fig. 4, legend]

Extended Data Figure 4. Group analysis of altered gene expression following REPOPS

Gene Ontology (GO) analysis …

[Revised: Extended Data Fig. 7b, legend]

Extended Data Figure 7. Expression analysis of transcriptional changes following REPOPS.

b, Expression levels of representative markers of mature (Tdo2, Dsp) and immature (Drd1, Grp) DG neurons, which were previously shown to change during development (2.2-, 3.0-, 0.28-, and 0.77-fold; postnatal day 8 vs 33 weeks) (ref: Murano et al., Commun. Biol., 2019), are shown. One-way ANOVA followed by Tukey's post hoc test; asterisks indicate significance vs. control (*$P < 0.05$, **$P < 0.01$, ***$P < 0.001$).

Comment 2

The introduction does not specify the experimental models (e.g., rodents, humans, or in vitro systems) used in studies referenced. Clarifying the model systems will help the reader understand the relevance of the findings.

Response:

We agree that specifying the experimental models cited in the Introduction improves the clarity and relevance of our study. Accordingly, we have revised the text to specify whether each referenced study used human, rodent, or in vitro models, thereby providing clearer context for how previous findings relate to our work.

Change:

[Original: L73]

ECT promotes neuroplastic changes, such as increased adult neurogenesis in the dentate gyrus and hippocampal volume enlargement[34–36]

[Revised: L73]

ECT in patients promotes neuroplastic changes, such as increased adult neurogenesis in the dentate gyrus and hippocampal volume enlargement[31–33]

[Original: L78]

Similar changes occur with chronic administration of fluoxetine, a selective serotonin reuptake inhibitor (SSRI)[9,39,40].

[Revised: L78]

Similar changes occur with chronic administration of fluoxetine, a selective serotonin reuptake inhibitor (SSRI), in mouse models[36–38,35].

[Original: L81]

Likewise, other noninvasive brain stimulation methods, such as repetitive transcranial magnetic stimulation (rTMS) and transcranial direct current stimulation (tDCS), can induce neuroplastic changes in the brain and have promising therapeutic potential [42,43].

[Revised: L82]

Likewise, other noninvasive brain stimulation methods, such as repetitive transcranial magnetic stimulation (rTMS) and transcranial direct current stimulation (tDCS), can induce neuroplastic changes in patients' brains and have promising therapeutic potential[41,42].

Comment 3

While the authors have a strong track record in the field, the introduction currently includes an excessive number of self-citations. A clearer distinction should be made between the authors' prior work and findings from other research groups to provide a more balanced and contextualized overview of the field.

Response:

We appreciate the reviewer's comment and agree that reducing self-citations improves the balance of the Introduction. Accordingly, we have removed several self-citations and incorporated additional references from other research groups to provide a more balanced and contextualized overview of the field.

Change:

[Original: Reference] removed

15, Hagihara et al., Molecular Brain, 2022

20, Hagihara et al., Molecular Brain, 2014

40, Ohira et al, Molecular Brain, 2019

[Revised: Reference] added

37, Segi-Nishida Bio Pharm Bull, 2011

38, Imoto et al., Mol Brain, 2015

Comment 4

Figure 1C (RNAseq Data): The heatmap and associated data emphasize the “quantity” of differentially expressed genes following Stimx10. However, the identity of these changes are more informative. The authors should conduct a comparative analysis of differentially expressed genes between the two time points and highlight those with the most significant functional or mechanistic relevance.

Response:

We agree that identifying and interpreting the functional relevance of differentially expressed genes is essential for understanding the underlying mechanisms. We therefore directly compared Stim×3 and Stim×10 at both 24 hours and 2 weeks in Extended Data Figure 7c. These additional analysis highlights key pathway-level differences between the two conditions.

Change:

[Original: Extended Data Fig. 4] ---

[Revised: Extended Data Fig. 7c] panel 7c was added

c

Stimx10+24hrs vs. Stimx3+24hrs (Fig1c, left)

	Biogroup name	common genes	direction	p-value
1	nuclear envelope	35	down	2.90E-23
2	angiogenesis	20	up	2.90E-18
3	blood vessel morphogenesis	21	up	4.50E-17
4	cell morphogenesis involved in neuron differentiation	24	down	5.60E-16
5	mitochondrion organization	25	up	7.00E-16
6	transcription coregulator activity	28	down	1.20E-15
7	DNA repair	26	down	3.70E-15
8	regulation of intracellular transport	25	down	5.40E-15
9	cell part morphogenesis	25	down	1.90E-14
10	extracellular matrix	14	up	2.70E-14

Stimx10+2wks vs. Stimx3+2wks (Fig1c, right)

Rank	Biogroup name	common genes	direction	p-value
1	presynapse	102	up	9.00E-76
2	distal axon	89	up	4.90E-64
3	ribonucleoprotein complex biogenesis	83	down	5.30E-51
4	mitochondrial inner membrane	81	up	6.90E-50
5	cell part morphogenesis	84	up	3.00E-45
6	nuclear outer membrane-endoplasmic reticulum membrane network	84	up	4.00E-45
7	endoplasmic reticulum membrane	82	up	6.60E-45
8	ATP metabolic process	50	up	1.20E-41
9	DNA repair	73	down	1.70E-41
10	secretion	79	up	1.70E-40

[Original: Extended Data Fig. 4, legend]

Extended Data Figure 4. Group analysis of altered gene expression following REPOPS

Gene Ontology (GO) analysis ...

[Revised: Extended Data Fig. 7c, legend]

Extended Data Figure 7. Expression analysis of transcriptional changes following REPOPS.

a, Gene Ontology (GO) analysis …

b, Expression levels of representative…

c, Pathway analysis comparing Stim×3 and Stim×10 at both 24 hours and 2 weeks after the last stimulation. "Nuclear envelope" was among the top downregulated categories at 24 hours, whereas "presynapse" was prominently altered at 2 weeks.

**Comment 5**
Figure 2B (Timeline Clarification): The timeline presented for the Stimx3 group is potentially misleading. Since the authors delayed stimulation in this group for tissue collection purposes, the current depiction misaligns with the Stimx10 group. For accurate interpretation, comparisons should align both groups by stimulation day. For instance, locomotor changes on day 9 in Stimx3 should be compared to day 2 of Stimx10.

Response:

We thank the reviewer for this comment and acknowledge that the original timeline could be misleading. To improve clarity, we have prepared an alternative version that aligns the Stim×3 and Stim×10 groups by stimulation day. The revised figures are now provided as Extended Data Figure 5a and 5b.

Change:

[Original: Fig. 2b, c] retained

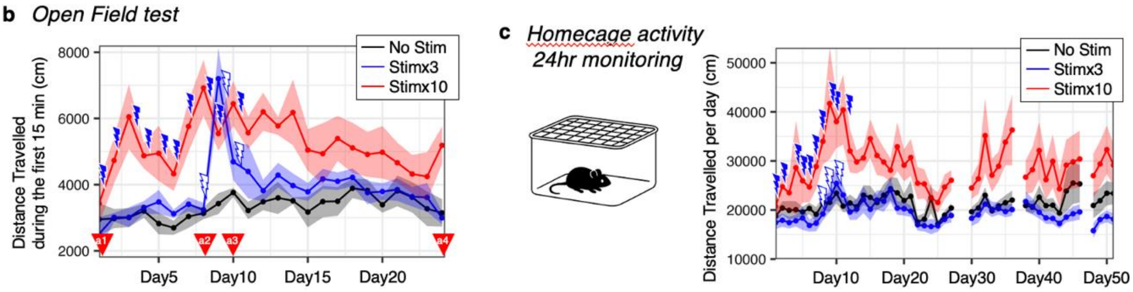

[Revised: Extended Data Fig. 5a, b] added.

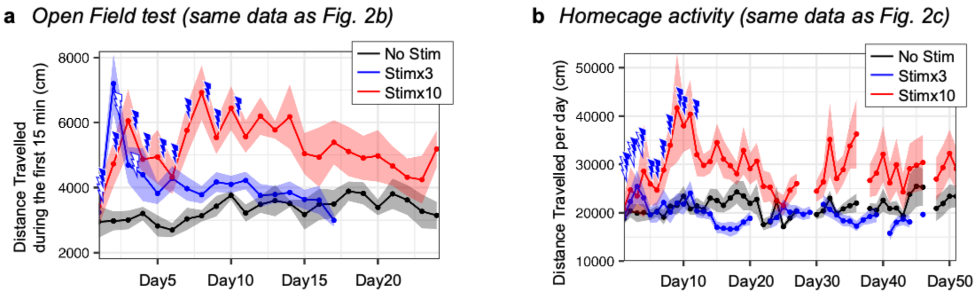

[Original: ---]

[Revised: Extended Data Fig. 5a,b, legend]

Extended Data Figure 5. REPOPS for 10 days induces anti-depressive behavioral changes in mice.

a, Open field test. Same data as in Figure 3b, but realigned by the start of the stimulation period.

b, Home cage activity. Same data as in Figure 3c, but realigned by the start of the stimulation period**.**

Comment 6

Figure 2C (Locomotor Activity): It is unexpected that the Stimx3 group does not show increased locomotion, given that the same stimulation protocol induces hyperactivity during the early phase of Stimx10. The authors should address this apparent discrepancy and provide a mechanistic or methodological explanation.

Response:

We appreciate the reviewer's insightful comment. The apparent discrepancy between the Stim×3 and Stim×10 groups can be explained by differences in temporal dynamics and behavioral context. In home cage monitoring, both groups exhibited a modest increase in locomotor activity beginning on the day after the first stimulation; however, this increase did not reach statistical significance in the Stim×10 group until day 7, as described in the original text (L176). This temporal pattern indicates that the lack of a significant increase in the Stim×3 group is consistent with the trajectory observed in the Stim×10 group. In contrast, in the open field test, both groups showed immediate hyperlocomotor activity after the first stimulation day (Figure 2B). These context-dependent differences likely reflect the contrast between familiar and novel environments. These points are now clarified in the revised text.

Change:

[Original: L176]

The distance traveled by mice in the Stim×10 group gradually increased after the initial stimulation and was significantly higher than that in the No Stim and Stim×3 groups ($P < 0.05$, Days 7–36; Fig. 2c). Mice in the Stim×3 group did not show any significant increase in locomotor activity in the home cage.

[Revised: L208]

The distance traveled by mice in the Stim×10 group gradually increased after the initial stimulation and was significantly higher than that in the No Stim and Stim×3 groups ($P < 0.05$, Days 7–36; Fig. 3c). In the home cage setting, unlike in the open field, locomotor activity did not increase immediately after stimulation, likely reflecting the differences between familiar and novel environments. Mice in the Stim×3 group did not show any significant increase in locomotor activity in the home cage.

Comment 7

Figure 2D (Behavioral Tests): The manuscript should clearly indicate in the Results section when the Tail Suspension Test (TST) and Forced Swim Test (FST) were conducted in relation to the stimulation timeline.

Response:

We agree with the reviewer's helpful comment and have clarified this point. The Results section has been revised to clearly indicate when the tail suspension test and forced swim test were conducted relative to the stimulation timeline.

Change:

[Original: L181]

Depression-related behavior was assessed with the tail suspension test (Fig. 2d). Mice in the Stim×10 group showed significantly less immobility compared to those in the No Stim (P = 0.0094) and Stim×3 groups (P = 0.0198). A similar trend was observed in the forced swim test (Fig. 2e), where Stim×10 mice showed reduced immobility compared to the No Stim group (P = 0.0304) and Stim×3 group (P = 0.0770).

[Revised: L218]

Depression-related behavior was assessed with the tail suspension test (Fig. 3d). Two weeks after the last stimulation, mice in the Stim×10 group showed significantly less immobility compared to those in the No Stim (P = 0.0094) and Stim×3 groups (P = 0.0198). A similar trend was observed in the forced swim test (Fig. 3e), where Stim×10 mice showed reduced immobility two weeks after the last stimulation compared to the No Stim group (P = 0.0304) and Stim×3 group (P = 0.0770).

Comment 8

Extended Figure 2: The data presented in Extended Figure 2 are central to understanding the

therapeutic potential of brain stimulation. These findings should be integrated into the main figures to enhance the impact and clarity of the results.

Response:

We appreciate the reviewer's suggestion. To enhance the impact and clarity of our findings, we have incorporated the data previously presented in Extended Data Figure 2 into the main figures and Results section, which is now presented as Figure 2 in the revised manuscript.

Change:

[Original: Extended Data Fig. 2] deleted

[Revised: new Fig. 2] added

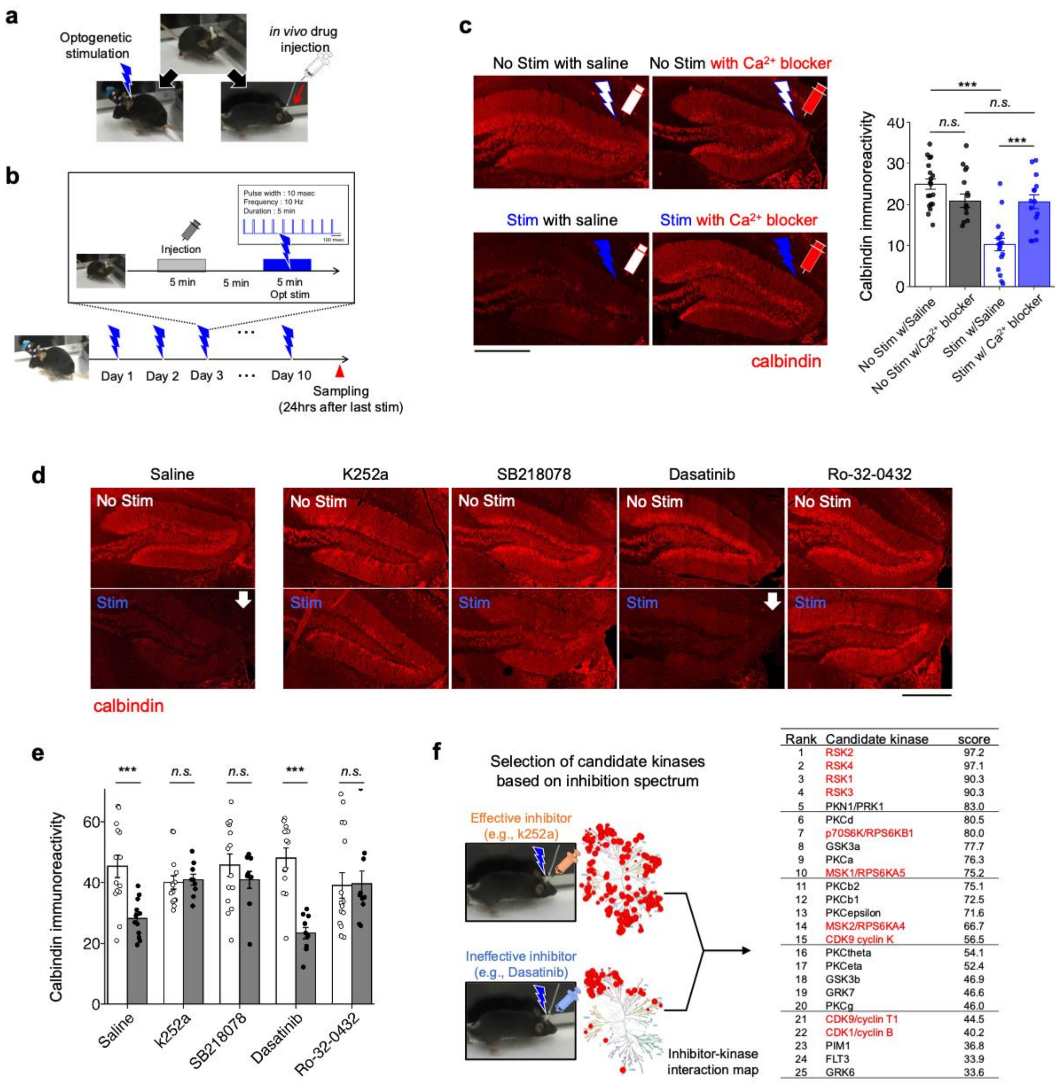

Rank	Candidate kinase	score
1	RSK2	97.2
2	RSK4	97.1
3	RSK1	90.3
4	RSK3	90.3
5	PKN1/PRK1	83.0
6	PKCd	80.5
7	p70S6K/RPS6KB1	80.0
8	GSK3a	77.7
9	PKCa	76.3
10	MSK1/RPS6KA5	75.2
11	PKCb2	75.1
12	PKCb1	72.5
13	PKCepsilon	71.6
14	MSK2/RPS6KA4	66.7
15	CDK9 cyclin K	56.5
16	PKCtheta	54.1
17	PKCeta	52.4
18	GSK3b	46.9
19	GRK7	46.6
20	PKCg	46.0
21	CDK9/cyclin T1	44.5
22	CDK1/cyclin B	40.2
23	PIM1	36.8
24	FLT3	33.9
25	GRK6	33.6

[Original: Extended Data Fig. 2, legend] deleted

[Revised: new Fig. 2, legend] added

Figure 2. Ca^{2+} influx and subsequent kinase activation are indispensable for inducing a cellular immature-like state.

a, Integrated optofluidic probe enabling localized drug injection and optogenetic stimulation through a single cannula implanted in the brain.

b, Experimental design. Intracerebral drug infusion into the DG was followed by optogenetic stimulation (5 min per day) for 10 consecutive days. Brains were sampled 24 hours after the final stimulation.

c, Effect of Ca^{2+} blockers on calbindin expression. The Ca^{2+} blockers included NBQX (AMPA-R antagonist), AP5 (NMDA-R antagonist), mibefradil (non-specific blocker of voltage-gated Ca^{2+} channels), and nimodipine (Ca_v1 blocker). Left: representative immunostaining of calbindin (red); scale bar, 500 μm. Right: bar graph showing calbindin immunoreactivity (mean ± s.e.m; 14–19 sections from 4 mice per group). One-way ANOVA; $F_{(3, 61)} = 19.17$, $P = 7.13 \times 10^{-9}$; Bonferroni correction was applied for multiple comparisons. $*P < 0.05$, $**P < 0.01$, $***P < 0.001$.

d, Effect of kinase inhibitors on calbindin expression. Four kinase inhibitors with distinct inhibition spectra (K252a, SB218078, Dasatinib, Ro-32-0432) and saline were injected before optogenetic stimulation. Representative calbindin (red) immunostaining images are shown; scale bar, 500 μm.

e, Quantification of calbindin immunoreactivity shown in **d.** Mean ± s.e.m. (9–16 sections from 4 mice per group). Two-way ANOVA; $F_{(4, 112)} = 5.60$, $P = 3.8 \times 10^{-4}$. Tukey's post hoc test was applied for multiple comparisons.

f, Identification of candidate kinases mediating REPOPS-induced dematuration. Left: schematic kinase dendrogram (modified from Anastassiadis et al., 2011[46]) showing inhibition profiles of the tested compounds (% inhibition at tested concentration); circle size indicates inhibition strength. Right: candidate kinases identified by comparing inhibition spectra of compounds that blocked (K252a, SB218078, and Ro-32-0432) or did not block (Dasatinib) REPOPS-induced effects. The score represents the difference in % inhibition for these compounds.

[Original: Supplementary Results 2] deleted

[Revised: L154] added

2. Ca^{2+} influx and subsequent kinase activation are indispensable for inducing a cellular immature-like state.

To dissect the signaling pathways underlying REPOPS-dependent dematuration, we developed an integrated optofluidic probe that enables simultaneous drug delivery and optogenetic stimulation within the same brain region through an implanted cannula (Fig. 2a). Using this device, we administered daily drug infusions followed by 5-min optogenetic stimulation for 10 consecutive days (Fig. 2b).

To test whether Ca^{2+} influx is required for dematuration, REPOPS was performed with a cocktail of Ca^{2+} blockers, including NBQX (AMPA-R antagonist), AP5 (NMDA-R antagonist), mibefradil (non-specific blocker of voltage-gated Ca^{2+} channels), and nimodipine (Ca_v1 blocker). In the saline-treated control group, REPOPS markedly reduced CB expression, whereas CB expression remained unchanged in Ca^{2+} blocker-treated mice (Fig. 2c). These results indicate that Ca^{2+} influx is crucial for neuronal dematuration.

Next, to explore kinases involved in dematuration, we conducted REPOPS with four kinase inhibitors with distinct inhibition spectra (Fig. 2d). K252a, SB218078, and Ro-32-0432 successfully inhibited REPOPS-induced CB reduction, whereas Dasatinib did not (Fig. 2d, 2e). Based on these results, we screened for candidate kinases potentially mediating dematuration. Using a comprehensive kinase-inhibitor activity dataset[46], we compared the inhibition spectra of the effective inhibitors with that of the ineffective one (Fig. 2f). Kinases more strongly inhibited by the effective inhibitors (K252a, SB218078, and Ro-32-0432) than by the ineffective inhibitor (Dasatinib) were shortlisted as candidates. This analysis identified 57 potential kinases, of which the top 25 are shown in Fig. 2f. These include RSK/MSK, which regulate chromatin remodeling through histone phosphorylation[47,48], and CDKs, key regulators of the cell cycle. Together, these findings suggest that Ca^{2+}-dependent signaling pathways and phosphorylation cascades play a pivotal role in REPOPS-induced dematuration.

Comment 9

Immunofluorescence quantification: The statistical analyses involving immunofluorescence data are not appropriately conducted. The reported "n" reflects the number of cells rather than biological replicates (i.e., number of animals). This is not statistically valid, as it inflates the sample size and underestimates variability. The authors should reanalyze these datasets using the number of mice as the unit of analysis.

Response:

We agree that treating individual cells as independent observations can inflate the sample size and underestimate variability. To address this issue, we reanalyzed all relevant

immunofluorescence datasets using a linear mixed-effects model (LMM) that included mouse ID as a random effect and the experimental group (e.g., stimulation condition) as a fixed effect (using the lmer function in R; Bates et al., *J. Stat. Softw.,* 2015). This approach estimates variability and statistical significance at the mouse level, thereby using the number of mice as the effective unit of analysis while accounting for correlations among cells within the same mouse. All relevant statistical descriptions and figure annotations have been updated accordingly in the revised manuscript.

Change:

[Original: Fig. 4, legend]

Figure 4. REPOPS confers G_2/M phase-like gene expression patterns and nuclear structure plasticity

b, Cyclin B immunostaining (red) in the DG. Beeswarm plot shows cyclin B immunoreactivity in individual neurons. Blue bars indicate the median values of 147 neurons (No Stim) and 202 (Stim×10+2wks) neurons from three biologically independent samples. ML, molecular layer; GCL, granule cell layer.

e, Lamin B1 immunostaining (red) in the DG. Enlarged views of boxed regions are shown at right. Arrows and arrowheads indicate neurons with partial or complete disruption of lamin B1 structure. Scale bars: 20 μm (left), 10 μm (right). Beeswarm plots show immunoreactivity and circularity of lamin B1 in individual neuron (n = 289 and 320 neurons from three independent samples). Blue bars, medians. Welch's *t*-tests: immunoreactivity, $t_{(589)} = 4.87$, $P = 1.45 \times 10^{-6}$; circularity, $t_{(547)} = 5.87$, $P = 7.38 \times 10^{-9}$.

f, Immunostaining of phospho-histone H3 (pH3) (red) in the DG. Scale bars: 200 μm (left), 10 μm (right). Beeswarm plot shows pH3 signal intensity in individual neurons (n = 415 and 459 neurons from three independent samples). Welch's *t*-test: $t_{(858)} = 14.1$, $P < 2.2 \times 10^{-16}$.

g, DNA counterstaining with Hoechst (blue and white pseudo-color) and cyclin B immunostaining (red) in the DG. Enlarged views of boxed areas are shown on the right. Red arrowheads indicate chromocenters–an aggregation of the heterochromatin domain visible by Hoechst staining. White arrowheads/arrows: neurons with large/small chromocenters and high/low cyclin B expression, respectively. Scale bars: 100 μm (left), 10 μm (right). Beeswarm plot shows the chromocenter ratio (%) per neurons (n = 147 and 202 neurons from three independent samples). Welch's *t*-test: $t_{(306)} = 5.46$, $P = 9.38 \times 10^{-8}$.

[Revised: Fig. 5, legend]

Figure 5. REPOPS confers G_2/M phase-like gene expression patterns and nuclear structure plasticity.

b, Cyclin B immunostaining (red) in the DG. Beeswarm plot shows cyclin B immunoreactivity in individual neurons. Blue bars indicate the median values of 147 neurons (No Stim) and 202 (Stim×10+2wks) neurons from three biologically independent samples. ML, molecular layer; GCL, granule cell layer. Linear mixed-effects model (LMM) treating mouse as a random effect and experimental group as a fixed effect; $t_{(4.20)} = 10.9$, $P = 3.1 \times 10^{-4}$.

e, Lamin B1 immunostaining (red) in the DG. Enlarged views of boxed regions are shown at right. Arrows and arrowheads indicate neurons with partial or complete disruption of lamin B1 structure. Scale bars: 20 μm (left), 10 μm (right). Beeswarm plots show immunoreactivity and circularity of lamin B1 in individual neurons (n = 289 and 320 neurons from three independent samples). Blue bars, medians. LMM: Lamin B1 immunoreactivity, $t_{(4.24)} = -3.84$, $P = 0.016$; Circularity, $t_{(4.07)} = -2.79$, $P = 0.048$.

f, Immunostaining of phospho-histone H3 (pH3; red) in the DG. Scale bars: 200 μm (left), 10 μm (right). Beeswarm plot shows pH3 signal intensity in individual neurons (n = 415 and 459 neurons from three biologically independent samples). LMM: $t_{(6.00)} = 3.40$, $P = 0.015$.

g, DNA counterstaining with Hoechst (blue and white pseudo-color) and cyclin B immunostaining (red) in the DG. Enlarged views of boxed areas are shown on the right. Red arrowheads indicate chromocenters–an aggregation of the heterochromatin domain visible by Hoechst staining. White arrowheads/arrows: neurons with large/small chromocenters and high/low cyclin B expression, respectively. Scale bars: 100 μm (left), 10 μm (right). Beeswarm plot shows the chromocenter ratio (%) per neuron (n = 147 and 202 neurons from three independent samples). LMM: $t_{(2.84)} = 4.65$, $P = 0.021$.

[Original: Fig. 5, legend]

Figure 5. Cyclin B deletion reverses multiple REPOPS-induced phenotypes

c, Lamin B1 immunostaining in the DG (converted to red for visibility; originally stained with Alexa647). Upper and lower panels show control (Scramble-KO) and *Ccnb1*-*Ccnb2*-KO (CyclinB-KO) mice, respectively. Scale bars: 20 μm (left), 10 μm (right). Beeswarm plot shows lamin B1 immunoreactivity. Red bars, median. One-way ANOVA, $F_{(3, 679)} = 60.19$, $P < 2.0 \times 10^{-16}$; Bonferroni corrected, ***$P < 0.001$. n = 245, 301, 271, and 246 neurons from three independent samples per group.

d, pH3 immunostaining in the DG. One-way ANOVA, $F_{(3, 679)} = 325.7$, $P < 2.0 \times 10^{-16}$; ***$P < 0.001$; 199, 158, 143, and 183 neurons from five independent samples per group. Scale bar, 20 μm.

e, Chromocenter visualized by Hoechst staining (white, pseudo-color). One-way ANOVA, $F_{(3, 679)}$ = 39.1, $P < 2.0 \times 10^{-16}$; ***$P < 0.001$; 199, 158, 143, and 183 neurons from five independent samples per group. Scale bar, 10 μm.

f, Calbindin staining in the DG. One-way ANOVA, $F_{(3, 725)}$ = 104.3, $P < 2.0 \times 10^{-16}$; ***$P < 0.001$; 217, 157, 162, and 198 neurons from five biologically independent samples per group. Scale bar, 20 μm.

[Revised: Fig. 6, legend]

Figure 6. Cyclin B deletion reverses multiple REPOPS-induced phenotypes.

c, Lamin B1 immunostaining in the DG (converted to red for visibility; originally stained with Alexa647). Upper and lower panels show control (Scramble-KO) and *Ccnb1*-*Ccnb2*-KO (CyclinB-KO) mice, respectively. Scale bars: 20 μm (left), 10 μm (right). Beeswarm plot shows lamin B1 immunoreactivity. Red bars, median. LMM treating mouse as a random effect and experimental group as a fixed effect; $F_{(3, 12.1)}$ = 3.82, $P = 0.039$; post hoc pairwise comparisons: *$P < 0.05$, **$P < 0.01$, ***$P < 0.001$.

d, pH3 immunostaining in the DG. One-way ANOVA, $F_{(3, 679)}$ = 325.7, $P < 2.0 \times 10^{-16}$; ***$P <$ 0.001; 199, 158, 143, and 183 neurons from five independent samples per group. Scale bar, 20 μm. LMM: $F_{(3, 8.0)}$ = 177.6, $P = 1.6 \times 10^{-7}$; post hoc pairwise comparisons as above.

e, Chromocenter visualized by Hoechst staining (white, pseudo-color). Scale bar, 10 μm. LMM: $F_{(3, 7.84)}$ = 4.79, $P = 0.034$; post hoc pairwise comparisons as above.

f, Calbindin staining in the DG. Scale bar, 20 μm. LMM: $F_{(3, 7.44)}$ = 31.7, $P = 1.33 \times 10^{-4}$; post hoc pairwise comparisons as above.

[Original: Fig. 6, legend]

Figure 6. Cyclin B/Cdk1 activity mediates long-term ΔFosB expression and pseudo-immaturity of neurons

c, ΔFosB immunostaining in the DG of Scram-KO and CyclinB-KO mice (same mice as in Fig. 5). Bottom panels show enlarged views of the white boxes. Grayscale indicates Hoechst staining. Scale bar, 20 μm. Beeswarm showing ΔFosB immunoreactivity (n = 129–165 cells per group). Black bars indicate the median. One-way ANOVA: $F_{(5, 859)}$ = 270.94, $P < 2.0 \times 10^{-16}$; post hoc comparisons with Tukey's test.

f, Beeswarm plots showing ΔFosB, calbindin, and chromocenter ratio. n = 114–156 cells per group. Black bars indicate the median. One-way ANOVA: ΔFosB: $F_{(4, 649)}$ = 405.45, $P < 2.0 \times 10^{-16}$; Calbindin: $F_{(4, 655)}$ = 117.65, $P < 2.0 \times 10^{-16}$; Chromocenter ratio: $F_{(4, 655)}$ = 14.63, $P < 2.0 \times 10^{-16}$. Multiple comparisons were corrected using Tukey's test.

[Revised: Fig. 7, legend]

Figure 7. Cyclin B/Cdk1 activity mediates long-term ΔFosB expression and pseudo-immaturity of neurons.

c, ΔFosB immunostaining in the DG of Scram-KO and CyclinB-KO mice (same mice as in Fig. 6). Bottom panels show enlarged views of the white boxes. Grayscale indicates Hoechst staining. Scale bar, 20 µm. Beeswarm showing ΔFosB immunoreactivity (n = 129–165 cells per group). Black bars indicate the median. LMM: $F_{(5, 18.1)} = 66.8$, $P = 5.1 \times 10^{-11}$; post hoc pairwise comparisons: *$P < 0.05$, **$P < 0.01$, ***$P < 0.001$.

f, Beeswarm plots showing ΔFosB, calbindin, and chromocenter ratio. n = 114–156 cells per group. Black bars indicate the median. LMM: ΔFosB: $F_{(4, 15.6)} = 190.8$, $P = 5.1 \times 10^{-13}$; Calbindin: $F_{(4, 14.3)} = 90.2$, $P = 5.7 \times 10^{-10}$; Chromocenter ratio: $F_{(4, 13.5)} = 14.0$, $P = 1.0 \times 10^{-4}$; post hoc pairwise comparisons as above.

[Original: Extended Data Fig. 9, legend]

Extended Data Figure 9. ΔFosB reduces calbindin expression in DG neurons.

d, Immunostaining of calbindin and ΔFosB in the DG. Beeswarm plot shows calbindin immunoreactivity in individual neurons (n = 142-182 neurons per group). Black bars indicate the median. One-way ANOVA: $F_{(3, 645)} = 100.63$, $P < 2.0 \times 10^{-16}$; post hoc comparisons using Tukey's test, ***$P < 0.001$.

[Revised: Extended Data Fig. 14, legend]

Extended Data Figure 14. ΔFosB reduces calbindin expression in DG neurons.

d, Immunostaining of calbindin and ΔFosB in the DG. Beeswarm plot shows calbindin immunoreactivity in individual neurons (n = 142-182 neurons per group). Black bars indicate the median. LMM: $F_{(3, 7.81)} = 48.8$, $P = 2.7 \times 10^{-5}$; post hoc pairwise comparisons: *$P < 0.05$, **$P < 0.01$, ***$P < 0.001$.

[Original: Extended Data Fig. 6, legend]

Extended Data Figure 6. Mitosis-like epigenetic modifications are associated with heterochromatin enlargement

a, STED microscopy images showing phospho-histone H3 (pH3; red) and histone H3 lysine 9 tri-methylation (H3K9me3; green), a marker of highly condensed heterochromatin domains[3]. Scale bar, 5 µm. The violin-box plot shows the size distribution of H3K9me3-positive regions. Center lines represent medians; box boundaries show upper and lower quartiles; whiskers represent maxima and minima; and diamonds denote mean values. 578 and 514 neurons from

four biologically independent samples in the No Stim and Stim×10+2wks groups, respectively. Welch's *t*-test, $t_{(1018)} = 5.32$, $P = 1.25 \times 10^{-7}$.

[Revised: Extended Data Fig. 10, legend]

Extended Data Figure 10. Mitosis-like epigenetic modifications are associated with heterochromatin enlargement.

b, STED microscopy images showing phospho-histone H3 (pH3; red) and histone H3 lysine 9 tri-methylation (H3K9me3; green), a marker of highly condensed heterochromatin domains[2]. Scale bar, 5 μm. The violin-box plot shows the size distribution of H3K9me3-positive regions. Center lines represent medians; box boundaries show upper and lower quartiles; whiskers represent maxima and minima; and diamonds denote mean values. 578 and 514 neurons from four biologically independent samples in the No Stim and Stim×10+2wks groups, respectively. LMM: $t_{(5.70)} = 3.17$, $P = 0.021$.

[Original: L1180]

Statistical Analyses

Mice of the same age and sex were raised under identical conditions and were randomly allocated to different experimental groups. Blinding was not applied in this study because the animals needed to be controlled by the stimulation conditions. The sample sizes were determined based on those reported in similar publications[30,31]. Welch's t-test was used to compare the averages. An ANOVA was performed when more than two groups were compared, followed by the Bonferroni post-hoc method. For correlation analysis, we used Pearson correlation analysis for normally distributed data. When pooling the data of neurons from multiple mice per group, a Linear Mixed-Effects Regression model was applied to account for individual differences per mouse. All results of the statistical analyses are summarized in Supplementary Table 2.

[Revised: L1326]

Statistical Analyses

Mice of the same age and sex were raised under identical conditions and were randomly allocated to different experimental groups. Blinding was not applied in this study because the animals needed to be controlled by the stimulation conditions. The sample sizes were determined based on those reported in similar publications[27,28]. Welch's *t*-test was used to compare group means. One-way or two-way ANOVA was applied for comparisons involving more than two groups, followed by Bonferroni's post hoc tests. When pooling the data of neurons from multiple mice per group, a linear mixed-effects model (LMM) was applied, treating

mouse ID as a random effect to account for within-animal correlations. All results of the statistical analyses of the behavioral tests are summarized in Supplementary Table 4.

Comment 10

Discussion: References 57 and 58 do not substantiate the claims made in the corresponding sentence. These should be removed or replaced with more appropriate citations.

Response:

We appreciate the reviewer's careful observation. We have removed References 57 and 58 (Herrup et al., 2007; Frost et al., 2016) and replaced them with a more relevant citation (Frost, Trends Neurosci., 2023), which directly substantiates the statement.

Change:

[Original: References] removed

57, Herrup et al

58, Frost et al

[Revised: Reference] added

67, Frost et al., *TINS*, 2023

Comment 11

Several studies have reported that neuronal dematuration can have detrimental effects on brain function and neuronal activity. The authors should address this apparent discrepancy by discussing why, in their model, dematuration is associated with seemingly beneficial behavioral outcomes. In particular, they should consider the timing of behavioral assessments, as this could significantly influence the observed effects (see earlier comment regarding the timeline).

Response:

We appreciate the reviewer's insightful comment. We agree that it is important to clarify the factors determining whether neuronal dematuration leads to beneficial or detrimental outcomes.

We consider that the intensity and repetition of neural hyperactivity are key determinants. Therapeutic paradigms such as ECT or REPOPS involve brief, intermittent, and well-controlled stimulation (Espinoza et al., N. Engl. J. Med., 2022; Salik et al., StatPearls, 2025), whereas pathological conditions such as epilepsy and Alzheimer's disease are characterized by uncontrolled and chronic hyperexcitability, resulting in irreversible network dysfunction (Palop et al., Nat. Rev. Neurosci., 2016). The timing of behavioral assessments is also critical, as the

reviewer noted. In our model, behavioral outcomes change in a time-dependent manner (Fig. 2a, b). Similarly, symptoms in epilepsy and Alzheimer's disease fluctuate across disease stages (Devinsky et al., Nat. Rev. Dis. Primers, 2018).

These distinctions—between regulated versus pathological activation and between acute versus chronic phases—help reconcile the seemingly opposite effects of dematuration. These points have been clarified in the revised Discussion.

Change:

[Original: L452]

A key insight from the present work is that the immature cellular state appears to be bistable; that is, it can serve as a reversible therapeutic intermediate, as observed in ECT, or consolidate into an irreversible pathological lesion, as seen in disease models.

[Revised: L494]

A key insight from the present work is that the immature cellular state appears to be bistable; that is, it can serve as a reversible therapeutic intermediate, as observed in ECT, or consolidate into an irreversible pathological lesion, as seen in disease models. This bistable nature likely depends on the intensity and repetition of neural hyperactivity. Therapeutic paradigms such as ECT or REPOPS involve brief, intermittent, and well-regulated stimulation (Espinoza et al., N. Engl. J. Med., 2022; Salik et al., StatPearls, 2025)[29,109]. In contrast, pathological conditions such as epilepsy and Alzheimer's disease are characterized by uncontrolled and chronic hyperexcitability (Palop et al., Nat. Rev. Neurosci., 2016)[110]. Timing is also critical: whether the system is in a stable recovery phase after transient hyperactivity or remains in a state of recurrent excitation profoundly influences the outcome. In our paradigm, behavioral testing conducted approximately two weeks after the final stimulation session likely reflects a stabilized phase of circuit reorganization, whereas patients are often in a state of persistent hyperexcitability, in which neuronal homeostasis is disrupted (Devinsky et al., Nat. Rev. Dis. Primers, 2018)[111].

Comment 12

While the authors specifically targeted neurons in their manipulation, the RNA-seq analysis does not distinguish between neuronal and glial differentially expressed genes (DEGs). Given the emerging role of glial cells in brain plasticity and rejuvenation, the inability to isolate neuron-specific transcriptomic changes represents a limitation. The authors should acknowledge this and briefly discuss the potential contribution of glial responses to the observed effects.

Editorial Note: Supplementary Fig. 15 in this Peer Review File is reproduced with permission from BioRender, Created in BioRender. Murano, T. (2026) https://BioRender.com/p6ed2mm

Response:

As the reviewer pointed out, our bulk RNA-seq analysis does not distinguish between neuronal and glial cells, making it unclear which cell types primarily contribute to the observed transcriptomic changes. To address this limitation, we performed a cell-type enrichment analysis using a publicly available single-cell RNA-seq reference dataset (Hochgerner et al., Nat. Neurosci., 2018). This analysis revealed that granule cell- and neuroblast-specific genes were predominantly downregulated and upregulated by REPOPS, respectively, whereas glial cell-specific genes were also altered. These results suggest that the majority of transcriptomic changes in our bulk RNA-seq data likely originate from neurons and neuroblasts, while potential glial responses may also contribute. These results have been added as Extended Data Fig. 15 and described in the Supplementary Results 2.

Change:

[Original: ---]

[Revised: Extended Data Fig. 15a, b, c] added

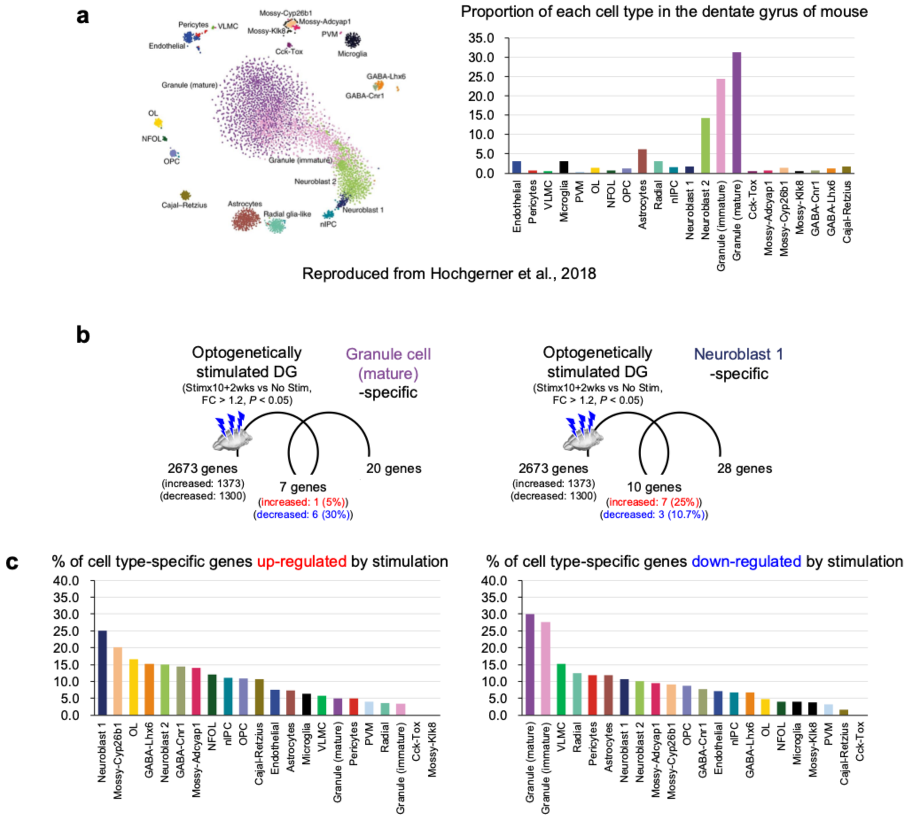

[Original: ---]

[Revised: Extended Data Fig. 15, legend] added

Extended Data Figure 15. REPOPS induces transcriptomic changes across multiple cell types, with predominant effects on granule cells.

a, Left, visualization of single-cell clusters in the mouse dentate gyrus, color-coded by cell-type cluster annotation (*reproduced from* Hochgerner *et al., Nat. Neurosci.,* 2018[3]). Right, proportion of each cell type relative to the total cell population.

b, Venn diagram showing the overlap between granule cell (mature)– or neuroblast-specific genes and genes significantly altered in the Stim×10+2wks group.

c, Bar plot illustrating the proportion of genes significantly up- or down-regulated in the Stim×10+2wks group within each cell type–specific gene set. Cell type classifications on the x-axis are based on Hochgerner *et al.,* 2018.

d, Immunostaining for NeuN (excitatory neurons), PV (interneurons), GFAP (astrocytes), and Iba1 (microglia) in the DG. Scale bar, 500 μm. The bar graph shows the density of marker-positive cells.

e, mRNA expression of representative cell-type marker genes. Data are shown as mean ± s.e.m. (n = 6 mice per group). *P* values were calculated using Welch's *t*-test.

[Original: ---]

[Revised: Supplementary Result 2] added

Supplementary Result 2. REPOPS induces transcriptomic changes across multiple cell types, with predominant effects on granule cells (related to Extended Data Fig. 15).

Our bulk RNA-seq analysis (Fig. 1c–f) captured gene expression changes not only in granule cells (GCs) but also in other cell types, including mossy cells, interneurons, and glial cells. To estimate the relative contribution of each cell type, we performed a cell-type enrichment analysis using the dentate gyrus single-cell RNA-seq dataset reported by Hochgerner et al.[3]. Extended Data Fig. 15a shows the cell-type annotations and their relative proportions within the DG, identical to those in the original reference. Marker genes for each cell type were defined as those expressed >10-fold higher in that specific cell type than the mean across all others. We compared these marker genes with the differentially expressed genes identified in the Stim×10+2wks group and calculated the proportion of marker genes that were significantly up- or down-regulated for each cell type (Extended Data Fig. 15b). Genes associated with immature excitatory neurons (Neuroblast1 cluster) were most prominently upregulated, whereas those associated with mature granule cells were most downregulated (Extended Data Fig. 15c). Cell-type-specific genes for other populations (e.g., glial and mossy cells) also

showed moderate alterations, indicating that REPOPS broadly influences multiple cellular populations within the DG.

To further validate these findings, we performed immunostaining for NeuN, parvalbumin (PV), GFAP, and Iba1 and quantified the density of marker-positive cells (Extended Data Fig. 15d). None of these markers showed significant differences in the number of positive cells after REPOPS, indicating that the bulk-level transcriptomic changes are unlikely to be explained simply by large shifts in cell numbers. We also analyzed the mRNA expression of representative cell-type marker genes (Prox1 and Calb1 for granule cells, Calb2 for mossy cells, Pvalb and Sst for interneurons, Gfap for astrocytes, and Aif1 (Iba1) for microglia). REPOPS for 10 days significantly reduced the expression of Prox1, Calb1, and Iba1, increased Gfap expression, and did not change the expression of Calb2, Pvalb, or Sst (Extended Data Fig. 15e). Given that granule cells are overwhelmingly predominant within the DG cell composition (Extended Data Fig. 15a, d), these results support the conclusion that, although REPOPS affects multiple cell types, the majority of transcriptomic alterations observed in our bulk RNA-seq data are primarily driven by granule cells.

Reviewer #2

Comment 1

It's arguable that the REPOPS model, which delivers local stimulation to DG granule cells is a valid model for ECT, which induces generalized seizures. It would be more appropriate to frame this as a neurostimulation protocol more generally.

Response:

We appreciate the reviewer's important comment. We agree that REPOPS differs fundamentally from ECT, which induces generalized seizures. REPOPS was designed to experimentally examine how repeated neuronal activation alters the cellular properties and functional state of neural circuits, rather than to replicate ECT per se. Accordingly, we have revised the relevant descriptions in the Introduction and Discussion, replacing the phrase "model of ECT" with more precise and neutral terms such as "ECT-inspired" or "repeated neuronal activation."

Change:

[Original: L43]

These findings demonstrate that ECT-like brain stimulation triggers plasticity of the cellular state, revealing a form of stimulus-regulated nuclear reprogramming with potential clinical utility.

[Revised: L43]

These findings demonstrate that chronically repeated brain stimulation triggers plasticity of the cellular state, revealing a form of stimulus-regulated nuclear reprogramming with potential clinical utility.

[Original: L88]

We applied an ECT-like optogenetic stimulation to granule cells of the hippocampal dentate gyrus, a key brain area for ECT, to produce clinically relevant behavioral effects.

[Revised: L89]

We applied an optogenetic stimulation to granule cells of the hippocampal dentate gyrus under a protocol inspired by ECT, to produce clinically relevant behavioral effects.

[Original: L94]

These findings reveal a unique immature cellular state induced by ECT-like neuronal activation, which has long-term stability, and a mechanism of activity-dependent nuclear reprogramming, offering mechanistic insight into ECT effects and related applications.

[Revised: L95]

These findings reveal a unique immature cellular state induced by chronically repeated neuronal activation, which has long-term stability, and a mechanism of activity-dependent nuclear reprogramming, offering mechanistic insight into ECT effects and related applications.

[Original: L162]

To assess whether ECT-like neuronal activation could alter anti-depressive behavior, we first performed an open field (OF) test (Fig. 2a, 2b).

[Revised: L194]

To assess whether repeated neuronal activation could alter anti-depressive behavior, we first performed an open field (OF) test (Fig. 3a, 3b).

[Original: L348]

We developed an ECT-like optogenetic stimulation, REPetitive OPtogenetic Stimulation (REPOPS), which reproducibly induces “dematuration” as indexed by global changes in the cellular transcriptome and 3D genome structure.

[Revised: L391]

We developed an optogenetic stimulation paradigm, REPetitive OPtogenetic Stimulation (REPOPS), which reproducibly induces “dematuration” as indexed by global changes in the cellular transcriptome and 3D genome structure.

[Original: L407]

The current results demonstrate that cellular dematuration triggered by an ECT-like stimulus can produce positive behavioral phenotypes, including decreased depression-like symptoms and increased motor activity, similar to the clinical ECT effects.

[Revised: L448]

The current results demonstrate that cellular dematuration triggered by chronically repeated stimulation can produce positive behavioral phenotypes, including decreased depression-like symptoms and increased motor activity, similar to the clinical ECT effects.

[Original: L414]

Activation of DG neurons is also known to suppress depression-like behaviors[86,87], therefore, increased speed-related activities of DG neurons may explain the link between ECT-like stimulation and the reduction in depression-like behavior observed in the tail suspension and forced swim tests (Fig. 2d, 2e).

[Revised: L456]

Activation of DG neurons is also known to suppress depression-like behaviors[91,92], therefore, increased speed-related activities of DG neurons may explain the link between chronically repeated stimulation and the reduction in depression-like behavior observed in the tail suspension and forced swim tests (Fig. 3d, 3e).

[Original: L429]

Thus, ECT-like REPOPS alters neural coding, leading to both decreased depressive-like behaviors and memory deficits. Further research will address the ECT stimulus conditions that do not impair memory and learning, but preserve beneficial clinical therapeutic effects.

[Revised: L471]

Thus, REPOPS alters neural coding, leading to both decreased depressive-like behaviors and memory deficits. Further research will address the stimulus conditions that preserve beneficial clinical therapeutic efficacy without impairing memory or learning.

Comment 2

The behavioral analyses (Figure 2) used to assess antidepressant-like effects (FST, TST) unfortunately are confounded by the hyperlocomotive phenotype induced by the stimulation. Additional assays that are less reliant on locomotor outputs such as sucrose preference or novelty suppressed feeding could be considered.

Response:

We thank the reviewer for raising this important point. As suggested, we performed the novelty-suppressed feeding test (NSFT) and the sucrose preference test (SPT), which are well-established assays for antidepressant-like effects that are less dependent on locomotor activity. In the NSFT, REPOPS for 10 days significantly shortened the latency to feed, indicating reduced anxiety-related behavior. In the SPT, no significant differences were observed between groups. These results are now included in Extended Data Fig. 5 and described in the Supplementary Result 1.

Change:

[Original: ---]

[Revised: Extended Data Fig. 5c, 5d] added

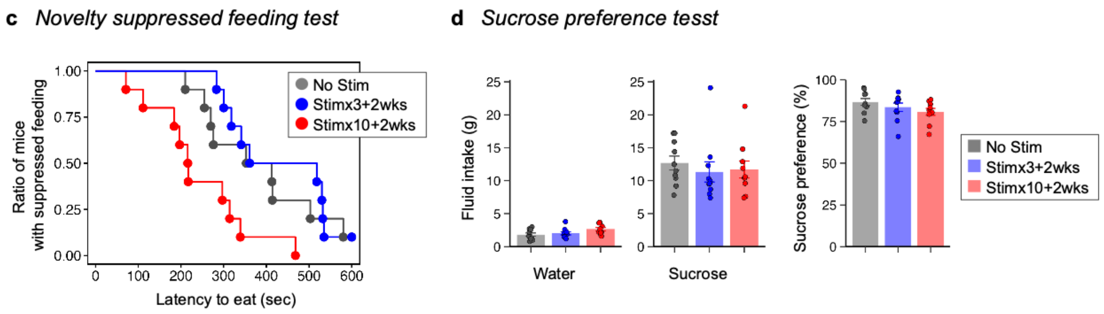

[Original: ---]

[Revised: Extended Data Fig. 5c, 5d, legend] added

Extended Data Figure 5. REPOPS for 10 days induces anti-depressive behavioral changes in mice.

c, Novelty-suppressed feeding test. Latency to feed per mouse shown as Kaplan–Meier survival curves depicting the probability of not eating over time. Log-rank Mantel–Cox analysis: Stim×10 vs. No Stim, χ^2 = 5.09, P = 0.024; Stim×10 vs. Stim×3, χ^2 = 10.20, P = 0.0014; Stim×3 vs. No Stim, χ^2 = 0.49, P = 0.49. ANCOVA controlling for locomotor activity revealed a significant main effect of stimulation group on feeding latency ($F_{(2, 26)}$ = 6.75, P = 0.0045), whereas total distance during the test ($F_{(1, 25)}$ = 0.72, P = 0.40) and sex ($F_{(1, 25)}$ = 3.36, P = 0.079) were not significant.

d, Sucrose preference test. Water and sucrose solution intake (g) and sucrose preference (%). Data are presented as mean ± s.e.m.

[Original: Supplementary Results 1]

[Revised: Supplementary Results 1] added

Supplementary Result 1. Behavioral analysis of optogenetically stimulated mice (related to Extended Data Figs. 5 and 6)

To further examine whether REPOPS alleviates depression-related behavior, we performed the novelty-suppressed feeding test, a well-established assay for antidepressant-like effects

that is less dependent on locomotor activity[5]. In this paradigm, food-deprived mice are placed in a brightly lit open field with a food pellet in the center, and the latency to begin feeding is recorded. Feeding latency differed significantly among the three groups (log-rank Mantel–Cox test: χ^2 = 11.86, P = 0.0027). Post hoc pairwise comparisons revealed that feeding latency was significantly shorter in the Stim×10 group than in both the No Stim (χ^2 = 5.09, P = 0.024) and Stim×3 groups (χ^2 = 10.20, P = 0.0014), whereas no significant difference was detected between the No Stim and Stim×3 groups (χ^2 = 0.25, P = 0.62; Extended Data Fig. 5c). To determine whether this effect was attributable to differences in general activity levels, ANCOVA controlling for locomotor activity confirmed that the main effect of stimulation group on feeding latency remained significant ($F_{(2, 18)}$ = 6.93, P = 0.0059). When sex and the interaction term were further entered into the model, there were no significant effects of locomotor activity ($F_{(1, 18)}$ = 0.74, P = 0.40), sex ($F_{(1, 18)}$ = 3.44, P = 0.080), or their interaction ($F_{(2, 18)}$ = 0.74, P = 0.49). These results demonstrate that 10-day, but not 3-day, REPOPS reduces anxiety-related behavior independently of general locomotor activity. We also performed the sucrose preference test. No statistically significant differences were observed among groups in sucrose or water intake or in the percentage of sucrose preference (Extended Data Fig. 5d). These findings suggest that REPOPS does not alter anhedonia-like behavior, although the high baseline sucrose preference in wild-type mice may have limited the sensitivity of this test.

[Original: ---]
[Revised: L1046]

Novelty-suppressed feeding test (NSFT)

Mice were individually housed in plastic cages (250 × 182 × 139 mm) with fresh paper-chip bedding. After 24-h food deprivation with ad libitum access to water, each mouse was placed in a brightly lit (400 lux) novel open field (square acrylic arena, 40 × 40 cm) containing a single familiar chow pellet at the center. Latency to feed was recorded as the time from placement in the arena until the mouse grasped and bit the pellet with its forepaws while sitting on its haunches. A cutoff time of 600 s was applied to non-eaters. Immediately after the test, mice were returned to their home cage, and percent body-weight loss during deprivation was calculated to control for appetite and motivational differences. The procedure followed established protocols[125].

Sucrose preference test (SPT)

Mice were individually housed in plastic cages (250 × 182 × 139 mm) and given two bottles of filtered tap water for acclimation. On the following day, one bottle was replaced with 1% (w/v)

sucrose solution while the other contained water. Fluid intake was measured by weighing the bottles at approximately 24-h intervals for 2 consecutive days, with left–right positions alternated daily to prevent side bias. Sucrose preference (%) was calculated as 100 × [mean sucrose intake / (mean sucrose + mean water intake)] across the 2-day testing period.

Comment 3

In establishing the model (Figure 1), it would be helpful to include a control virus to demonstrate whether changes in cellular maturity are mediated through specific activation of GC's vs nonspecific effects of optical stim of the region.

Response:

We thank the reviewer for this comment. In our study, ChR2 was selectively expressed in granule cells through the crossing of POMC-Cre mice with a Cre-dependent ChR2 reporter line (POMC-Cre × ChR2), as described in the Materials and Methods section.

The issue raised by the reviewer has already been addressed in Extended Data Figure 1d (groups 4 vs. 5) of the original text. We confirmed that optogenetic stimulation in mice lacking ChR2 expression (POMC-Cre × WT) did not cause a significant decrease in calbindin expression. This result indicates that the REPOPS-induced reduction in calbindin was specifically mediated by neuronal activation, rather than by nonspecific effects of light exposure. We have clarified this point in the legend of Extended Data Fig. 1c.

Change:

[Original: Extended Data Fig. 1d]

[Revised: Extended Data Fig. 1c]

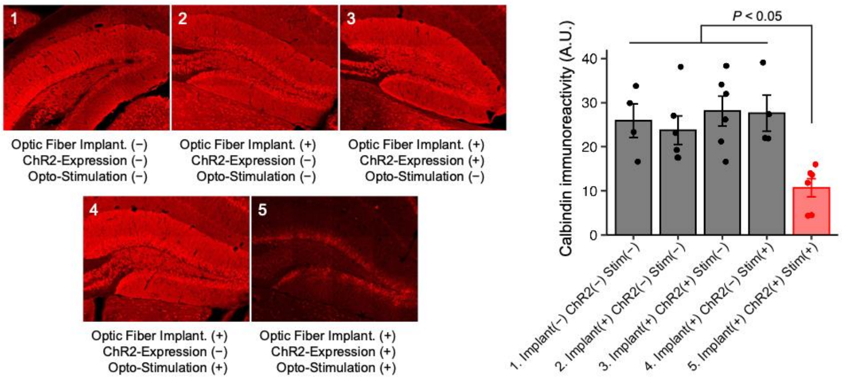

[Original: Extended Data Fig. 1d, legend]

d, Calbindin staining in the DG under five experimental conditions (1−5), representing combinations of optic fiber implantation, ChR2 expression in GCs, and optogenetic stimulation. The bar graph shows calbindin immunoreactivity. Mean ± s.e.m. (4–6 sections from 4 mice/group). One-way ANOVA; $F_{(4,26)}$ = 0.3244, P = 0.858; Tukey's HSD post hoc test was applied for multiple comparisons.

[Revised: Extended Data Fig. 1c, legend]

c, Calbindin staining in the DG under five experimental conditions (1−5), representing combinations of optic fiber implantation, ChR2 expression in GCs, and optogenetic stimulation. The bar graph shows calbindin immunoreactivity. Mean ± s.e.m. (4–6 sections from 4 mice/group). One-way ANOVA; $F_{(4,26)}$ = 0.3244, P = 0.858; Tukey's HSD post hoc test was applied for multiple comparisons.

Calbindin expression was not significantly affected by optic fiber implantation, ChR2 expression, or light stimulation itself, indicating that the REPOPS-induced reduction in calbindin was not attributable to surgical inflammation or heat generated by light exposure.

Reviewer #3

Major Comment 1

Confounding locomotor effects in behavioral assays: Since REPOS induces speed-activity encoding in DG and significantly enhances locomotor activity, it is difficult to tease-apart whether the observed effects on FST/TST are simply an outcome of increased locomotion or an actual anti-depressant behavior. I think the reduction in immobility in FST and TST after REPOS likely reflects increased locomotor activity, rather than a true antidepressant-like effect. Standard practice is to control for locomotor activity when interpreting these assays. Therefore, the conclusion that REPOS reduces depression-like behavior is speculative and potentially misleading. Moreover, the observed impairment in social interaction contradicts the interpretation of reduced depression-like behavior. The authors could reanalyze immobility after adjusting for locomotor activity, or test whether FST/TST behavior is rescued in mice with Cyclin B knockdown, as they did for locomotor activity. If not possible, the authors should tone down their claim of 'REPOS inducing long-term anti-depressant effects' and explicitly acknowledge this as a strong limitation of their study.

Response:

We thank the reviewer for this important comment. This concern is consistent with Comment 2 from Reviewer 2. We agree that increased locomotor activity could potentially influence depression-like behaviors. To address this, we conducted the novelty-suppressed feeding test (NSFT)**,** a widely accepted assay for antidepressant-like effects that is less dependent on locomotor activity. In the Stim×10+2wks group, the latency to feed was significantly shorter than that in the No Stim group, whereas total distance traveled did not differ among groups. ANCOVA controlling for locomotor activity revealed a significant main effect of stimulation group on feeding latency ($F_{(2,26)}$ = 6.12, P = 0.0066), whereas the covariate, total distance traveled, had no significant effect ($F_{(1,26)}$ = 0.33, P = 0.57). These results indicate that REPOPS exerts antidepressant-like effects independently of general locomotor activity**.**

We also appreciate the reviewer's comment regarding the social interaction test. Indeed, the reduced social interaction observed in REPOPS mice does not appear to represent classical antidepressant-like effects. To further examine this issue, we reanalyzed locomotor activity during the social interaction test. There was a trend toward increased activity in the stimulated groups, although total distance traveled did not differ significantly among groups (P = 0.63). Social interaction is known to be strongly influenced by novelty-seeking and locomotor drive (Kim et al., *Exp. Neurobiol.,* 2019); therefore, the reduced social preference observed in the Stim×10 group

may reflect enhanced exploratory behavior rather than impaired sociability. Nevertheless, we cannot exclude the possibility that REPOPS genuinely reduces sociability, indicating that its effects are not uniformly antidepressant-like. We have added these analyses as Extended Data Fig. 3c and described both possible interpretations in the Supplementary Results section.

Change:

[Original: ---]

[Revised: Extended Data Fig. 5c,d] added

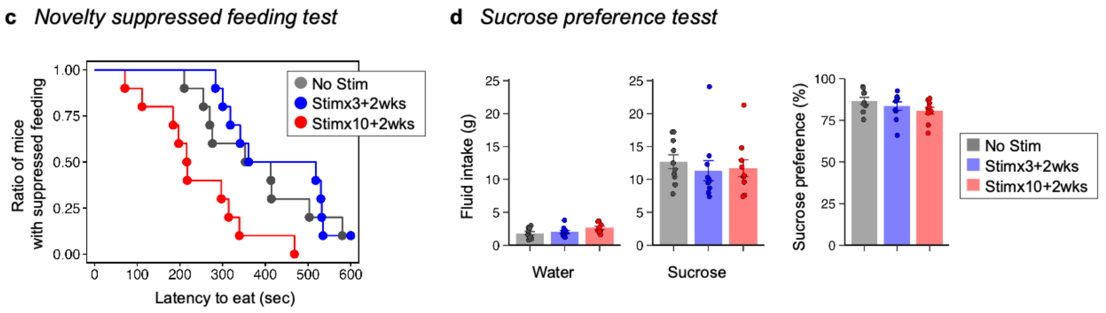

[Original: ---]

[Revised: Extended Data Fig. 5c,d, legend] added

Extended Data Figure 5. REPOPS for 10 days induces anti-depressive behavioral changes in mice.

c, Novelty-suppressed feeding test. Latency to feed per mouse shown as Kaplan–Meier survival curves depicting the probability of not eating over time. Log-rank Mantel–Cox analysis: Stim×10 vs. No Stim, $\chi^2 = 5.09$, $P = 0.024$; Stim×10 vs. Stim×3, $\chi^2 = 10.20$, $P = 0.0014$; Stim×3 vs. No Stim, $\chi^2 = 0.49$, $P = 0.49$. ANCOVA controlling for locomotor activity revealed a significant main effect of stimulation group on feeding latency ($F_{(2, 26)} = 6.75$, $P = 0.0045$), whereas total distance during the test ($F_{(1, 25)} = 0.72$, $P = 0.40$) and sex ($F_{(1, 25)} = 3.36$, $P = 0.079$) were not significant.

d, Sucrose preference test. Water and sucrose solution intake (g) and sucrose preference (%). Data are presented as mean ± s.e.m.

[Original: ---]

[Revised: Supplementary Results 1] added

Supplementary Result 1. Behavioral analysis of optogenetically stimulated mice (related to Extended Data Figs. 5 and 6)

To further examine whether REPOPS alleviates depression-related behavior, we performed the novelty-suppressed feeding test, a well-established assay for antidepressant-like effects that is less dependent on locomotor activity[5]. In this paradigm, food-deprived mice are placed in a brightly lit open field with a food pellet in the center, and the latency to begin feeding is recorded. Feeding latency differed significantly among the three groups (log-rank Mantel–Cox test: $\chi^2 = 11.86$, $P = 0.0027$). Post hoc pairwise comparisons revealed that feeding latency was significantly shorter in the Stim×10 group than in both the No Stim ($\chi^2 = 5.09$, $P = 0.024$) and Stim×3 groups ($\chi^2 = 10.20$, $P = 0.0014$), whereas no significant difference was detected between the No Stim and Stim×3 groups ($\chi^2 = 0.25$, $P = 0.62$; Extended Data Fig. 5c). To determine whether this effect was attributable to differences in general activity levels, ANCOVA controlling for locomotor activity confirmed that the main effect of stimulation group on feeding latency remained significant ($F_{(2, 18)} = 6.93$, $P = 0.0059$). When sex and the interaction term were further entered into the model, there were no significant effects of locomotor activity ($F_{(1, 18)} = 0.74$, $P = 0.40$), sex ($F_{(1, 18)} = 3.44$, $P = 0.080$), or their interaction ($F_{(2, 18)} = 0.74$, $P = 0.49$). These results demonstrate that 10-day, but not 3-day, REPOPS reduces anxiety-related behavior independently of general locomotor activity. We also performed the sucrose preference test. No statistically significant differences were observed among groups in sucrose or water intake or in the percentage of sucrose preference (Extended Data Fig. 5d). These findings suggest that REPOPS does not alter anhedonia-like behavior, although the high baseline sucrose preference in wild-type mice may have limited the sensitivity of this test.

[Original: Extended Data Fig. 3b]

[Revised: Extended Data Fig. 6b] added

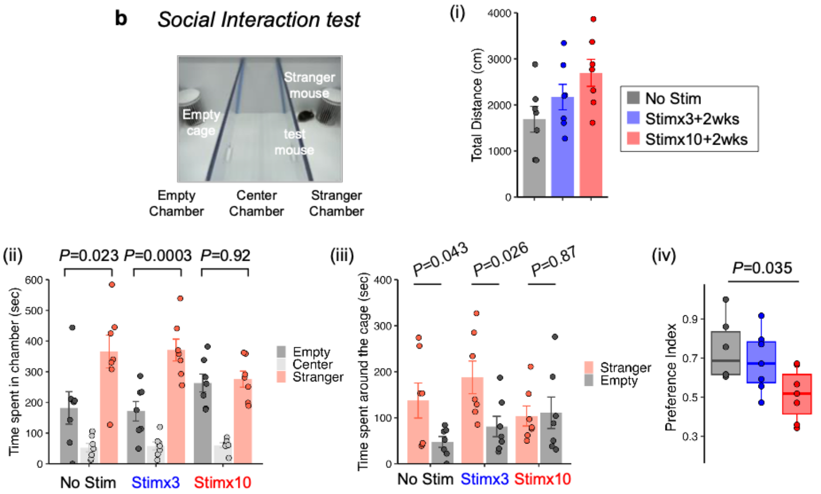

[Original: Extended Data Fig. 6b, legend]

b, Social interaction test. (i) Time spent in each chamber (empty, center, stranger mouse) (n = 7 mice/group). One-way ANOVA: No Stim, $F_{(2,18)} = 12.7$, $P = 3.63 \times 10^{-4}$; Stim×3, $F_{(2,18)} = 30.42$, $P = 1.69 \times 10^{-6}$; Stim×10, $F_{(2,18)} = 27.33$, $P = 3.51 \times 10^{-6}$. P-values in the graphs were Bonferroni corrected for multiple comparisons. (ii) Time spent around the empty cage vs. the cage with a stranger mouse. Student t-test: No Stim, $t_{(12)} = 2.27$, $P = 0.043$; Stim×3, $t_{(12)} = 2.54$, $P = 0.026$; Stim×10, $t_{(12)} = 0.17$, $P = 0.87$. (iii) Preference index, calculated as time spent near the stranger mouse divided by total time spent near both cages. One-way ANOVA, $F_{(2,17)} = 4.18$, $P = 0.0333$. P-values were Bonferroni-corrected for multiple comparisons.

[Revised: Extended Data Fig. 6b, legend]

b, Social interaction test. (i) Locomotor activity (n = 7 mice per group). One-way ANOVA: No Stim, $F_{(2, 18)} = 3.172$, $P = 0.0661$. (ii) Time spent in each chamber (empty, center, stranger mouse) (n = 7 mice/group). One-way ANOVA: No Stim, $F_{(2, 18)} = 12.7$, $P = 3.63 \times 10^{-4}$; Stim×3, $F_{(2, 18)} = 30.42$, $P = 1.69 \times 10^{-6}$; Stim×10, $F_{(2, 18)} = 27.33$, $P = 3.51 \times 10^{-6}$. P-values in the graphs were Bonferroni corrected for multiple comparisons. Observed powers (1−β) were 0.96 (No Stim), 0.99 (Stim×3), and 0.99 (Stim×10). (iii) Time spent around the empty cage vs. the cage with a stranger mouse. Student t-test: No Stim, $t_{(12)} = 2.27$, $P = 0.043$; Stim×3, $t_{(12)} = 2.54$, $P = 0.026$; Stim×10, $t_{(12)} = 0.17$, $P = 0.87$. Observed powers (1−β) were 0.78 (No Stim), 0.85 (Stim×3), and 0.06 (Stim×10). (iv) Preference index, calculated as time spent near the stranger mouse divided by total time spent near both cages. One-way ANOVA, $F_{(2, 17)} = 4.18$, $P = 0.0333$. P-values were Bonferroni-corrected for multiple comparisons.

[Original: Supplementary Results 1]

We assessed the impact of REPOPS on sociability using a social interaction test (Extended Data Fig. 3b). Mice in the No Stim and Stim×3 groups spent significantly more time in the chamber with stranger mice than in the chamber with empty cages (Extended Data Fig. 3b (i)). In contrast, no significant difference was observed in the time spent in the stranger and empty chambers in the Stim×10 group (Extended Data Fig. 3b (i)). A similar trend was observed for time spent around the cages (Extended Data Fig. 3b (ii)). The preference index (i.e., the ratio of time spent in the stranger cage to the total time) was significantly smaller in Stim×10 group than that in No Stim group (P = 0.034; Extended Data Fig. 3b (iii)). These observations suggest that REPOPS reduces a preference for social novelty in mice.

[Revised: Supplementary Results 1]

Supplementary Result 1. Behavioral analysis of optogenetically stimulated mice (related to Extended Data Figs. 5 and 6)

We assessed the impact of REPOPS on sociability using a social interaction test (Extended Data Fig. 6b). Mice in the No Stim and Stim×3 groups spent significantly more time in the chamber with stranger mice than in the chamber with empty cages (Extended Data Fig. 6b (i)). In contrast, no significant difference was observed in the time spent in the stranger and empty chambers in the Stim×10 group (Extended Data Fig. 6b (i)). A similar trend was observed for time spent around the cages (Extended Data Fig. 6b (ii)). The preference index (i.e., the ratio of time spent in the stranger cage to the total time) was significantly smaller in Stim×10 group than that in No Stim group (P = 0.034; Extended Data Fig. 6b (iii)). These observations suggest that REPOPS reduces a preference for social novelty in mice. However, it is also possible that increased locomotor activity influenced the social interaction measures. As previously suggested, an enhanced exploratory drive may outweigh attraction to social novelty, leading to an apparent reduction in social preference indices[6]. Considering that locomotor activity during the social interaction test was moderately elevated in the REPOPS group (Extended Data Fig. 6c), both interpretations remain plausible and the results should be interpreted with caution.

Major Comment 2

Lack of disease-relevant models: The authors aim to model ECT, which is used clinically for patients with psychiatric disorders, yet they only test REPOS in healthy mice. To strengthen the translational value of this study, REPOS should be tested in mouse depression-like models such as chronic variable stress or social defeat stress model. Demonstrating that REPOS reduces

depression-like behavior in stressed mice would provide stronger evidence that REPOS mimics ECT's therapeutic and molecular effects.

Response:

As the reviewer noted, validating the effects of REPOPS in a depression-like model is crucial for establishing its translational relevance. To address this, we applied REPOPS to a chronic corticosterone (CORT) administration model, a well-established paradigm that induces depression-like behaviors through sustained activation of the hypothalamic-pituitary-adrenal (HPA) axis (Shoji et al., Mol. Brain., 2024).

In the open field test, CORT-treated mice showed a marked reduction in locomotor activity compared with vehicle controls. REPOPS did not fully restore the overall activity. Five mice (2 CORT-mice and 3 CORT/Stim×10 mice) showed severe CORT-induced debilitation and died soon after the test before subsequent behavioral assessments (arrows in Extended Data Fig. 17b), potentially due to the combination of surgery and CORT-treatment. When these mice that died were excluded from the analysis, locomotor activity in the REPOPS-treated group was significantly higher than in the CORT-only group. Thus, REPOPS may have potentially increased locomotor activity under CORT-induced conditions, but its effect was likely masked, at least in part, by the inclusion of severely debilitated mice.

In the tail suspension test, CORT treatment increased immobility relative to controls, whereas REPOPS reduced this immobility. By contrast, the forced swim and sucrose preference tests showed no clear effects of either chronic CORT treatment or REPOPS. Together, these results suggest that the CORT-induced decreases in locomotor activity and depression-like behaviors were partially rescued by REPOPS.

In the novelty-suppressed feeding test, CORT treatment prolonged feeding latency, whereas REPOPS shortened this latency toward control levels. This finding indicates that REPOPS alleviated anxiety-like behavior induced by chronic CORT exposure.

These new data, presented in Extended Data Fig. 17 and described in Supplementary Result 3, provide strong evidence that REPOPS ameliorates depression-like behaviors under pathological conditions, thereby strengthening its translational validity and supporting the notion that REPOPS reproduces key therapeutic features of ECT.

Change:

[Original: ---]

[Revised: Extended Data Fig. 17] added

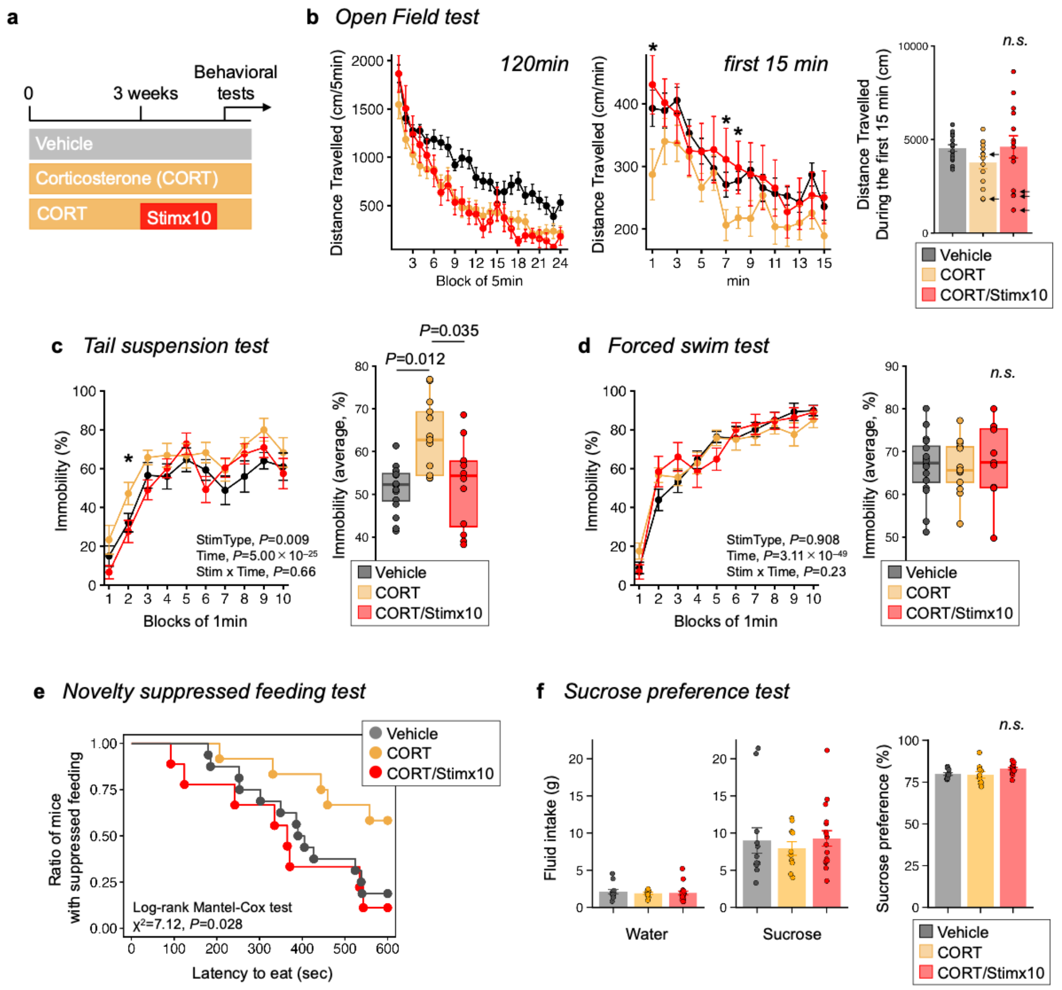

[Original: ---]

[Revised: Extended Data Fig. 17, legend] added

Extended Data Figure 17. REPOPS induces anti-depressive behavioral changes in chronic corticosterone-treated mice.

a, Experimental design: corticosterone (CORT) or vehicle was administered in drinking water for three weeks, followed by REPOPS.

b, Distance traveled in the open field test during 120 min and in the first 15 min. Two-way repeated-measures ANOVA with Bonferroni correction; *$P < 0.05$. Vehicle: 16 mice; CORT: 14 mice; CORT/Stim×10: 14 mice. Arrows indicate mice that became debilitated during CORT administration and died after the experiment.

c, Immobility (%) in the tail suspension test. Mean ± s.e.m. Two-way repeated-measures ANOVA results shown in the panel. Box plot summarizes average immobility over 10 min. One-

way ANOVA with Bonferroni correction. Vehicle: 16 mice; CORT: 13 mice; CORT/Stim×10: 12 mice.

d, Same as **c**, for immobility in the forced swim test. Vehicle: 16 mice; CORT: 13 mice; CORT/Stim×10: 11 mice.

e, Novelty-suppressed feeding test. Latency to feed is shown as Kaplan–Meier survival curves depicting the probability of not eating over time. Log-rank Mantel-Cox test: $\chi^2 = 7.12$, $P = 0.028$. Pairwise comparisons revealed significant differences between the Vehicle and CORT groups ($P = 0.025$) and between the CORT and CORT/Stim×10 groups ($P = 0.014$). Vehicle: 16 mice; CORT: 14 mice; CORT/Stim×10: 14 mice.

f, Sucrose preference test. Water and sucrose solution intake (g) and percentage of sucrose preference (%). Mean ± s.e.m.

[Original: ---]

[Revised: Supplementary Results 3] added

Supplementary Result 3. REPOPS reduces depression-like behavior in chronic corticosterone-treated mice (related to Extended Data Fig. 17).

To examine whether the antidepressant-like behavioral effects of REPOPS could be reproduced under depression-like conditions, we used a chronic corticosterone (CORT) administration model**,** a validated paradigm that reliably induces depression-like phenotypes via sustained activation of the hypothalamic–pituitary–adrenal (HPA) axis[7]. Mice received chronic CORT treatment for three weeks, followed by ten consecutive days of REPOPS (CORT/Stim×10 group) or no stimulation (CORT group) (Extended Data Fig. 17a).

In the open field test, mice in the CORT and CORT/Stim×10 groups exhibited markedly reduced locomotor activity compared with controls (Extended Data Fig. 17b). Immediately after exposure to the open field, mice in the CORT/Stim×10 group showed higher locomotor activity than in the CORT group at 1, 7, and 8 min of the test (Extended Data Fig. 17b). There was no significant difference in total distance traveled among the three groups. However, after excluding mice that showed severe CORT-related debilitation and died shortly after the open field test (arrows in Extended Data Fig. 17b), locomotor activity in the CORT/Stim×10 group was significantly higher than in the CORT group ($P = 0.0166$). These results suggest that REPOPS increased locomotor activity under CORT-induced conditions (Extended Data Fig. 17b). In the tail suspension test, CORT-treated mice showed significantly increased immobility relative to vehicle controls ($P = 0.012$), whereas immobility was significantly decreased in the CORT/Stim×10 group compared to CORT-treated mice ($P = 0.035$) (Extended Data Fig. 17c). When sex was included as a covariate, ANCOVA still confirmed a significant main effect of the

stimulation group on locomotor activity in the open field test ($F_{(2, 34)}$ = 6.39, P = 0.0044) and on immobility in the tail suspension test ($F_{(2, 35)}$ = 5.97, P = 0.0059). There were no significant changes in immobility in the forced swim test (Extended Data Fig. 17d).

In the novelty-suppressed feeding test, feeding latency differed significantly among the three groups (log-rank Mantel–Cox test: χ^2 = 7.12, P = 0.028). Post hoc pairwise comparisons revealed that feeding latency was significantly longer in the CORT group than in controls (χ^2 = 5.00, P = 0.025), whereas the CORT/Stim×10 group exhibited a significantly shorter latency than in the CORT group (χ^2 = 6.01, P = 0.014) (Extended Data Fig. 17e). When sex was included as a covariate, ANCOVA still confirmed a significant main effect of the stimulation group on feeding latency ($F_{(2, 35)}$ = 3.41, P = 0.046). Together, these data demonstrate that REPOPS reverses a certain type of CORT-induced depression- and anxiety-like behavioral deficits and supports the notion that REPOPS recapitulates key therapeutic effects of ECT under pathological conditions.

[Original: ---]

[Revised: L1091] added

Corticosterone treatment

Corticosterone (95%, FUJIFILM Wako Pure Chemical Co., Japan) was dissolved in ethanol (99.5%, FUJIFILM Wako Pure Chemical Co., Japan) at 10 mg/mL and then diluted with filtered drinking water to a final concentration of 0.1 mg/mL corticosterone in 1% ethanol. Mice had ad libitum access to bottles containing either the CORT or vehicle solution. The solutions were replaced every 2–4 days. Oral administration continued throughout the experiment except during the sucrose preference test.

Major Comment 3

Cell-type heterogeneity in RNA-seq: Bulk RNA-seq and ATAC-seq of DG tissue includes multiple cell types other than granule cells (GC), the main target cells of this study. I highly recommend using an external single-cell RNA-seq dataset of mouse hippocampus (e.g. PMID: 37034735, PMID: 38217668, https://portal.brain-map.org/atlases-and-data/rnaseq/mouse-wholecortex-and-hippocampus-smart-seq) to adjust cell-type composition in differential models and use deconvolution approaches to further clarify whether dematuration signatures are specific to GC.

Response:

We appreciate the reviewer's important comment regarding cell-type heterogeneity. To address this, we performed a cell-type enrichment analysis using a dentate gyrus-specific single-cell RNA-

Editorial Note: Supplementary Fig. 15 in this Peer Review File is reproduced with permission from BioRender, Created in BioRender. Murano, T. (2026) https://BioRender.com/p6ed2mm

seq reference dataset (Hochgerner et al., Nat. Neurosci., 2018). This analysis revealed that REPOPS-induced transcriptional changes were predominantly enriched in granule cell– and neuroblast-specific genes, with moderate enrichment also observed for glial cell–associated genes. These findings indicate that the dematuration-like transcriptomic changes observed in bulk RNA-seq largely originate from granule cells. The new results are presented in Extended Data Figure 15 and described in the Supplementary Result 2.

Change:

[Original: ---]

[Revised: Extended Data Fig. 15] added

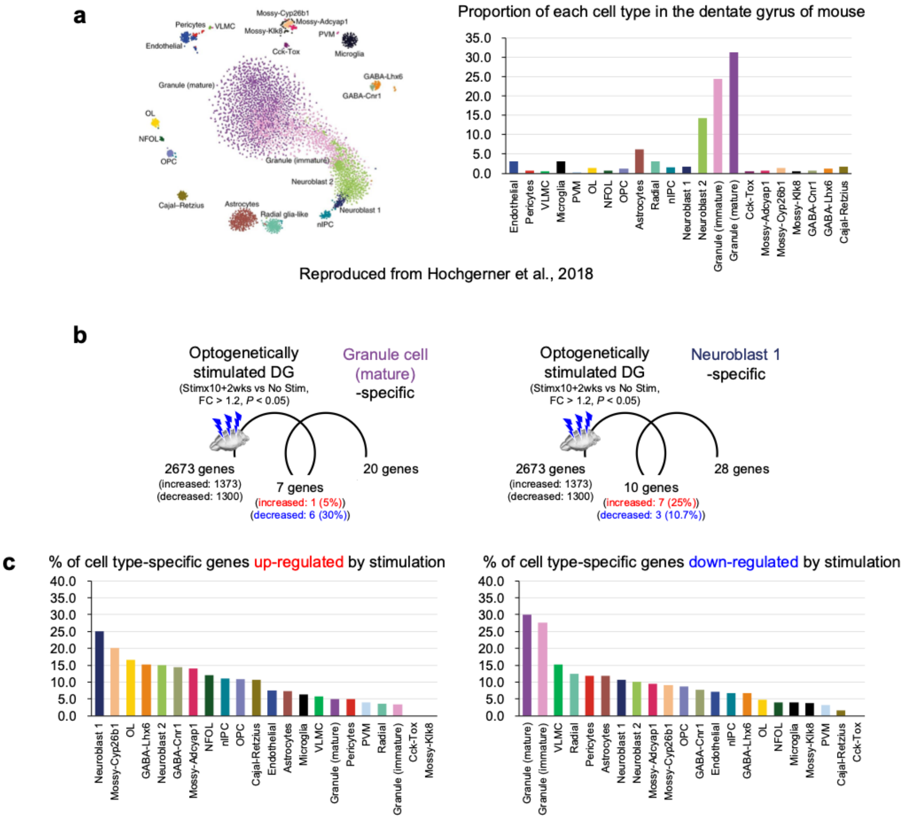

[Original: ---]

[Revised: Extended Data Fig. 15, legend] added

Extended Data Figure 15. REPOPS induces transcriptomic changes across multiple cell types, with predominant effects on granule cells.

a, Left, visualization of single-cell clusters in the mouse dentate gyrus, color-coded by cell-type cluster annotation (*reproduced from* Hochgerner *et al., Nat. Neurosci.,* 2018[3]). Right, proportion of each cell type relative to the total cell population.

b, Venn diagram showing the overlap between granule cell (mature)– or neuroblast-specific genes and genes significantly altered in the Stim×10+2wks group.

c, Bar plot illustrating the proportion of genes significantly up- or down-regulated in the Stim×10+2wks group within each cell type–specific gene set. Cell type classifications on the x-axis are based on Hochgerner *et al.,* 2018.

[Original: ---]

[Revised: Supplementary Result 2] added

Supplementary Result 2. REPOPS induces transcriptomic changes across multiple cell types, with predominant effects on granule cells (related to Extended Data Fig. 15).

Our bulk RNA-seq analysis (Fig. 1c–f) captured gene expression changes not only in granule cells (GCs) but also in other cell types, including mossy cells, interneurons, and glial cells. To estimate the relative contribution of each cell type, we performed a cell-type enrichment analysis using the dentate gyrus single-cell RNA-seq dataset reported by Hochgerner et al.[3]. Extended Data Fig. 15a shows the cell-type annotations and their relative proportions within the DG, identical to those in the original reference. Marker genes for each cell type were defined as those expressed >10-fold higher in that specific cell type than the mean across all others. We compared these marker genes with the differentially expressed genes identified in the Stim×10+2wks group and calculated the proportion of marker genes that were significantly up- or down-regulated for each cell type (Extended Data Fig. 15b). Genes associated with immature excitatory neurons (Neuroblast1 cluster) were most prominently upregulated, whereas those associated with mature granule cells were most downregulated (Extended Data Fig. 15c). Cell-type-specific genes for other populations (e.g., glial and mossy cells) also showed moderate alterations, indicating that REPOPS broadly influences multiple cellular populations within the DG.

To further validate these findings, we performed immunostaining for NeuN, parvalbumin (PV), GFAP, and Iba1 and quantified the density of marker-positive cells (Extended Data Fig. 15d). None of these markers showed significant differences in the number of positive cells after REPOPS, indicating that the bulk-level transcriptomic changes are unlikely to be explained simply by large shifts in cell numbers. We also analyzed the mRNA expression of representative cell-type marker genes (Prox1 and Calb1 for granule cells, Calb2 for mossy cells, Pvalb and Sst for interneurons, Gfap for astrocytes, and Aif1 (Iba1) for microglia).

REPOPS for 10 days significantly reduced the expression of Prox1, Calb1, and Iba1, increased Gfap expression, and did not change the expression of Calb2, Pvalb, or Sst (Extended Data Fig. 15e). Given that granule cells are overwhelmingly predominant within the DG cell composition (Extended Data Fig. 15a, d), these results support the conclusion that, although REPOPS affects multiple cell types, the majority of transcriptomic alterations observed in our bulk RNA-seq data are primarily driven by granule cells.

Major Comment 4

ATAC-seq quality control: No QC metrics are shown for ATAC-seq. Please provide transcription start site (TSS) enrichment plots, fragment-in-peaks (FRiP) scores, and details on peak merging and peak reproducibility across samples. Please provide details on which method or pipeline was used for transcription factor motif enrichment of differential ATAC-seq peaks in the Methods section.

Response:

We thank the reviewer for this important comment. To ensure transparency and reproducibility, we have added detailed quality control metrics and methodological information for the ATAC-seq analysis in Extended Data Fig. 16 and the Methods section.

(i) **FRiP scores:** ranged from 37–51% across all samples, well above the commonly accepted 10% threshold, confirming high data quality.

(ii) **TSS enrichment:** aggregate accessibility profiles centered on transcription start sites (±5 kb), genebodies (±2 kb), and merged peak regions (±5 kb), showed strong enrichment across all samples, indicating a high signal-to-noise ratio and robust library quality (Extended Data Fig. 16).

(iii) **Peak merging and reproducibility:** peaks called by MACS2 were merged across samples by taking the union of overlapping intervals, enabling consistent quantification even when peak boundaries slightly differed between replicates.

(iv) **Motif enrichment:** differentially accessible regions (adjusted $P < 0.1$) were analyzed using HOMER with a 200 bp windows centered on peak midpoints (±100 bp); both known and de novo motifs were identified after repeat masking to minimize false positives.

Change:

[Original: ---]

[Revised: Extended Data Fig. 16] added

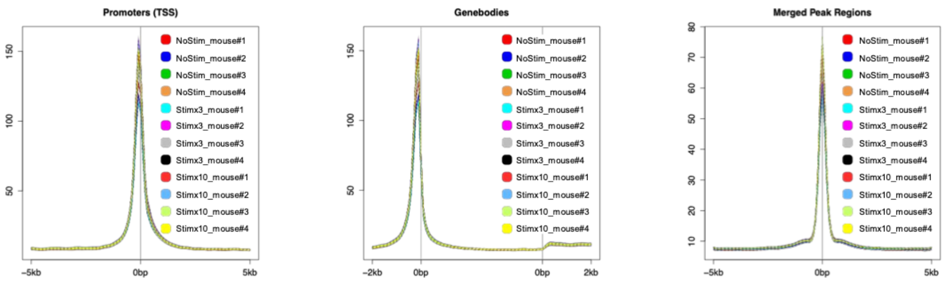

[Original: ---]

[Revised: Extended Data Fig. 16, legend] added

Extended Data Figure 16. ATAC-seq signal enrichment and distribution across genomic features.

a, Aggregate accessibility profiles centered on transcription start sites (TSS ± 5 kb) for all samples.

b, Aggregate accessibility profiles aligned to genebodies (±2 kb), showing the signal distribution along gene bodies.

c, Aggregate accessibility profiles centered on merged peak regions (±5 kb), illustrating chromatin accessibility around consensus peak centers.

[Original: L784]

ATAC-Sequencing Library Construction, Sequencing, Mapping, and Data Analysis

The dentate gyrus of adult mice was dissected in the same manner as that used for RNA-seq. The dentate gyrus was slowly frozen in a freezing box (Cat. No. 5100-0001; Mr. Frosty Freezing Container, Thermo Fisher Scientific). Flash-frozen tissue was sent to Active Motif to perform the ATAC-seq assay. The tissue was manually dissociated, isolated nuclei were quantified using a hemocytometer, and 100,000 nuclei were tagmented as previously described[110], with some modifications based on Corces et al. 2017[111] using the enzyme and buffer provided in the Nextera Library Prep Kit (Illumina). Tagmented DNA was purified using the MinElute PCR purification kit (Qiagen), amplified with 10 cycles of PCR, and purified using Agencourt AMPure SPRI beads (Beckman Coulter). The resulting material was quantified using the KAPA Library

Quantification Kit for Illumina platforms (KAPA Biosystems) and sequenced using PE42 sequencing on a NextSeq 500 sequencer (Illumina). For the analysis of ATAC-seq data, we first aligned reads using the BWA algorithm (mem mode; default settings). Duplicate reads were removed, and only reads mapping as matched pairs and uniquely mapped reads (mapping quality >= 1) were used for further analysis. Alignments were extended in silico at their 3'ends to a length of 200 bp and assigned to 32-nt bins along the genome. The resulting histograms (genomic "signal maps") were stored in the bigWig files. Peaks were identified using the MACS 2.1.0 algorithm at a cutoff of P-value 1.0×10^{-7}, without the control file, and with the –nomodel option. Peaks on the ENCODE blacklist of known false ChIP-Seq peaks were excluded. Signal maps and peak locations were used as input data for Active Motif's proprietary analysis program, which creates Excel tables containing detailed information on sample comparisons, peak metrics, peak locations, and gene annotations. For differential analysis, reads were counted in all merged peak regions (using Subread) and the replicates for each condition were compared using DESeq2. IGV was used to visualize the raw intensities[112]. Statistical analyses were performed using an in-house R script, unless otherwise specified.

[Revised: L898]

ATAC-Sequencing Library Construction, Sequencing, Mapping, and Data Analysis

ATAC-seq was performed using n = 4 biological replicates per group. The dentate gyrus of adult mice was dissected in the same manner as that used for RNA-seq. The dentate gyrus was slowly frozen in a freezing box (Cat. No. 5100-0001; Mr. Frosty Freezing Container, Thermo Fisher Scientific). Flash-frozen tissue was sent to Active Motif to perform the ATAC-seq assay. The tissue was manually dissociated, isolated nuclei were quantified using a hemocytometer, and 100,000 nuclei were tagmented as previously described[119], with some modifications based on Corces et al. 2017[120] using the enzyme and buffer provided in the Nextera Library Prep Kit (Illumina). Tagmented DNA was purified using the MinElute PCR purification kit (Qiagen), amplified with 10 cycles of PCR, and purified using Agencourt AMPure SPRI beads (Beckman Coulter). The resulting material was quantified using the KAPA Library Quantification Kit for Illumina platforms (KAPA Biosystems) and sequenced using paired-end 42 bp sequencing on a NextSeq 500 sequencer (Illumina).

Reads were aligned to the mm10 genome using BWA (mem mode, default parameters). Duplicate reads were removed, and only reads mapping as matched pairs and uniquely mapped reads (mapping quality >= 1) were used for further analysis. Alignments were extended in silico at their 3'ends to a length of 200 bp and assigned to 32-nt bins along the genome. The resulting histograms (genomic "signal maps") were stored in the bigWig files. Peaks were identified using the MACS 2.1.0 algorithm at a cutoff of P-value 1.0×10^{-7}, without

the control file, and with the –nomodel option. Peaks on the ENCODE blacklist of known false ChIP-Seq peaks were excluded. Intervals identified by MACS2 were merged by taking the union of overlapping peaks across all samples, ensuring consistent quantification among replicates. Read counts within merged regions were obtained using Subread, and differential accessibility was analyzed using DESeq2 (adjusted $P < 0.1$). Quality control metrics confirmed robust data quality: FRiP scores ranged from 37 to 51%, exceeding the standard 10% benchmark. Aggregate accessibility profiles around promoters (TSS ± 5 kb) showed clear enrichment in all samples, confirming high signal-to-noise ratios (Extended Data Fig. 16). Significantly up- and down-regulated regions were exported as BED files, and 200-bp window centered on each peak midpoint were analyzed for motif enrichment using HOMER. Both known and de novo motifs were identified after repeat masking to minimize false positives. Correlation analysis further confirmed high reproducibility across biological samples. Statistical analyses were performed using an in-house R script, unless otherwise specified.

Major Comment 5

ATAC-seq interpretation: The ATAC-seq results are not integrated with RNA-seq findings. Differential peaks should be assigned to genes (e.g. preferably using Hi-C promoter-enhancer connections or other gene assignment methods). Pathway analysis of peak-associated genes could reveal consistency with RNA-seq pathways (e.g. cell cycle/mitosis). Statistical comparison between ATAC- and RNA-seq derived gene sets should be done to provide deeper insights into chromatin-transcriptome relationships induced by REPOS.

Response:

We appreciate the reviewer’s insightful suggestion. To address this, we performed an integrated analysis of the ATAC-seq and RNA-seq datasets.

For ATAC-seq peaks showing significant changes in chromatin accessibility ($P < 0.05$), each peak was assigned to its nearest gene, and the corresponding genes were defined as ATAC-seq peak–associated genes. This gene-assignment approach revealed a high degree of concordance between the ATAC-seq and RNA-seq datasets, supporting its biological validity (Extended Data Fig. 4).

A running Fisher test further demonstrated a highly significant overlap between peak-associated and differentially expressed genes ($P = 4.9 \times 10^{-103}$), indicating that chromatin accessibility changes are tightly coupled to transcriptional regulation. These results are presented in Extended Data Fig. 4.

Furthermore, pathway analysis of the ATAC-seq peak–associated genes revealed enrichment of pathways consistent with those identified in the RNA-seq data—most notably, those related to

the cell cycle and mitosis (e.g., “regulation of mitotic cell cycle”). The complete list of enriched pathways is provided in Supplementary Table 3.

Change:

[Original: ---]

[Revised: Extended Data Fig. 4] added

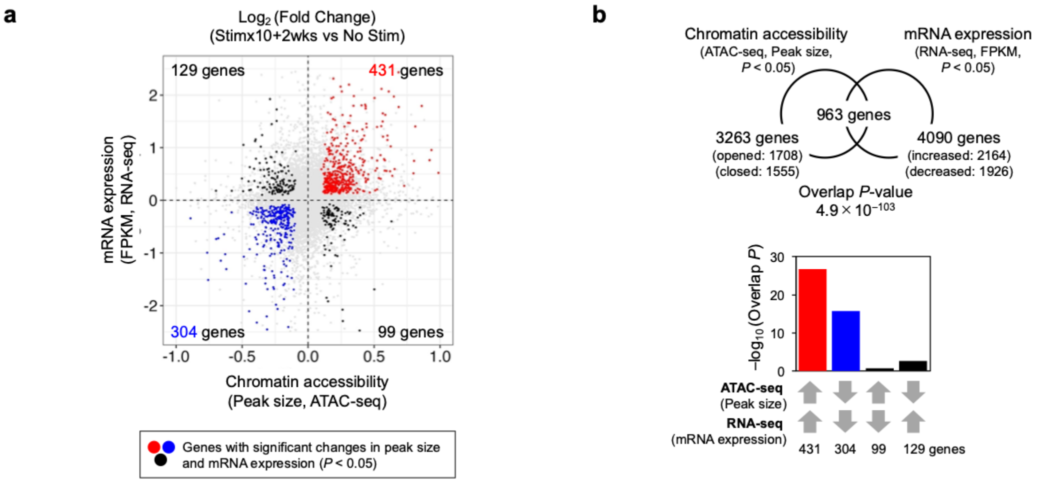

[Original: ---]

[Revised: Extended Data Fig. 4, legend] added

Extended Data Figure 4. Concordance between changes in gene expression and chromatin accessibility.

a, Scatter plot showing the fold change in chromatin accessibility (ATAC-seq peak size, Stim×10+2wks vs. No Stim) versus mRNA expression (RNA-seq, Stim×10+2wks vs. No Stim). Peak size for each gene was calculated as the sum of read counts mapped to the genebody ± 1 kb. Red, blue, and black dots represent genes showing significant increases, decreases, or no change in both chromatin accessibility and mRNA expression ($P < 0.05$).

b, Overlap between genes with significant changes in chromatin accessibility and mRNA expression (Stim×10+2wks vs. No Stim). The bar graph shows the $-\log_{10}$ of overlap *P*-values for up- and down-regulated genes, corresponding to the color scheme in **a**.

[Original: L157]

These results indicate that repeated neuronal activation for 10 consecutive days, but not for 3 days, induced widespread and persistent reorganization of three-dimensional genome structure, along with concordant global transcriptome changes.

[Revised: L188]

These results indicate that repeated neuronal activation for 10 consecutive days, but not for 3 days, induced widespread and persistent reorganization of three-dimensional genome structure, along with concordant global transcriptome changes (Extended Data Fig. 4 and Supplementary Table 3).

[Original: ---]

[Revised: Supplementary Table 3] added

Supplementary Table 3. Pathway analysis of gene sets altered by REPOPS (Stim×10+2wks): genes identified from RNA-seq, genes associated with ATAC-seq peak changes, and their combined gene set.

Major Comment 6

Sex specific analysis: It is not clear whether the experiments performed in this study were using male or female mice or both. If experiments performed were only in the male mice, it would be a missed opportunity to not test molecular and behavioral effects of REPOS in female mice. This is important because psychiatric disorders tend to effect males and females differently. I highly recommend performing at least some of the same experiments (e.g. Calbindin expression, locomotor activity, RNA-seq) to show consistent outcomes in female mice as well.

Response:

We apologize for not providing sufficient information regarding the sex of the animals in the original manuscript and thank the reviewer for raising this important point.

RNA-seq and ATAC-seq analyses were performed using male mice. Other experiments were conducted using approximately equal numbers of male and female mice. This information has been added to the Methods section.

In the additional experiments−including NSFT and SPT (Extended Data Fig. 5, response to major comment 1) and the CORT model (Extended Data Fig. 17; response to major comment 2)−both sexes were also included in nearly equal numbers. In all behavioral analyses, sex was included as a factor in ANCOVA to statistically evaluate its influence. No significant main effects of sex or sex-by-stimulation group interactions were detected.

These findings suggest that the behavioral effects of REPOPS are not sex-dependent. The corresponding information has been added to the methods sections, Extended Data Figures, and Supplementary Results.

Change:

[Original: L683]

Animals

… Mice were used for experiments at 3–4 months of age for RNA-seq and ATAC-seq experiments, and at 6–12 months for other experiments. …

[Revised: 767]

Animals

… For RNA-seq and ATAC-seq experiments, male mice aged 3–4 months were used. For other experiments, approximately equal numbers of male and female mice aged 6–12 months were used. Age-matched control groups were included in each experiment. Our previous study (ref: Shoji et al., Neuropsychopharmacol Rep 2019)[113] reported minimal behavioral differences between 2- and 11-month-old mice, indicating that the use of 3–4 month-old and 6–12 month-old mice in the present study is unlikely to confound the interpretation of the results. For the omics analyses, six mice per group were used for RNA-seq and four per group for ATAC-seq, which is consistent with standard practice in transcriptomic and epigenomic studies (ref: Su et al., Nat. Neurosci., 2017; Fernández et al., Nat. Neurosci., 2019)[27,28]. For behavioral experiments, 7–16 mice per group were used. Post hoc power analyses (α = 0.05) based on the observed effect sizes indicated that the achieved statistical powers (1−β) exceeded 0.80 in most tests, confirming that the sample sizes were sufficient to detect the main effects.

[Original: ---]

[Revised: Supplementary Result 1] added

Supplementary Result 1. Behavioral analysis of optogenetically stimulated mice (related to Extended Data Figs. 5 and 6)

… To determine whether this effect was attributable to differences in general activity levels, ANCOVA controlling for locomotor activity confirmed that the main effect of stimulation group on feeding latency remained significant ($F_{(2,\ 18)}$ = 6.93, P = 0.0059). When sex and the interaction term were further entered into the model, there were no significant effects of locomotor activity ($F_{(1,\ 18)}$ = 0.74, P = 0.40), sex ($F_{(1,\ 18)}$ = 3.44, P = 0.080), or their interaction ($F_{(2,\ 18)}$ = 0.74, P = 0.49). These results demonstrate that 10-day, but not 3-day, REPOPS

reduces anxiety-related behavior independently of general locomotor activity. We also performed the sucrose preference test…

[Original: ---]

[Revised: Supplementary Results 3] added

Supplementary Result 3. REPOPS reduces depression-like behavior in chronic corticosterone-treated mice (related to Extended Data Fig. 17).

In the open field test, mice in the CORT and CORT/Stim×10 groups exhibited markedly reduced locomotor activity compared with controls (Extended Data Fig. 17b). Immediately after exposure to the open field, mice in the CORT/Stim×10 group showed higher locomotor activity than in the CORT group at 1, 7, and 8 min of the test (Extended Data Fig. 17b). There was no significant difference in total distance traveled among the three groups. However, after excluding mice that showed severe CORT-related debilitation and died shortly after the open field test (arrows in Extended Data Fig. 17b), locomotor activity in the CORT/Stim×10 group was significantly higher than in the CORT group (P = 0.0166). These results suggest that REPOPS increased locomotor activity under CORT-induced conditions (Extended Data Fig. 17b). In the tail suspension test, CORT-treated mice showed significantly increased immobility relative to vehicle controls (P = 0.012), whereas immobility was significantly decreased in the CORT/Stim×10 group compared to CORT-treated mice (P = 0.035) (Extended Data Fig. 17c). When sex was included as a covariate, ANCOVA still confirmed a significant main effect of the stimulation group on locomotor activity in the open field test ($F_{(2, 34)}$ = 6.39, P = 0.0044) and on immobility in the tail suspension test ($F_{(2, 35)}$ = 5.97, P = 0.0059). There were no significant changes in immobility in the forced swim test (Extended Data Fig. 17d).

In the novelty-suppressed feeding test, feeding latency differed significantly among the three groups (log-rank Mantel–Cox test: χ^2 = 7.12, P = 0.028). Post hoc pairwise comparisons revealed that feeding latency was significantly longer in the CORT group than in controls (χ^2 = 5.00, P = 0.025), whereas the CORT/Stim×10 group exhibited a significantly shorter latency than in the CORT group (χ^2 = 6.01, P = 0.014) (Extended Data Fig. 17e). When sex was included as a covariate, ANCOVA still confirmed a significant main effect of the stimulation group on feeding latency ($F_{(2, 35)}$ = 3.41, P = 0.046). Together, these data demonstrate that REPOPS reverses a certain type of CORT-induced depression- and anxiety-like behavioral deficits and supports the notion that REPOPS recapitulates key therapeutic effects of ECT under pathological conditions.

Major Comment 7

Comparison to patient ECT data: The authors only compared a subset of selected synaptic genes between REPOS and ECT-treated patients. A more comprehensive comparison of differential gene expression between 10-day REPOS vs non-stimulated mice and ECT-treated vs untreated patients is required to provide unbiased insight into whether REPOS truly parallels ECT at the molecular level.

Response:

We agree with the reviewer that a comprehensive and unbiased comparison between REPOPS and ECT is essential. To address this, we compared the differentially expressed genes identified in Figure 1d (Stim×10+24hrs/2wks vs No Stim) and Figure 1f (ECT-treated patients vs untreated patients/healthy controls). The list of overlapping genes and associated gene ontology terms were compiled in Supplementary Table 2.

Specifically, 138 genes were commonly altered between the Stim×10+2wks/No Stim comparison and the ECT-treated/non-treated patient comparison. The overlap *P*-values for genes changing in the same direction (up–up or down–down; $P = 0.192$, 5.8×10^{-5}) were relatively smaller than those for genes changing in the opposite direction (up–down or down–up; $P = 0.0006$, 0.603), indicating that REPOPS and ECT elicit overall concordant changes in gene expression profiles. Notably, genes and gene groups related to synaptic function and cell-cycle regulation were commonly affected in both datasets, underscoring a strong molecular parallel between REPOPS and ECT.

Change:

[Original: L142]

> This analysis revealed a significant overlap between the two datasets (Overlap $P = 1.2 \times 10^{-8}$), with consistent directional changes (Fig. 1f), suggesting that ECT induces an immature-like gene expression pattern in the DG of human patients, in line with the pattern observed in REPOPS.

[Revised: L144]

> This analysis revealed a significant overlap between the two datasets (Overlap $P = 1.2 \times 10^{-8}$), with consistent directional changes (Fig. 1f), suggesting that ECT induces an immature-like gene expression pattern in the DG of human patients, in line with the pattern observed in REPOPS (see also Supplementary Table 2).

[Original: ---]

[Revised: Supplementary Table 2] added

Supplementary Table 2. Genes and related GO terms commonly altered in REPOPS (Stim×10+2wks) mice and ECT patients.

Major Comment 8

G2/M 're-entry' in mature neurons: I think the authors claim of REPOS induced G2/M cell cycle 're-entry' is too strong without further functional evidence (e.g., showing a full panel of mitotic markers (e.g., Ki67, Aurora kinase B, PCNA) or markers of DNA synthesis (e.g., BrdU). Was pH3 staining co-localized with neuronal markers (NeuN, Prox1) to confirm this occurs in mature neurons and not in glial or progenitor cells? Does cyclin B+, lamin-disruption, and pH3+ neurons overlap spatially? This co-localization would strengthen the argument. Currently, it may be more accurate to say, "REPOS upregulates G2/M phase associated genes" or "induces a G2/M-like transcriptional signature." If neurons truly re-entered G2/M transcriptional programs, why was there no evidence of apoptosis detected in TUNEL assay? Aberrant cell cycle re-entry is often associated with neuronal death. For example, aberrant cell cycle re-entry has been shown as a causal phenomenon in neurodegeneration and Alzheimer's disease (PMID: 20584423). This discrepancy requires further investigation and deeper discussion.

Response:

We agree that the term "G2/M re-entry" was too strong. As noted in the original text ("Importantly, we do not assert that this immature-like cellular state is a perfect regression to developmental immaturity but rather an analog state of immaturity …", L386), we do not interpret these changes as true cell division but rather as a G2/M phase-like transcriptional state reflecting partial activation of cell cycle-associated genes. Accordingly, we have revised the wording "G2/M cell cycle re-entry" to the more accurate description, "induces a G2/M-like transcriptional signature," as suggested.

The fixation protocols for lamin B staining was different from those for Cyclin B or pH3 (as described in methods section); therefore, co-staining was not feasible. We performed double immunostaining for Cyclin B and pH3, which revealed that in the Stim×10+2wks group, most pH3-positive cells were localized within the granule cell layer (GCL) and overlapped spatially with Cyclin B-positive cells (Extended Data Fig. 10a). Furthermore, this distribution pattern closely resembled that of NeuN, whereas it was clearly distinct from those of GFAP or Iba1 (Extended Data Fig. 15d), indicating that these signals mainly originated from neurons rather than glial cells. Although a minor contribution of astrocytes or microglia cannot be completely excluded, the increase in pH3 signals appears to occur predominantly in neurons. These new results are presented in Extended Data Fig. 10 and are described in the main text.

As the reviewer pointed out, although aberrant cell cycle re-entry is often linked to neuronal death in pathological contexts, REPOPS induced a G2/M-like transcriptional and structural response without apoptotic features. As discussed in the original text ("REPOPS-induced nuclear structural changes were less pronounced than those in dividing cells during the late M phase", L383), we speculate that REPOPS activates a non-lethal, partial form of the G2/M program that contributes to nuclear plasticity rather than cell death. The Discussion has been revised accordingly to clarify this interpretation.

Change:

[Original: ---]

[Revised: Extended Data Fig. 10a] added

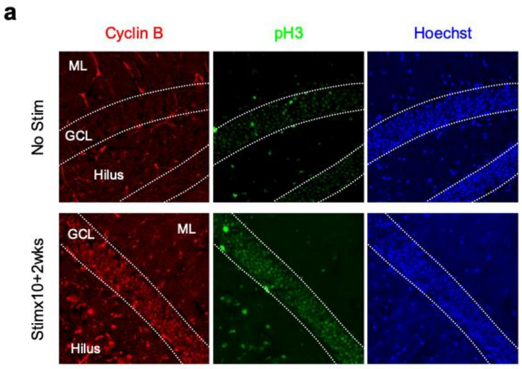

[for reference: Extended Data Fig. 15d]

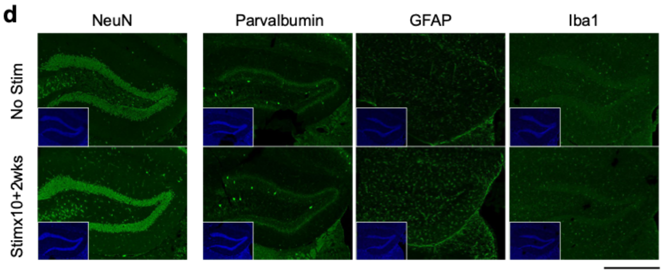

[Original: ---]

[Revised: Extended Data Fig. 10, legend] added

Extended Data Figure 10. Mitosis-like epigenetic modifications are associated with heterochromatin enlargement.

a, Immunostaining of Cyclin B and pH3 in the DG (related to Fig. 5b, f, g). Scale bar, 20 μm. ML, molecular layer; GCL, granule cell layer.

[Original: L279]

Thus, REPOPS induces G_2/M phase-like nuclear states, characterized by nuclear lamina disruption, increased pH3, and chromatin condensation (see also Extended Data Fig. 6a, 6b).

[Revised: L318]

Thus, REPOPS induces G_2/M phase-like nuclear states, characterized by nuclear lamina disruption, increased pH3, and chromatin condensation. These changes largely overlapped and were most prominent in the granule cell layer, suggesting that they occur predominantly in granule cells (see also Extended Data Fig. 10). Interestingly, this G_2/M-like nuclear state aligns with the idea that REPOPS-induced dematuration is likely mediated by RSK/MSK, which regulate chromatin remodeling through histone phosphorylation, and by CDKs, which are key regulators of the cell cycle (Fig. 2f).

[Original: L363]

Our findings reveal that optogenetic stimulation of hippocampal granule cells can trigger coordinated changes in gene expression, nuclear structural plasticity, and G_2/M cell cycle re-entry, which collectively define an immature-like cell state. The evidence supporting G_2/M cell cycle re-entry includes the increased expression of M phase-related genes such as Cyclin A2, Cyclin B1, Cyclin B2, and Cdc25c, and changes in nuclear structure, including nuclear lamina disruption, an increase in pH3, and chromocenter enlargement.

[Revised: L406]

Our findings reveal that optogenetic stimulation of hippocampal granule cells induces a G2/M phase-like transcriptional signature and nuclear structural plasticity, which collectively define an immature-like cell state. There was increased expression of G2/M phase-related genes such as Cyclin A2, Cyclin B1, Cyclin B2, and Cdc25c, and changes in nuclear structure, including nuclear lamina disruption, an increase in pH3, and chromocenter enlargement.

[Original: L379]

This study is the first, to our knowledge, to show a causal link between chronic neural activation and G_2/M cell cycle re-entry.

[Revised: L421]

This study is, to our knowledge, the first to demonstrate a causal link between chronic neural activation and G2/M phase-like transcriptional and nuclear signatures.

[Original: L380]

Interestingly, the cell cycle re-entry observed in various diseases is typically associated with apoptosis[78,79]; however, our REPOPS method did not induce significant cell death (Extended Data Fig. 1c). REPOPS triggered nuclear structural dynamics, but they were less pronounced than those in dividing cells during the late M phase[79,80] and there were no fully compacted chromosomes (Fig. 4e, 4g), suggesting that neurons were arrested in a G_2 to early M phase-like state for at least two weeks. Importantly, we do not assert that this immature-like cellular state is a perfect regression to developmental immaturity but rather an analog state of immaturity, a state that is phenotypically immature relative to the mature state.

[Revised: L423]

Interestingly, the cell cycle re-entry observed in various diseases is typically associated with apoptosis[83,84]; however, our REPOPS method did not induce significant cell death (Extended Data Fig. 2). REPOPS triggered nuclear structural dynamics, but they were less pronounced than those in dividing cells during the late M phase[84,85] and there were no fully compacted chromosomes (Fig. 5e, 5g), suggesting partial activation of G2/M-associated programs rather than true mitotic progression. Importantly, we do not assert that this immature-like cellular state is a perfect regression to developmental immaturity but rather an analog state of immaturity, a state that is phenotypically immature relative to the mature state.

Major Comment 9

Adult neurogenesis markers: Authors conclude cyclin B is expressed in mature neurons rather than newborn cells. It would be valuable to test whether DE genes from REPOS overlap with known markers of adult hippocampal neurogenesis (e.g. doublecortin, nestin, Pax6, NeuroD, calretinin) and perform immunostaining of cyclin B with markers of neurogenesis. This would be important to help distinguish whether REPOS mimics early developmental dematuration or induces adult neurogenesis programs.

Response:

As suggested, we systematically compared REPOPS-induced differentially expressed genes with hippocampal neurogenesis-related genes listed in the Mammalian Adult Neurogenesis Gene Ontology (Overall et al., PLoS ONE, 2012) (Extended Data Fig. 8a). We found no consistent upregulation among representative neurogenesis-related genes in the Stim×10+2wks group:

Editorial Note: Supplementary Fig. 8 in this Peer Review File is reproduced with permission from BioRender, Created in BioRender. Murano, T. (2026) https://BioRender.com/p6ed2mm

Doublecortin was significantly increased, whereas PCNA and Pax6 were decreased, and NeuroD1, Nestin, and Calretinin showed no significant changes (Extended Data Fig. 8b). Co-immunostaining of Cyclin B and Doublecortin was technically infeasible because the antigen retrieval required for Cyclin B abolished Doublecortin signals. Instead, we performed single immunostaining for Doublecortin, which revealed a small increase in Doublecortin-positive cells (~3% of all granule cells; Extended Data Fig. 8c). In contrast, Cyclin B was expressed in approximately 90% of granule cells (Extended Data Fig. 11), indicating minimal overlap between Doublecortin-positive and Cyclin B-positive populations.

Together, these findings indicate that REPOPS primarily reactivates immature-like transcriptional programs within pre-existing mature neurons, rather than enhancing adult neurogenesis. Extended Data Figure 8 has been added to the revised manuscript.

Change:

[Original: ---]

[Revised: Extended Data Fig. 8] added

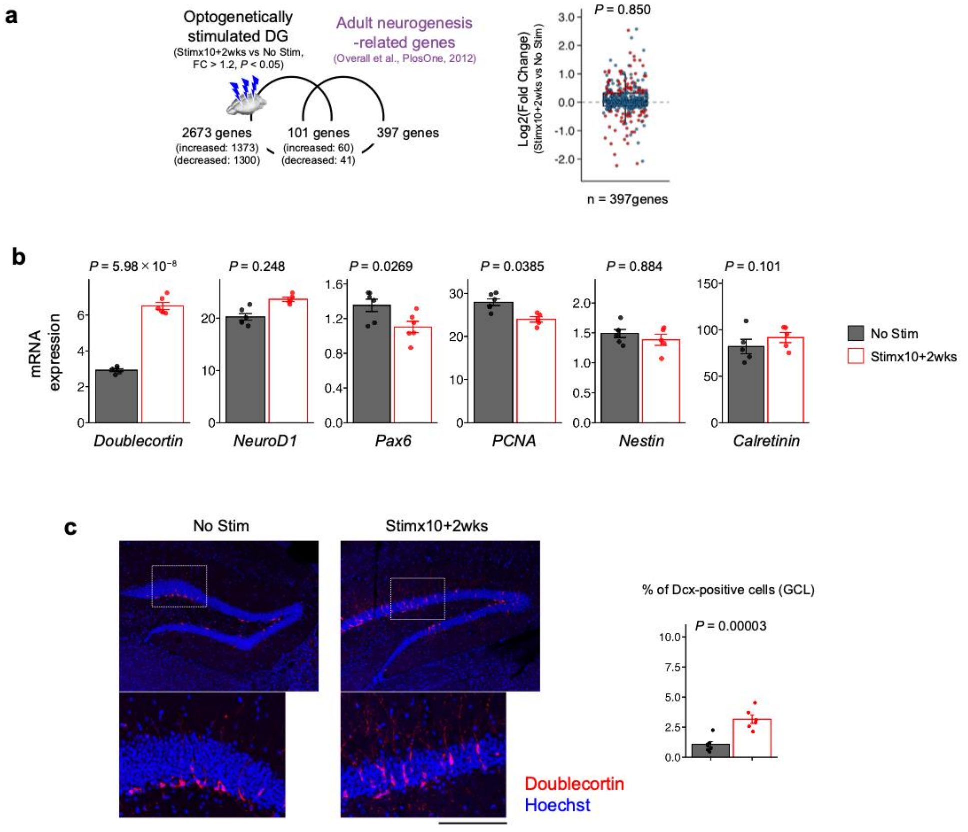

[for reference: Extended Data Fig. 11]

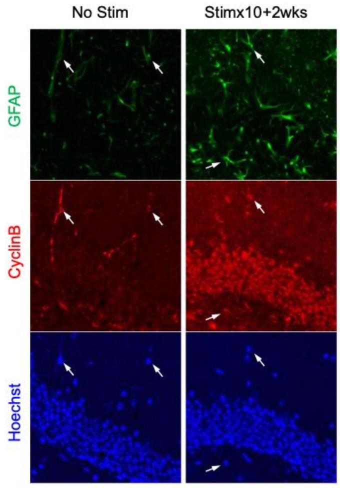

[Original: L239]

It was broadly expressed throughout the granule cell layer and was not restricted to the subgranular zone, where newborn neurons are abundant, indicating that the majority of cyclin B expression reflects induction in mature neurons, rather than enhanced neurogenesis.

[Revised: L277]

It was broadly expressed throughout the granule cell layer and was not restricted to the subgranular zone, where newborn neurons are abundant, indicating that the majority of cyclin B expression reflects induction in mature neurons, rather than enhanced neurogenesis (see also Extended Data Fig. 8).

[Original: ---]

[Revised: Extended Data Fig. 8, legend] added

Extended Data Figure 8. REPOPS-induced changes in neurogenesis-related genes.

a, Overlap between genes altered by REPOPS (Stim×10+2wks vs. No Stim) and neurogenesis-related genes (Overall et al., PLoS ONE, 2012)[4]. The mean $\log_2$(fold change) did not differ from zero (one-sample *t*-test, $t_{(391)} = -1.04$, $P = 0.85$). Red and blue dots represent genes with or without significant ($P < 0.05$) expression changes.

b, Expression of representative neurogenesis-related genes. Data are shown as mean ± s.e.m. (n = 6 mice per group).

c, Immunostaining of Doublecortin in the DG. Scale bar, 100 μm. Bar graphs show the percentage of Doublecortin-positive cells in the granule cell layer (GCL). Data are shown as mean ± s.e.m. Welch's *t*-test: $t_{(11)} = 5.19$, $P = 3.00 \times 10^{-4}$.

Doublecortin-positive cells were broadly distributed throughout the GCL rather than being confined to the subgranular zone, where adult-born neurons normally appear, implying re-expression of Doublecortin in mature neurons rather than newly generated cells. Doublecortin-positive cells accounted for 1.1% and 3.2% of all GCs in the No Stim and Stim×10+2wks groups, respectively, whereas approximately 90% of GCs expressed Cyclin B (Extended Data Fig. 11). These results suggest that the contribution of enhanced adult neurogenesis to REPOPS-induced changes is minimal, and instead, these changes primarily reflect partial reactivation of immature-like transcriptional programs within pre-existing mature neurons.

Major Comment 10

Additional cell-type markers: While NeuN, GFAP, and Iba1 staining was performed, the analysis would benefit from including inhibitory neuron markers (PV, SST) and excitatory subtypes (granule cells, mossy cells) to clarify which neuronal populations are additionally affected by REPOS treatment in mice.

Response:

We thank the reviewer for this valuable suggestion. In addition to NeuN, GFAP, and Iba1, we performed immunostaining for the interneuron marker parvalbumin (PV) (Extended Data Fig. 15d). We also reanalyzed the RNA-seq dataset focusing on established cell-type marker genes: *Prox1* and *Calb1* for granule cells, *Calb2* for mossy cells, *Pvalb* and *Sst* for interneurons, *Gfap* for astrocytes, and *Aif1* (*Iba1*) for microglia (Extended Data Fig. 15e). This analysis revealed that REPOPS induced significant transcriptional alterations in granule-cell markers (*Prox1*, *Calb1*) and in glial-cell markers (*Gfap*, *Aif1*), whereas interneuron-related genes (*Pvalb*, *Sst*) remained largely unchanged. These results indicate that REPOPS predominantly affects excitatory granule cells and glial populations, with minimal impact on inhibitory interneurons.

The new data are presented in Extended Data Fig. 15 and described in Supplementary Result 2.

Change:

[Original: ---]

[Revised: Extended Data Fig. 15] added

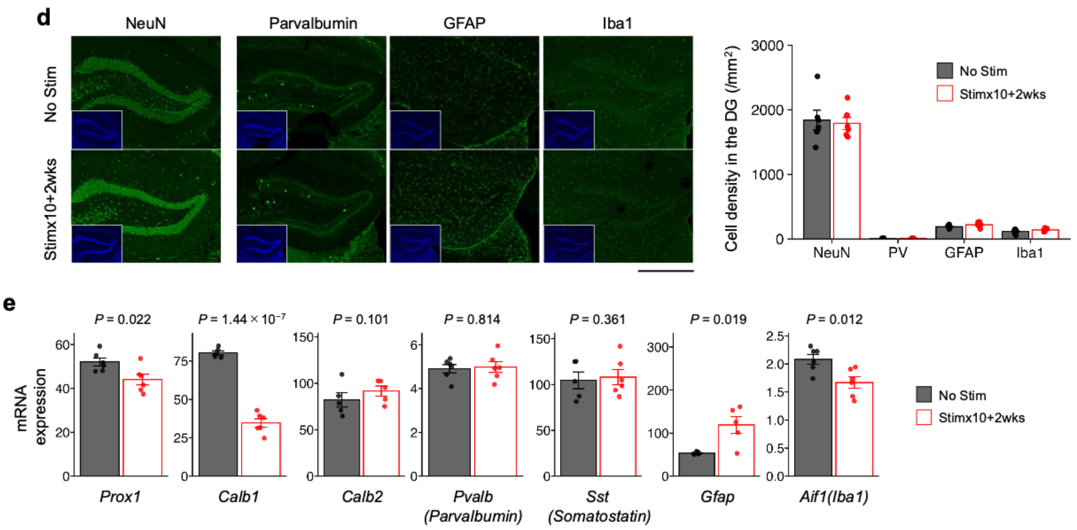

[Original: ---]

[Revised: Extended Data Fig. 15, legend] added

Extended Data Figure 15. REPOPS induces transcriptomic changes across multiple cell types, with predominant effects on granule cells.

d, Immunostaining for NeuN (excitatory neurons), PV (interneurons), GFAP (astrocytes), and Iba1 (microglia) in the DG. Scale bar, 500 μm. The bar graph shows the density of marker-positive cells.

e, mRNA expression of representative cell-type marker genes. Data are shown as mean ± s.e.m. (n = 6 mice per group). *P* values were calculated using Welch's *t*-test.

[Original: ---]

[Revised: Supplementary Result 2] added

Supplementary Result 2. REPOPS induces transcriptomic changes across multiple cell types, with predominant effects on granule cells (related to Extended Data Fig. 15).

To further validate these findings, we performed immunostaining for NeuN, parvalbumin (PV), GFAP, and Iba1 and quantified the density of marker-positive cells (Extended Data Fig. 15d). None of these markers showed significant differences in cell number after REPOPS. We also analyzed the mRNA expression of representative cell-type marker genes (Prox1 and Calb1 for granule cells, Calb2 for mossy cells, Pvalb and Sst for interneurons, Gfap for astrocytes, and Aif1 (Iba1) for microglia). REPOPS induced a significant reduction in Prox1, Calb1, and Iba1, and an increase in Gfap, whereas Calb2, Pvalb, and Sst remained unchanged (Extended Data

Fig. 15e). Given that granule cells overwhelmingly predominate within the DG cell composition (Extended Data Fig. 15a, d), these results support the conclusion that, although REPOPS affects multiple cell types, the majority of transcriptomic alterations observed in our bulk RNA-seq data are primarily driven by granule cells.

Minor Comment 1

Gene overlap analyses (Fig. 1D and 1F): Currently, the authors use Fisher's test on nominally significant genes ($p < 0.05$). I recommend a rank-rank hypergeometric overlap (RRHO) analysis, which is threshold-free and more powerful, allowing the entire ranked gene list to be compared. The Fisher's test could still be included, but I would also like to see the overlap after multiple-comparison correction for each dataset (e.g. FDR 5%).

Response:

We thank the reviewer for this valuable suggestion.

To address this, we reanalyzed the gene overlap using only differentially expressed genes that passed multiple-comparison correction (FDR < 0.05) (Extended Data Fig. 3). Significant overlaps were still observed between the mouse datasets (Stim×10+2wks and DG development). In contrast, in the dataset of ECT-treated patients, only a small number of DEGs (19 genes) were identified—likely due to high inter-individual variability—and no significant overlap was found with human hippocampal development. This limitation explains why nominal *P*-value thresholds were used in the main analysis.

We also performed a RRHO analysis to visualize genome-wide concordance between datasets. This RRHO analysis revealed strong concordance for genes ranked at both the upregulated and downregulated extremes of the lists, compared with the other rank combinations (up-/down-regulated or up-/down-regulated).

Both FDR-based overlap and RRHO results have been added to Extended Data Fig. 3.

Change:

[Original: ---]

[Revised: Extended Data Fig. 3] added

Editorial Note: Supplementary Fig. 3 in this Peer Review File is reproduced with permission from BioRender, Created in BioRender. Murano, T. (2026) https://BioRender.com/p6ed2mm

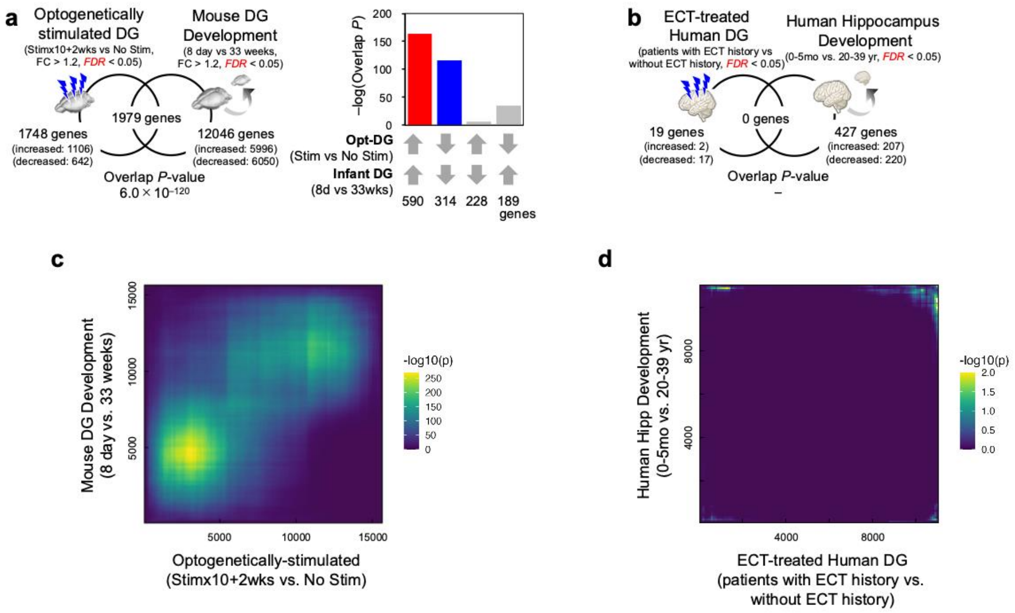

[Original: ---]

[Revised: Extended Data Fig. 3, legend] added

Extended Data Figure 3. Comparison of REPOPS/ECT-induced transcriptional changes with developmental datasets (related to Fig. 1d, f).

a, FDR-based gene overlap analysis. Left: Significant overlap between *mouse REPOPS* and *mouse DG development.* Right: No overlap between *human DG* after ECT treatment and *human hippocampal development.* Only 19 differentially expressed genes were identified in human DG after ECT treatment (FDR < 0.05), likely due to high inter-individual heterogeneity.

b, Rank-rank hypergeometric overlap (RRHO) analysis. RRHO heatmaps illustrate genome-wide concordance between datasets. The x- and y-axes indicate rank positions defining up- or down-regulated cutoffs in each dataset, and color intensity represents $-\log_{10}(P)$ values from Fisher's exact tests. Strong concordance was observed for genes at both up-regulated (lower left) and down-regulated (upper right) ends of the ranked lists, compared with the opposite combinations (upper left, lower right).

Minor Comment 2

Long-term Calbindin expression: Calbindin expression was tested up to 2 weeks poststimulation.

It would be valuable to extend the analysis to later time points (e.g., up to 1 month) to assess whether immature-like neuronal state is transient or persistent.

Response:

In response, we performed a time-course analysis of Calbindin expression from 1 day to 40 days after the last stimulation. The results demonstrated that the REPOPS-induced reduction in Calbindin expression persisted for at least 40 days, indicating that the immature-like neuronal state is sustained for more than one month.

Change:

[Original: ---]

[Revised: Extended Data Fig. 13] added

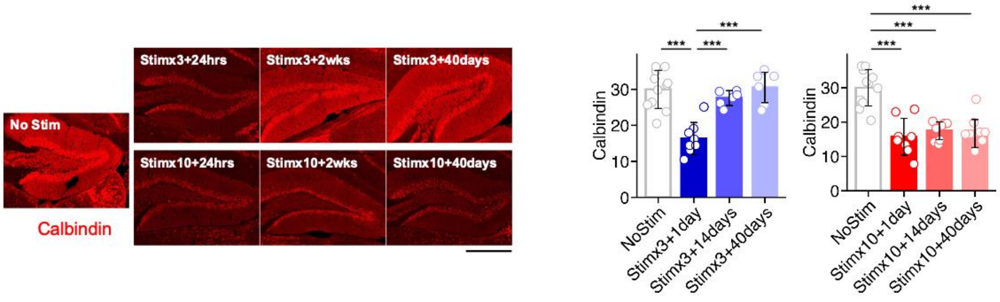

[Original: ---]

[Revised: Extended Data Fig. 13, legend]

Extended Data Figure 13. Long-term reduction of Calbindin after REPOPS (related to Fig. 7a,b).

Calbindin immunostaining in the dorsal DG. Scale bar, 500 µm. Bar graphs show Calbindin immunoreactivity (6–11 sections from 3 mice per group). Error bars represent mean ± s.e.m. One-way ANOVA: Stim×3, $F_{(3, 28)} = 18.4$, $P = 8.5 \times 10^{-7}$; Stim×10, $F_{(3, 35)} = 23.7$, $P = 1.5 \times 10^{-8}$. Multiple comparisons were performed using Tukey's post hoc test. ***$P < 0.001$.

Minor Comment 3

RNA-seq and ATAC-seq analysis: Important details are missing in the Methods section. Please specify the sample sizes, how genes/peaks were filtered, and whether covariates (e.g. RNA integrity number, sex) were included in differential analyses. In addition, the github code does not

include the RNA/ATAC-seq differential analysis code, which should be open source for reproducibility.

Response:

We thank the reviewer for this helpful comment. We have added detailed information regarding sample size, filtering criteria, covariates, and reproducibility to the Methods section.

(i) RNA-seq was performed with n = 6 mice per group, and ATAC-seq with n = 4 mice per group.

(ii) For RNA-seq, genes with FPKM > 0.1 across all samples were retained; for ATAC-seq, peaks overlapping ENCODE blacklist regions were removed.

(iii) All samples were derived from male mice with high RNA integrity numbers (RINs); therefore, sex and RIN were not included as covariates in the differential analyses.

(iv) Differential expression and accessibility analyses were performed using DESeq2, and the complete R scripts used for these analyses are publicly available on GitHub: https://github.com/tmurano/REPeatedOPtgeneticStimulation/blob/main/Fig1c_RNAseq/VolcanoPlot_RNAseq.R https://github.com/tmurano/REPeatedOPtgeneticStimulation/blob/main/Fig1hi_ATACseq/Volcano_ATACseq.R

Change:

[Original: L767]

RNA-Sequencing Library Construction, Sequencing, Mapping, and Data Analysis

The dentate gyrus was rapidly dissected from the adult mouse hippocampus under a dissection microscope as previously described[108]. Previous studies have shown that such preparations are highly enriched with mature dentate granule neurons (–90% NeuN$^+$ neurons[30,109]; see also Extended Data Fig. 7a). Total RNA from dissected tissue samples was extracted using NucleoSpin RNA (Macherey-Nagel, Duren, Germany), according to the manufacturer's instructions. The RNA quality was assessed with an Agilent 2200 TapeStation (Agilent Technologies, Santa Clara, CA, United States). Sequencing libraries were prepared using the TruSeq Stranded mRNA Sample Prep Kit (Illumina, San Diego, CA, USA) and sequenced as 100-bp paired-end reads on the Illumina HiSeq 2500. Sequenced data were mapped onto the Mus musculus genome (GRCm38/mm10) using TopHat (v2.0.14). Fragments per kilobase per million (FPKM) values were computed by Genedata Profiler Genome (v10.1.12). Genes with low expression (FPKM < 0.1) were excluded from the analysis to avoid an excessively high FDR due to low expression levels.

[Revised: 877]

RNA-Sequencing Library Construction, Sequencing, Mapping, and Data Analysis

RNA-seq was performed with six biological replicates per group (n = 6). All animals were male mice and exhibited high RNA integrity numbers (RINs); thus, sex and RIN were not included as covariates in the differential analysis. The dentate gyrus was rapidly dissected from the adult mouse hippocampus under a dissection microscope as previously described[117]. Previous studies have shown that such preparations are highly enriched with mature dentate granule neurons (–90% $NeuN^+$ neurons[27,118]; see also Extended Data Fig. 15a,d). Total RNA from dissected tissue samples was extracted using NucleoSpin RNA (Macherey-Nagel, Duren, Germany), according to the manufacturer's instructions. The RNA quality was assessed with an Agilent 2200 TapeStation (Agilent Technologies, Santa Clara, CA, United States). Sequencing libraries were prepared using the TruSeq Stranded mRNA Sample Prep Kit (Illumina, San Diego, CA, USA) and sequenced as 100-bp paired-end reads on the Illumina HiSeq 2500. Sequenced data were mapped onto the Mus musculus genome (GRCm38/mm10) using TopHat (v2.0.14). Fragments per kilobase per million (FPKM) values were computed by Genedata Profiler Genome (v10.1.12). Genes with low expression (FPKM < 0.1) were excluded from the analysis to avoid an excessively high FDR due to low expression levels. Differential expression analysis was performed using DESeq2. The R scripts used for these analyses are publicly available on GitHub (https://github.com/tmurano/REPeatedOPtgeneticStimulation).

[Original: L784]

ATAC-Sequencing Library Construction, Sequencing, Mapping, and Data Analysis

The dentate gyrus of adult mice was dissected in the same manner as that used for RNA-seq. The dentate gyrus was slowly frozen in a freezing box (Cat. No. 5100-0001; Mr. Frosty Freezing Container, Thermo Fisher Scientific). Flash-frozen tissue was sent to Active Motif to perform the ATAC-seq assay. The tissue was manually dissociated, isolated nuclei were quantified using a hemocytometer, and 100,000 nuclei were tagmented as previously described[110], with some modifications based on Corces et al. 2017[111] using the enzyme and buffer provided in the Nextera Library Prep Kit (Illumina). Tagmented DNA was purified using the MinElute PCR purification kit (Qiagen), amplified with 10 cycles of PCR, and purified using Agencourt AMPure SPRI beads (Beckman Coulter). The resulting material was quantified using the KAPA Library Quantification Kit for Illumina platforms (KAPA Biosystems) and sequenced using PE42 sequencing on a NextSeq 500 sequencer (Illumina). For the analysis of ATAC-seq data, we first aligned reads using the BWA algorithm (mem mode; default settings). Duplicate reads were removed, and only reads mapping as matched pairs and uniquely mapped reads (mapping quality >= 1) were used for further analysis. Alignments were extended in silico at

their 3’ends to a length of 200 bp and assigned to 32-nt bins along the genome. The resulting histograms (genomic "signal maps") were stored in the bigWig files. Peaks were identified using the MACS 2.1.0 algorithm at a cutoff of *P*-value 1.0×10^{-7}, without the control file, and with the –nomodel option. Peaks on the ENCODE blacklist of known false ChIP-Seq peaks were excluded. Signal maps and peak locations were used as input data for Active Motif’s proprietary analysis program, which creates Excel tables containing detailed information on sample comparisons, peak metrics, peak locations, and gene annotations. For differential analysis, reads were counted in all merged peak regions (using Subread) and the replicates for each condition were compared using DESeq2. IGV was used to visualize the raw intensities[112]. Statistical analyses were performed using an in-house R script, unless otherwise specified.

[Revised: L898]

ATAC-Sequencing Library Construction, Sequencing, Mapping, and Data Analysis

ATAC-seq was performed using n = 4 biological replicates per group. The dentate gyrus of adult mice was dissected in the same manner as that used for RNA-seq. The dentate gyrus was slowly frozen in a freezing box (Cat. No. 5100-0001; Mr. Frosty Freezing Container, Thermo Fisher Scientific). Flash-frozen tissue was sent to Active Motif to perform the ATAC-seq assay. The tissue was manually dissociated, isolated nuclei were quantified using a hemocytometer, and 100,000 nuclei were tagmented as previously described[119], with some modifications based on Corces et al. 2017[120] using the enzyme and buffer provided in the Nextera Library Prep Kit (Illumina). Tagmented DNA was purified using the MinElute PCR purification kit (Qiagen), amplified with 10 cycles of PCR, and purified using Agencourt AMPure SPRI beads (Beckman Coulter). The resulting material was quantified using the KAPA Library Quantification Kit for Illumina platforms (KAPA Biosystems) and sequenced using paired-end 42 bp sequencing on a NextSeq 500 sequencer (Illumina).

Reads were aligned to the mm10 genome using BWA (mem mode, default parameters). Duplicate reads were removed, and only reads mapping as matched pairs and uniquely mapped reads (mapping quality >= 1) were used for further analysis. Alignments were extended in silico at their 3’ends to a length of 200 bp and assigned to 32-nt bins along the genome. The resulting histograms (genomic "signal maps") were stored in the bigWig files. Peaks were identified using the MACS 2.1.0 algorithm at a cutoff of *P*-value 1.0×10^{-7}, without the control file, and with the –nomodel option. Peaks on the ENCODE blacklist of known false ChIP-Seq peaks were excluded. Intervals identified by MACS2 were merged by taking the union of overlapping peaks across all samples, ensuring consistent quantification among replicates. Read counts within merged regions were obtained using Subread, and differential accessibility was analyzed using DESeq2 (adjusted $P < 0.1$). Quality control metrics confirmed

robust data quality: FRiP scores ranged from 37 to 51%, exceeding the standard 10% benchmark. Aggregate accessibility profiles around promoters (TSS ± 5 kb) showed clear enrichment in all samples, confirming high signal-to-noise ratios (Extended Data Fig. 16). Significantly up- and down-regulated regions were exported as BED files, and 200-bp windows centered on each peak midpoint were analyzed for motif enrichment using HOMER. Both known and de novo motifs were identified after repeat masking to minimize false positives. Correlation analysis further confirmed high reproducibility across biological samples. Statistical analyses were performed using an in-house R script, unless otherwise specified.

Minor Comment 4

Cell death assay: Although TUNEL staining showed no apoptosis, RNA-seq identified enrichment of ‘positive regulation of cell death.’ This discrepancy suggests transcriptional activation of stress/apoptosis pathways without execution. The authors should check whether the DE genes are upstream regulators (stress response, pro-apoptotic signaling) or execution markers (caspases, BAX/BAK). Validation with Annexin V staining or caspase assays would clarify whether early apoptosis is occurring.

Response:

First, we would like to note that the enrichment of the GO term “positive regulation of cell death” in Extended Data Fig. 4 (original manuscript) indicates that these genes were differentially expressed, but not necessarily upregulated.

We examined REPOPS-induced changes in the expression of apoptosis-related genes. Among upstream regulators of apoptosis, *Gadd45a* was significantly upregulated by REPOPS in the Stim×10+2wks group (Extended Data Fig. 2a), whereas *Pmaip1*, *Trp53*, and *Ddit3* showed no significant changes. Among execution-phase genes, *Casp3*, *Bax*, and *Bak1* were unchanged, whereas *Casp7* was significantly downregulated (Extended Data Fig. 2a). Thus, although REPOPS induced mild activation of upstream stress-response genes, execution-phase genes were not upregulated, consistent with the absence of TUNEL-positive cells. These results are presented in Extended Data Fig. 2. Additionally, we summarized the expression profiles of all genes included in the GO term “positive regulation of cell death” in Supplementary Table 1.

Change:

[Original: Extended Data Fig. 1c] deleted

[Revised: Extended Data Fig. 2] added

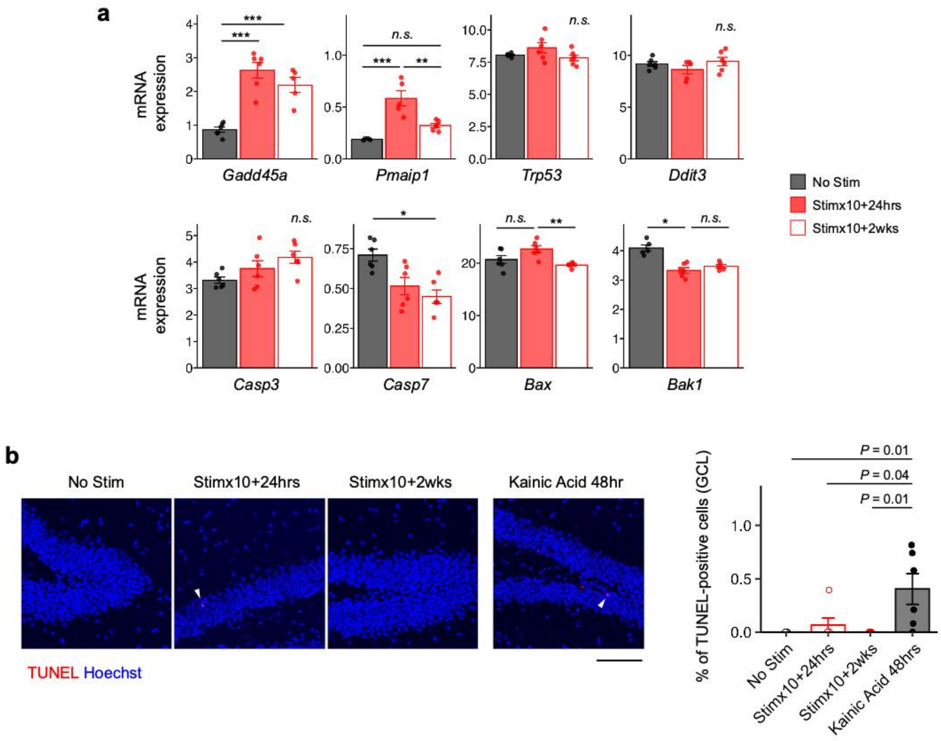

[Original: Extended Data Fig. 1c] deleted

[Revised: Extended Data Fig. 2, legend] added

Extended Data Figure 2. REPOPS for 10 days does not induce apoptosis.

a, Expression of apoptosis-related genes (upstream regulators: Gadd45a, Pmaip1, Trp53, and Ddit3; execution markers: Casp3, Casp7, Bax, and Bak1). Data are shown as mean ± s.e.m. (n = 6 mice per group). One-way ANOVA with Bonferroni correction; *$P < 0.05$, **$P < 0.01$, ***$P < 0.001$.

b, TUNEL staining (red) in the DG of mice treated with 10-day REPOPS or kainic acid (20 mg/kg, 48 hours after intraperitoneal injection). Arrowheads: TUNEL-positive cells. Scale bar, 500 µm. Bar graph shows the percentage of TUNEL-positive cells in the granule cell layer. Mean ± s.e.m. of 6 mice/group. One-way ANOVA; $F_{(3, 20)} = 6.00$, $P = 4.37 \times 10^{-3}$; Bonferroni correction was applied for multiple comparisons.

[Original: L110]

We employed a moderate stimulation protocol that did not cause cell death (Extended Data Fig. 1-b).

[Revised: L111] added

We employed a moderate stimulation protocol that did not cause cell death (Extended Data Fig. 1a, 1b, 2a, 2b; Supplementary Table 1).

[Original: ---]

[Revised: Supplementary Table 1] added

Supplementary Table 1. Genes altered by REPOPS that are annotated to the GO term "positive regulation of programmed cell death."

Minor Comment 5

Mouse age discrepancy: RNA-seq and ATAC-seq were performed in 3-4 month old mice, while other experiments used 6-12 month old mice. The rationale for this discrepancy should be clarified, as age strongly influences gene expression and behavior.

Response:

We appreciate the reviewer's insightful comment.

Due to practical constraints in animal availability at the time of each experiment, we were unable to perfectly align ages across all experimental cohorts. Importantly, each experiment included age-matched control groups.

We acknowledge that age differences could, in principle, influence cross-experimental comparisons; however, our previous study (Shoji et al., Neuropsychopharmacol. Rep, 2019) demonstrated minimal behavioral differences between 2- and 11-month-old mice, suggesting that the use of 3–4 month-old and 6–12 month-old cohorts in the present study is unlikely to substantially affect the conclusions. The Methods section has been revised accordingly, and the reference has been added.

Change:

[Original: L683]

Animals

… Mice were used for experiments at 3–4 months of age for RNA-seq and ATAC-seq experiments, and at 6–12 months for other experiments. …

[Revised: L767]

Animals

… For RNA-seq and ATAC-seq experiments, male mice aged 3–4 months were used. For other experiments, approximately equal numbers of male and female mice aged 6–12 months

were used. Age-matched control groups were included in each experiment. Our previous study[113] reported minimal behavioral differences between 2- and 11-month-old mice, indicating that the use of 3–4 month-old and 6–12 month-old mice in the present study is unlikely to confound the interpretation of the results. For the omics analyses, six mice per group were used for RNA-seq and four per group for ATAC-seq, which is consistent with standard practice in transcriptomic and epigenomic studies[27,28]. For behavioral experiments, 7–16 mice per group were used. Post hoc power analyses (α = 0.05) based on the observed effect sizes indicated that the achieved statistical powers (1−β) exceeded 0.80 in most tests, confirming that the sample sizes were sufficient to detect the main effects.

Minor Comment 6

Sample size inconsistencies: Please provide sample size for each experiment. It seems like different experiments use inconsistent group sizes (e.g. N=6 for RNA-seq, N=4 for ATAC-seq, N=10 for locomotor and FST/TST, N=7 for social interaction). The rationale should be explained, and power calculations would increase confidence.

Response:

We appreciate the reviewer's comment.

Differences in sample sizes across experiments were primarily due to resources and technical constraints. For the omics analyses, sample sizes were limited mainly by the financial cost of sequencing. RNA-seq (n = 6 per group) and ATAC-seq (n = 4 per group) followed widely accepted standards in similar studies (Su et al. Nat. Neurosci., 2017; Fernández et al. Nat. Neurosci., 2019), which typically use 3–4 biological replicates per condition. For behavioral experiments, group sizes (n = 7–10 per group) depended on the number of age-appropriate mice available at the time of each experiment.

As suggested by the reviewer, we performed post hoc power analyses (α = 0.05), which confirmed that the observed powers (1−β) based on the observed effect sizes exceeded 0.80 in most tests, indicating that the sample sizes provided adequate sensitivity to detect the main effects. These details have been added to the Methods section and Figure legends to ensure transparency.

Change:

[Original: L683]

Animals

… Mice were used for experiments at 3–4 months of age for RNA-seq and ATAC-seq experiments, and at 6–12 months for other experiments. …

[Revised: L767]

Animals

… For RNA-seq and ATAC-seq experiments, male mice aged 3–4 months were used. For other experiments, approximately equal numbers of male and female mice aged 6–12 months were used. Age-matched control groups were included in each experiment. Our previous study[113] reported minimal behavioral differences between 2- and 11-month-old mice, indicating that the use of 3–4 month-old and 6–12 month-old mice in the present study is unlikely to confound the interpretation of the results. For the omics analyses, six mice per group were used for RNA-seq and four per group for ATAC-seq, which is consistent with standard practice in transcriptomic and epigenomic studies[27,28]. For behavioral experiments, 7–16 mice per group were used. Post hoc power analyses ($\alpha = 0.05$) based on the observed effect sizes indicated that the achieved statistical powers ($1-\beta$) exceeded 0.80 in most tests, confirming that the sample sizes were sufficient to detect the main effects.

[Original: L513]

Figure 2. REPOPS induces anti-depressive behaviors

b, Total distance traveled in the first 15 min of the open field test from Day 1 to Day 24. Two-way repeated measures ANOVA; Stim type, $F_{(2, 14)} = 9.96$, $P = 2.0\times10^{-3}$; Day, $F_{(23, 322)} = 5.08$, $P = 4.63\times10^{-12}$. Stim type × Day, $F_{(46, 322)} = 2.58$, $P = 7.81\times10^{-7}$.

c, 24-hour locomotor activity in the home cage. Daily average distance traveled is shown (n = 10, 8, and 10 mice for No Stim, Stim×3, and Stim×10 groups, respectively). Data missing on Day 28, 29, 37 and 47 due to computer system error. $P < 0.05$ on Day 7–8, 10–16, 20, 22–23, 25–30, 33–36 (Stim×10 vs. No Stim).

d, Immobility (%) in tail suspension test. Values are the Mean ± s.e.m. Two-way repeated-measures ANOVA results shown in panel. n = 10, 9, and 10 mice. Box plot summarizes average immobility over 10 min. One-way ANOVA with Bonferroni correction; *$P < 0.05$, **$P < 0.01$, ***$P < 0.001$.

e, Same as **d**, for immobility (%) in the forced swim test.

[Revised: L595]

Figure 3. REPOPS induces anti-depressive behaviors.

b, Total distance traveled in the first 15 min of the open field test from Day 1 to Day 24. Two-way repeated measures ANOVA; Stim type, $F_{(2, 14)} = 9.96$, $P = 2.0\times10^{-3}$; Day, $F_{(23, 322)} = 5.08$, $P = 4.63\times10^{-12}$. Stim type × Day, $F_{(46, 322)} = 2.58$, $P = 7.81\times10^{-7}$. Observed power ($1-\beta$) for the main effect of stimulation group was 0.99 ($\alpha = 0.05$).

c, 24-hour locomotor activity in the home cage. Daily average distance traveled is shown (n = 10, 8, and 10 mice for No Stim, Stim×3, and Stim×10 groups, respectively). Data missing on Day 28, 29, 37 and 47 due to computer system error. $P < 0.05$ on Day 7–8, 10–16, 20, 22–23, 25–30, 33–36 (Stim×10 vs. No Stim). Two-way repeated-measures ANOVA; stimulation group, $F_{(2, 25)} = 4.94$, $P = 0.016$; Day, $F_{(46, 1150)} = 3.32$, $P = 2.54\times10^{-12}$; interaction, $F_{(92, 1150)} = 1.02$, $P = 0.42$. Observed power (1−β) = 0.78 (α = 0.05).

d, Immobility (%) in tail suspension test. Values are the Mean ± s.e.m. Two-way repeated-measures ANOVA results shown in panel. n = 10, 9, and 10 mice. Stimulation group: $F_{(2, 26)} = 6.34$, $P = 0.006$; Timebin: $F_{(5.84, 151.83)} = 18.75$, $P < 0.001$; interaction: $F_{(11.68, 151.83)} = 1.45$, $P = 0.15$. Observed powers (1−β) were 0.88, >0.99, and 0.50, respectively. Box plot summarizes average immobility over 10 min. One-way ANOVA with Bonferroni correction; *$P < 0.05$, **$P < 0.01$, ***$P < 0.001$.

e, Same as **d**, for immobility (%) in the forced swim test. Stimulation group: $F_{(2, 26)} = 4.28$, $P = 0.025$; Timebin: $F_{(3.61, 93.81)} = 143.73$, $P < 0.001$; interaction: $F_{(7.22, 93.81)} = 0.74$, $P = 0.65$. Observed powers (1−β) were 0.82, >0.99, and 0.30, respectively.

[Original: Extended Data Fig. 3b]

Extended Data Figure 3. Impaired memory and social behavior following repeated optogenetic stimulation

b, Social interaction test. (i) Time spent in each chamber (empty, center, stranger mouse) (n = 7 mice/group). One-way ANOVA: No Stim, $F_{(2,18)} = 12.7$, $P = 3.63 \times 10^{-4}$; Stim×3, $F_{(2,18)} = 30.42$, $P = 1.69 \times 10^{-6}$; Stim×10, $F_{(2,18)} = 27.33$, $P = 3.51 \times 10^{-6}$. P-values in the graphs were Bonferroni corrected for multiple comparisons. (ii) Time spent around the empty cage vs. the cage with a stranger mouse. Student t-test: No Stim, $t_{(12)} = 2.27$, $P = 0.043$; Stim×3, $t_{(12)} = 2.54$, $P = 0.026$; Stim×10, $t_{(12)} = 0.17$, $P = 0.87$. (iii) Preference index, calculated as time spent near the stranger mouse divided by total time spent near both cages. One-way ANOVA, $F_{(2,17)} = 4.18$, $P = 0.0333$. P-values were Bonferroni-corrected for multiple comparisons.

[Revised: Extended Data Fig. 6b]

Extended Data Figure 6. Impaired memory and social behavior following repeated optogenetic stimulation.

b, Social interaction test. (i) Locomotor activity (n = 7 mice per group). One-way ANOVA: No Stim, $F_{(2, 18)} = 3.172$, $P = 0.0661$. (ii) Time spent in each chamber (empty, center, stranger mouse) (n = 7 mice/group). One-way ANOVA: No Stim, $F_{(2, 18)} = 12.7$, $P = 3.63 \times 10^{-4}$; Stim×3, $F_{(2, 18)} = 30.42$, $P = 1.69 \times 10^{-6}$; Stim×10, $F_{(2, 18)} = 27.33$, $P = 3.51 \times 10^{-6}$. P-values in the graphs were Bonferroni corrected for multiple comparisons. Observed powers (1−β) were 0.96 (No

Stim), 0.99 (Stim×3), and 0.99 (Stim×10). (iii) Time spent around the empty cage vs. the cage with a stranger mouse. Student *t*-test: No Stim, $t_{(12)} = 2.27$, $P = 0.043$; Stim×3, $t_{(12)} = 2.54$, $P = 0.026$; Stim×10, $t_{(12)} = 0.17$, $P = 0.87$. Observed powers (1−β) were 0.78 (No Stim), 0.85 (Stim×3), and 0.06 (Stim×10). (iv) Preference index, calculated as time spent near the stranger mouse divided by total time spent near both cages. One-way ANOVA, $F_{(2, 17)} = 4.18$, $P = 0.0333$. *P*-values were Bonferroni-corrected for multiple comparisons.

Minor Comment 7

Parallels with ECT: Typically, ECT does not induce chronic hyperlocomotion in humans. The persistent hyperactivity seen in mice raises concerns about how well REPOPS models therapeutic effects of ECT. Please explain this in Discussion section

Response:

We appreciate the reviewer's insightful comment and agree that chronic hyperlocomotion is not a typical clinical consequence of ECT in humans. However, several clinical studies indicate that effective antidepressant treatment is often accompanied by increased spontaneous motor activity. Actigraphic measurements revealed that patients with depression exhibited elevated activity following antidepressant or ECT treatment (Volkers et al., *Eur. Neuropsychopharmacol.*, 2002; Winkler et al., *J. Psychiatr. Res.*, 2014), and motor activity levels were shown to correlate with depression severity (Spulber et al., *Transl. Psychiatry*, 2022). Collectively, these findings suggest that enhanced motor activity is a common physiological correlate of effective antidepressant treatment, including ECT. Therefore, the persistent hyperlocomotor phenotype in REPOPS mice likely represents a biologically relevant correlate of the behavioral activation observed in patients receiving ECT. We have clarified this interpretation in the Discussion section.

Change:

[Original: L179]

These results indicate that repeated neuronal activation causes a significant increase in baseline locomotor activity, which persists for more than one month.

[Revised: L213]

These results indicate that repeated neuronal activation causes a significant increase in baseline locomotor activity, which persists for more than one month. Notably, in humans, effective antidepressant treatment is accompanied by increased spontaneous motor activity (Volkers et al., Eur. Neuropsychopharmacol., 2002; Winkler et al., J. Psychiatr. Res., 2014;

Spulber et al., Transl. Psychiatry, 2022)[51–53], supporting the relevance of the enhanced activity observed in our model.

Minor Comment 8

3-day and 10-day REPOPS: The rationale for these timeframes is not fully justified. Why 3 and 10? Do these correspond to clinical ECT regimens (e.g., acute vs maintenance treatments)? Please clarify why and how these timepoints were selected.

Response:

We thank the reviewer for raising this point. As noted in the Introduction (L73) and Discussion (L350), repeated ECT sessions (typically 6–12) are required to achieve sustained therapeutic efficacy. Accordingly, we adopted 3-day and 10-day REPOPS protocols to model a subthreshold (insufficient) regimen and a long-lasting (sufficient) regimen, respectively. This design allowed us to examine how the cumulative number of stimulations determines the transition from transient to persistent molecular and behavioral changes. We have clarified this rationale in the Results section.

Change:

[Original: L113]

REPOPS was performed either three or ten times consecutively (Fig. 1a). The expression of calbindin (CB), a maturation marker of GCs, was reduced …

[Revised: L114]

REPOPS was performed either three or ten times consecutively (Fig. 1a). These regimens were selected to contrast a minimal (3-day) protocol with an extended (10-day) regimen, approximating the 6–12 sessions typically required to achieve clinical efficacy in ECT. This design enabled us to assess how the cumulative number of stimulations influences the emergence of stable molecular and behavioral effects. The expression of calbindin (CB), a maturation marker of GCs, was reduced …

Minor Comment 9

Knockout efficiency: The authors state cyclin B expression was “effectively eliminated,” but I was not able to find quantitative data to support this (e.g., reduction in RNA or protein levels). Was knockout uniform across the DG or mosaic? Did authors validate specificity with sequencing or off-target prediction methods?

Response:

We thank the reviewer for this helpful comment. Data demonstrating the effective and uniform elimination of REPOPS-induced Cyclin B expression are presented in Extended Data Fig. 7b of the original manuscript. We apologize that the original figure title did not clearly indicate that this panel represented validation of knockout efficiency, which may have caused confusion. To improve clarity, we have now separated this panel as Extended Data Fig. 11 and revised its title accordingly.

In addition, sgRNA sequences were designed using CRISPRdirect (https://crispr.dbcls.jp/) to minimize predicted off-target effects. These details have been added to the Methods section.

Change:

[Original: Extended Data Fig. 7b] deleted

[Revised: Extended Data Fig. 11] added

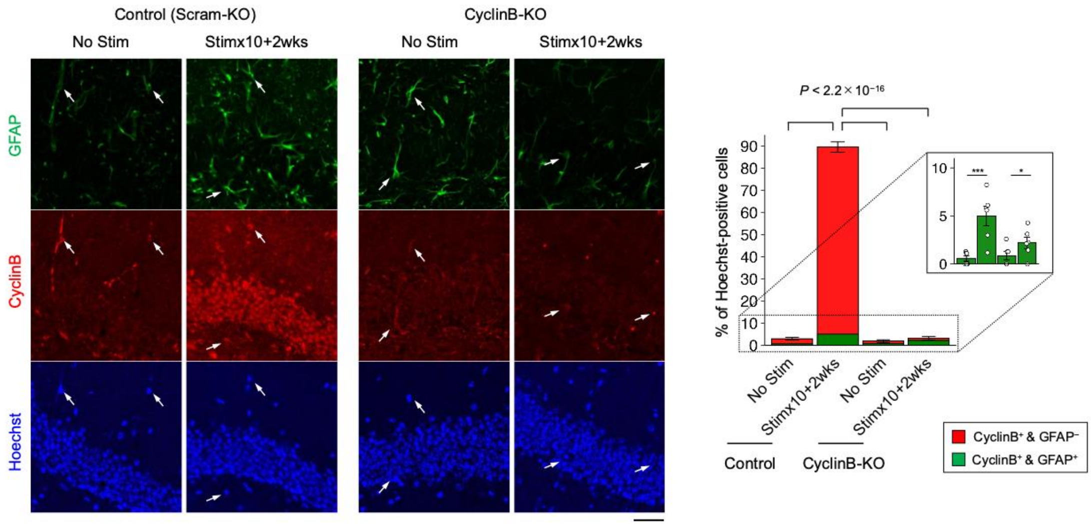

[Original: ---]

[Revised: Extended Data Fig. 11, legend] added

Extended Data Figure 11. Efficient knockout of REPOPS-induced Cyclin B expression. Confocal images showing co-staining of GFAP and Cyclin B. Arrows indicate GFAP^{+}/Cyclin B^{+} astrocytes. Scale bar, 20 μm. Bar graphs show the percentage of Cyclin B^{+} cells among Hoechst^{+} cells (left), and GFAP^{+}/Cyclin B^{+} cells (right). One-way ANOVA; $F_{(3, 20)}$ = 1092 and

$F_{(3, 20)} = 24.4$, $P < 2.0 \times 10^{-16}$ and $P = 3.45 \times 10^{-4}$. Bonferroni correction was applied for multiple comparisons. *$P < 0.05$, **$P < 0.01$, ***$P < 0.001$.

Cyclin B+ cells were markedly increased in the control group following REPOPS but were effectively eliminated in the CyclinB-KO group. The reduction was observed uniformly across the dentate gyrus, confirming efficient and spatially consistent knockout of Cyclin B.

[Original: L713]

Stereotactic injection of AAV9

For the genome editing experiment (Fig. 5), a mixture of adeno-associated virus (AAV) carrying Cas9 and sgRNA was injected into the DG immediately before optic fiber implantation. For dominant-negative inhibition of ΔFosB experiment (Extended Data Fig. 9), we injected an AAV expressing ΔJunD (a kind gift from A.J. Robison) was injected. Anesthetization and craniotomy were performed in the same way as described above. A glass-capillary pipette (25 μm inner-diameter tip) filled with the virus suspended in mineral oil was inserted into the target site (2.0 mm posterior to bregma (AP), 1.5 mm lateral to midline (ML)) through the surgical drill on the skull. A 200 nL of 1:3 AAV mixture of AAV9-U6-sgRNA (*Ccnb1*-*Ccnb2* or Scramble)-hSyn1-mCherry (2.0×10^{13} vector genomes vg/ml) and AAV9-pMecp2-HA-SpCas9 (2.0×10^{13} vg/ml) was injected at 200 nL/min using a microinjection pump (Nanoliter 2010, WPI, FL) at three depths (1.5 mm, 2.0 mm, and 2.5 mm). After each injection, a glass pipette was held in place for 1–2 min before retraction to prevent leakage. Shortly after virus injection, the optic fiber was embedded in the same site using the procedure described above.

[Revised: L812]

Stereotactic injection of AAV9

Anesthetization and craniotomy were performed as described above. A glass-capillary pipette (25 μm inner-diameter tip) filled with virus suspended in mineral oil was inserted into the target site (2.0 mm posterior to bregma (AP), 1.5 mm lateral to midline (ML)) through the surgical drill on the skull. For the genome-editing experiment (Fig. 6), a mixture of adeno-associated viruses (AAVs) carrying Cas9 and sgRNA was injected into the DG. A 200 nL volume of a 1:3 mixture of AAV9-U6-sgRNA (*Ccnb1*-*Ccnb2* or Scramble)-hSyn1-mCherry (2.0×10^{13} vector genomes vg/mL) and AAV9-pMecp2-HA-SpCas9 (2.0×10^{13} vg/mL) was injected at 200 nL/min using a microinjection pump (Nanoliter 2010, WPI, FL) at three depths (1.5, 2.0, and 2.5 mm). After each injection, the glass pipette was held in place for 1–2 min before retraction to prevent leakage. The optic fiber was implanted in the same site immediately after injection. For the ΔFosB inhibition experiment (Extended Data Fig. 14), an AAV expressing ΔJunD (a kind gift from A.J. Robison) was injected using the same procedure.

For SpCas9 target selection and sgRNA design, 20-nt target sequences preceding a 5’-NGG PAM motif were selected. To minimize off-target effects, the CRISPR design tool (https://crispr.dbcls.jp/) was used. sgRNAs targeting *Ccnb1*, *Ccnb2* and *Scramble* control were designed as follows: *Ccnb1*: 5'-GCTAC AGCTG GTCGG TGTAA CGG-3', *Ccnb2*: 5'-GGCGC TGCTC CGACG CCCGA CGG-3', *Scramble*: 5'-GCACT ACCAG AGCTA ACTCA CGG -3'. The backbone plasmid for sgRNA (PX552; pAAV-U6sgRNA(SapI)_hSyn-GFP-KASH-bGH) was obtained from Addgene. *Ccnb*-sgRNA (tandemly connecting *Ccnb1* and *Ccnb2*; Fig. 6b) and *Scramble*-sgRNA nucleotides were synthesized and cloned into AAV backbone plasmids (Vigene Biosciences, USA). To avoid fluorescent overlap with ChR2-EYFP, the GFP cassette was replaced with mCherry (Fig. 6b). The plasmid for SpCas9 (PX551; pAAV-pMecp2-SpCas9-spA) was also obtained from Addgene. Concentrated AAV vectors were produced from these plasmids with support from the Gunma University Initiative for Advanced Research Viral Vector Core.

Minor Comment 10

Specificity of detection: Since ΔFosB is a truncated isoform, the authors must clarify whether their antibody or probe distinguishes ΔFosB from full-length FosB. Without isoform specific validation, claims about ΔFosB may be confounded.

Response:

We thank the reviewer for this important comment.

We used a ΔFosB-specific rabbit monoclonal antibody (Cell Signaling Technology, #14695, clone D3S8R). According to the manufacturer’s validation data (https://www.cellsignal.com/products/primary-antibodies/delta-fosb-d3s8r-rabbit-mab/14695), this antibody specifically recognizes ΔFosB without cross-reactivity to full-length FosB. Consistent with this, previous studies (e.g., Stephens et al., Front. Neurosci., 2020, Supplementary Fig. 1) have confirmed that this antibody detects ΔFosB (35–37 kDa) but not the full-length FosB (46–48 kDa). Thus, the antibody’s isoform specificity has been thoroughly validated by both the manufacturer and independent researchers. We have clarified this point and added the relevant citation to the Methods section.

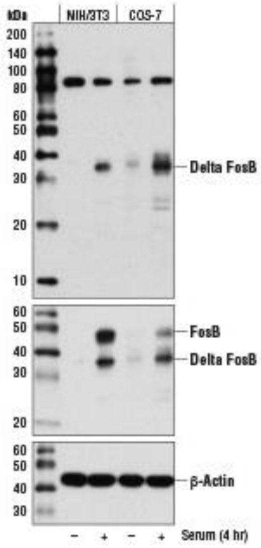

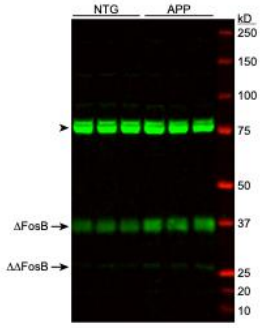

Supplemental Figure S1. Anti-ΔFosB antibody D3S8R (Cell Signaling, 1:3000) was used to probe a Western blot of hippocampal lysates (40 micrograms protein/lane) from NTG and APP mice. Arrows denote bands for Δ FosB and possible Δ2ΔFosB, levels of which appear very low or negligible in hippocampus as we previously found (Corbett et al., 2017). Arrowhead denotes possible nonspecific band around 80-85kD. Note that the intensity of the 80-85 kD band does not differ between genotypes. The ΔFosB band at 37 kD is increased in intensity in APP mice relative to NTG littermate controls, as expected, suggesting that genotype or treatment specific differences in target genes pulled down by this antibody are likely due to ΔFosB binding.

Left panel: from the Cell Signaling Technology validation data. The upper image shows a Western blot stained with the ΔFosB antibody (#14695, clone D3S8R). A clear ΔFosB band is observed at 35–37 kDa, whereas the 46–48 kDa full-length FosB band is completely absent. A nonspecific band appears at approximately 80–85 kDa. The middle image shows staining with the FosB rabbit polyclonal antibody (#2263), in which both ΔFosB and full-length FosB are detected.

Right panel: from Stephens et al., Front. Neurosci., 2020, Supplementary Figure 1. Western blot of hippocampal lysates stained with the ΔFosB antibody (#14695, clone D3S8R). A distinct ΔFosB band is detected at 35–37 kDa, while no signal corresponding to the 46–48 kDa full-length FosB is observed.

Change:

[Original: L821]

The sections were stained with the following antibodies: Calbindin (1:1000, rabbit, polyclonal, Synaptic Systems Cat# 214 002, RRID:AB_2068199), Cyclin B (1:500, mouse, monoclonal, Thermo Fisher Scientific Cat# MA1-155, RRID:AB_2536863), phospho-Histone H3 (1:500, rabbit, polyclonal, Millipore Cat# 06-570, RRID:AB_310177), HA-tag (1:500, rabbit, polyclonal, Cell Signaling Technology Cat# 3724, RRID:AB_1549585), ΔFosB (1:1000, rabbit, monoclonal, Cell Signaling Technology Cat# 14695, clone D3S8R, RRID:AB_2798577)…

[Revised: L941]

The sections were stained with the following antibodies: Calbindin (1:1000, rabbit, polyclonal, Synaptic Systems Cat# 214 002, RRID:AB_2068199), Cyclin B (1:500, mouse, monoclonal,

Thermo Fisher Scientific Cat# MA1-155, RRID:AB_2536863), phospho-Histone H3 (1:500, rabbit, polyclonal, Millipore Cat# 06-570, RRID:AB_310177), HA-tag (1:500, rabbit, polyclonal, Cell Signaling Technology Cat# 3724, RRID:AB_1549585), ΔFosB (1:1000, rabbit, monoclonal, Cell Signaling Technology Cat# 14695, clone D3S8R, RRID:AB_2798577)…

The ΔFosB antibody (Cell Signaling Technology, Cat# 14695, clone D3S8R, RRID:AB_2798577) specifically recognizes the ΔFosB isoform (35–37 kDa) without cross-reactivity to the full-length FosB (46–48 kDa), as verified by both the manufacturer and an independent study (Stephens et al., Front. Neurosci., 2020)[121].

Minor Comment 11

Lack of transparency in ATAC-seq analysis: Authors mention that the signal maps and peak locations were used as input data for Active Motif's proprietary analysis program. While I understand that the authors used Active Motif's proprietary program, it is important for transparency and reproducibility to include more information on what algorithms and parameters were used for peak calling and gene annotation.

Response:

Peak calling was performed using MACS2 (v2.1.0) with a cutoff of $P < 1e\text{-}7$, the –nomodel option, and without a control file. Peaks overlapping with regions defined in the ENCODE blacklist were removed. Each interval and merged region was then assigned to the nearest gene if located within ±10 kb of the transcription start site (TSS). These details have now been added to the Methods section.

Change:

[Original: L784]

ATAC-Sequencing Library Construction, Sequencing, Mapping, and Data Analysis

The dentate gyrus of adult mice was dissected in the same manner as that used for RNA-seq. The dentate gyrus was slowly frozen in a freezing box (Cat. No. 5100-0001; Mr. Frosty Freezing Container, Thermo Fisher Scientific). Flash-frozen tissue was sent to Active Motif to perform the ATAC-seq assay. The tissue was manually dissociated, isolated nuclei were quantified using a hemocytometer, and 100,000 nuclei were tagmented as previously described[110], with some modifications based on Corces et al. 2017[111] using the enzyme and buffer provided in the Nextera Library Prep Kit (Illumina). Tagmented DNA was purified using the MinElute PCR purification kit (Qiagen), amplified with 10 cycles of PCR, and purified using Agencourt AMPure SPRI beads (Beckman Coulter). The resulting material was quantified using the KAPA Library

Quantification Kit for Illumina platforms (KAPA Biosystems) and sequenced using PE42 sequencing on a NextSeq 500 sequencer (Illumina). For the analysis of ATAC-seq data, we first aligned reads using the BWA algorithm (mem mode; default settings). Duplicate reads were removed, and only reads mapping as matched pairs and uniquely mapped reads (mapping quality >= 1) were used for further analysis. Alignments were extended in silico at their 3'ends to a length of 200 bp and assigned to 32-nt bins along the genome. The resulting histograms (genomic "signal maps") were stored in the bigWig files. Peaks were identified using the MACS 2.1.0 algorithm at a cutoff of P-value 1.0×10^{-7}, without the control file, and with the –nomodel option. Peaks on the ENCODE blacklist of known false ChIP-Seq peaks were excluded. Signal maps and peak locations were used as input data for Active Motif's proprietary analysis program, which creates Excel tables containing detailed information on sample comparisons, peak metrics, peak locations, and gene annotations. For differential analysis, reads were counted in all merged peak regions (using Subread) and the replicates for each condition were compared using DESeq2. IGV was used to visualize the raw intensities[112]. Statistical analyses were performed using an in-house R script, unless otherwise specified.

[Revised: L898]

ATAC-Sequencing Library Construction, Sequencing, Mapping, and Data Analysis

ATAC-seq was performed using n = 4 biological replicates per group. The dentate gyrus of adult mice was dissected in the same manner as that used for RNA-seq. The dentate gyrus was slowly frozen in a freezing box (Cat. No. 5100-0001; Mr. Frosty Freezing Container, Thermo Fisher Scientific). Flash-frozen tissue was sent to Active Motif to perform the ATAC-seq assay. The tissue was manually dissociated, isolated nuclei were quantified using a hemocytometer, and 100,000 nuclei were tagmented as previously described[119], with some modifications based on Corces et al. 2017[120] using the enzyme and buffer provided in the Nextera Library Prep Kit (Illumina). Tagmented DNA was purified using the MinElute PCR purification kit (Qiagen), amplified with 10 cycles of PCR, and purified using Agencourt AMPure SPRI beads (Beckman Coulter). The resulting material was quantified using the KAPA Library Quantification Kit for Illumina platforms (KAPA Biosystems) and sequenced using paired-end 42 bp sequencing on a NextSeq 500 sequencer (Illumina).

Reads were aligned to the mm10 genome using BWA (mem mode, default parameters). Duplicate reads were removed, and only reads mapping as matched pairs and uniquely mapped reads (mapping quality >= 1) were used for further analysis. Alignments were extended in silico at their 3'ends to a length of 200 bp and assigned to 32-nt bins along the genome. The resulting histograms (genomic "signal maps") were stored in the bigWig files. Peaks were identified using the MACS 2.1.0 algorithm at a cutoff of P-value 1.0×10^{-7}, without

the control file, and with the –nomodel option. Peaks on the ENCODE blacklist of known false ChIP-Seq peaks were excluded. Intervals identified by MACS2 were merged by taking the union of overlapping peaks across all samples, ensuring consistent quantification among replicates. Read counts within merged regions were obtained using Subread, and differential accessibility was analyzed using DESeq2 (adjusted $P < 0.1$). Quality control metrics confirmed robust data quality: FRiP scores ranged from 37 to 51%, exceeding the standard 10% benchmark. Aggregate accessibility profiles around promoters (TSS ± 5 kb) showed clear enrichment in all samples, confirming high signal-to-noise ratios (Extended Data Fig. 16). Significantly up- and down-regulated regions were exported as BED files, and 200-bp windows centered on each peak midpoint were analyzed for motif enrichment using HOMER. Both known and de novo motifs were identified after repeat masking to minimize false positives. Correlation analysis further confirmed high reproducibility across biological samples. Statistical analyses were performed using an in-house R script, unless otherwise specified.

%%%%% end of file %%%%%

Reviewer #2

Comment 1

Although the behavioral effects of REPOPS in the NSFT are convincing and nicely replicated in a depression model, no effect is seen in the SPT, and the FST and TST results remain confounded by hyperlocomotion. I would suggest moving the NSFT to Figure 3 in place of the FST or TST as this provides the clearest evidence for a therapeutically-relevant behavioral effect. The authors may consider addressing whether this model impacts specific depression-related behavioral phenotypes and not others, and whether these differential effects are related to the regional specificity of their neuronal stimulation.

Response:

We thank the reviewer for this constructive suggestion. We have prioritized the NSFT as the main behavioral evidence and moved NSFT to Fig. 3e**,** while relocating the FST to Extended Data Fig. 5c**.** This reorganization emphasizes the assay that provides the clearest evidence for an affective behavioral change with minimal confounding by REPOPS-induced hyperlocomotion. We also address the reviewer's point by discussing that dentate gyrus–targeted stimulation may preferentially affect specific behavioral domains rather than producing uniform effects across all assays, and note this as a potential consequence of regional/circuit specificity.

Change:

[Original (1st revision): Fig. 3 and Extended Data Fig. 5]

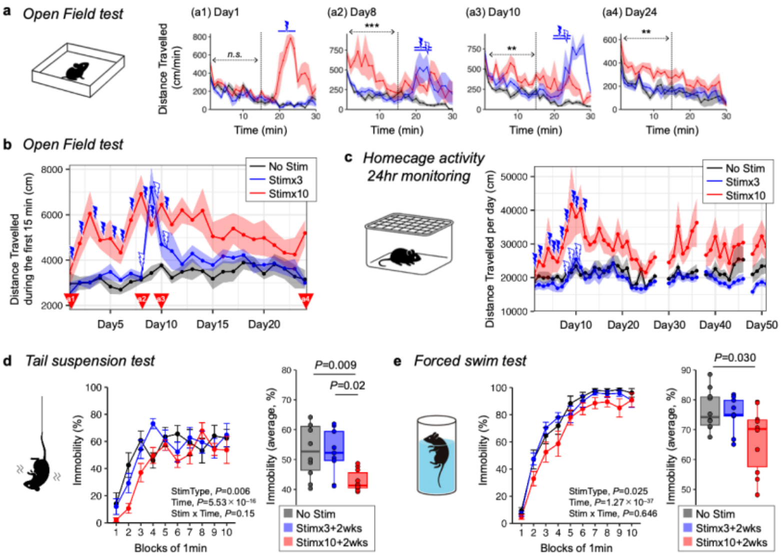

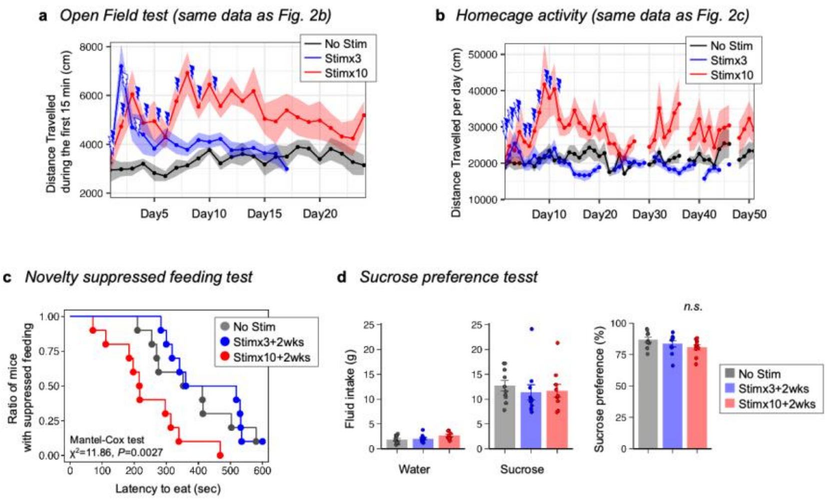

[Revised (2nd revision): Fig. 3 and Extended Data Fig. 5] Revised

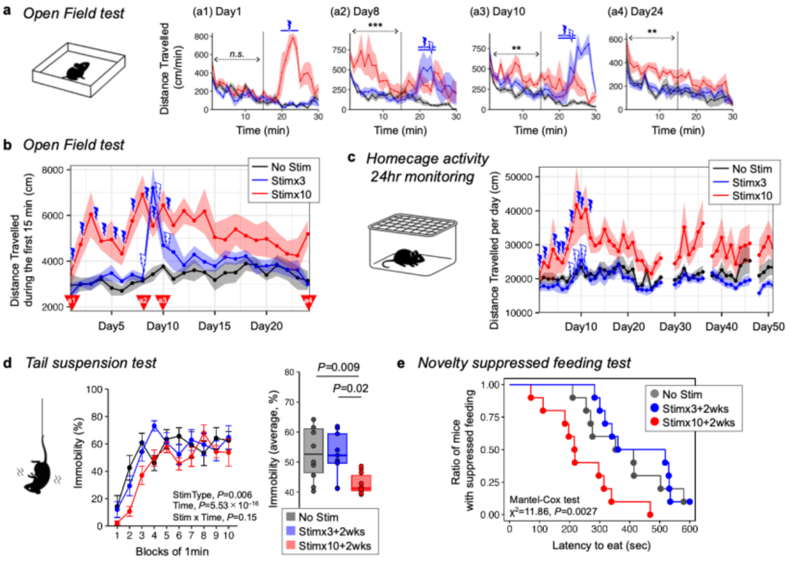

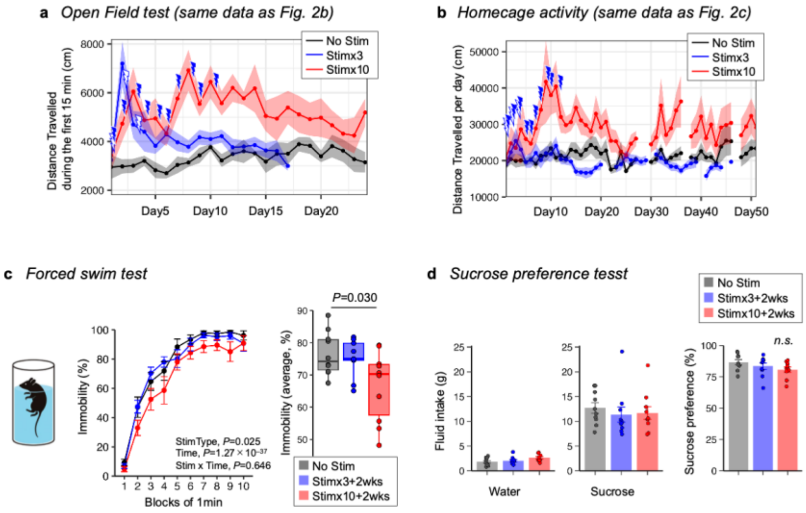

[Original (1st revision): Main Text, L619]

Figure 3. REPOPS induces anti-depressive behaviors.

e, Same as **d**, for immobility (%) in the forced swim test. Stimulation group: $F_{(2, 26)} = 4.28$, $P = 0.025$; Timebin: $F_{(3.61, 93.81)} = 143.73$, $P < 0.001$; interaction: $F_{(7.22, 93.81)} = 0.74$, $P = 0.65$. Observed powers (1−β) were 0.82, >0.99, and 0.30, respectively.

[Revised (2nd revision): Main Text, L619] Revised

Figure 3. REPOPS induces anti-depressive behaviors.

e, Novelty-suppressed feeding test. Latency to feed per mouse shown as Kaplan–Meier survival curves depicting the probability of not eating over time. Log-rank Mantel–Cox analysis: Stim×10 vs. No Stim, $\chi^2 = 5.09$, $P = 0.024$; Stim×10 vs. Stim×3, $\chi^2 = 10.20$, $P = 0.0014$; Stim×3

vs. No Stim, χ^2 = 0.49, P = 0.49. ANCOVA controlling for locomotor activity revealed a significant main effect of stimulation group on feeding latency ($F_{(2, 26)}$ = 6.75, P = 0.0045), whereas total distance during the test ($F_{(1, 26)}$ = 0.72, P = 0.40) and sex ($F_{(1, 26)}$ = 3.36, P = 0.079) were not significant.

[Original (1st revision): Extended Data Fig. 5, legend]

Extended Data Figure 5. REPOPS for 10 days induces anti-depressive behavioral changes in mice.

c, Novelty-suppressed feeding test. Latency to feed per mouse shown as Kaplan–Meier survival curves depicting the probability of not eating over time. Log-rank Mantel–Cox analysis: Stim×10 vs. No Stim, χ^2 = 5.09, P = 0.024; Stim×10 vs. Stim×3, χ^2 = 10.20, P = 0.0014; Stim×3 vs. No Stim, χ^2 = 0.49, P = 0.49. ANCOVA controlling for locomotor activity revealed a significant main effect of stimulation group on feeding latency ($F_{(2, 26)}$ = 6.75, P = 0.0045), whereas total distance during the test ($F_{(1, 25)}$ = 0.72, P = 0.40) and sex ($F_{(1, 25)}$ = 3.36, P = 0.079) were not significant.

[Revised (2nd revision): Extended Data Fig. 5, legend] Revised

Extended Data Figure 5. REPOPS for 10 days induces anti-depressive behavioral changes in mice.

c, Immobility (%) in the forced swim test. Stimulation group: $F_{(2, 26)}$ = 4.28, P = 0.025; Timebin: $F_{(3.61, 93.81)}$ = 143.73, P < 0.001; interaction: $F_{(7.22, 93.81)}$ = 0.74, P = 0.65. Observed powers (1−β) were 0.82, >0.99, and 0.30, respectively.

[Original (1st revision): Main Text, L218]

Depression-related behavior was assessed with the tail suspension test (Fig. 3d). Two weeks after the last stimulation, mice in the Stim×10 group showed significantly less immobility compared to those in the No Stim (P = 0.0094) and Stim×3 groups (P = 0.0198). A similar trend was observed in the forced swim test (Fig. 3e), where Stim×10 mice showed reduced immobility two weeks after the last stimulation compared to the No Stim group (P = 0.0304) and Stim×3 group (P = 0.0770). These results suggest that repeated neuronal activation mimicking ECT reduces depression-like behavior in mice (for other behavioral tests, see also Extended Data Fig. 5 and 6).

[Revised (2nd revision): Main Text, L218] Revised

Depression-related behavior was assessed with the tail suspension test (Fig. 3d). Two weeks after the last stimulation, mice in the Stim×10 group showed significantly less immobility

compared to those in the No Stim (P = 0.0094) and Stim×3 groups (P = 0.0198). A similar trend was observed in the forced swim test (Extended Data Fig. 5c), where Stim×10 mice showed reduced immobility two weeks after the last stimulation compared to the No Stim group (P = 0.0304) and Stim×3 group (P = 0.0770). These results suggest that repeated neuronal activation mimicking ECT reduces depression-like behavior in mice. We further performed the novelty-suppressed feeding test, a well-established assay for antidepressant-like effects that is less dependent on locomotor activity[6] (Fig. 3e). In this paradigm, food-deprived mice are placed in a brightly lit open field with a food pellet in the center, and the latency to begin feeding is recorded. Feeding latency differed significantly among the three groups (log-rank Mantel–Cox test: χ^2 = 11.86, P = 0.0027). Post hoc pairwise comparisons revealed that feeding latency was significantly shorter in the Stim×10 group than in both the No Stim (χ^2 = 5.09, P = 0.024) and Stim×3 groups (χ^2 = 10.20, P = 0.0014), whereas no significant difference was detected between the No Stim and Stim×3 groups (χ^2 = 0.25, P = 0.62; Fig. 3e). To determine whether this effect was attributable to differences in general activity levels, ANCOVA controlling for locomotor activity confirmed that the main effect of stimulation group on feeding latency remained significant ($F_{(2, 18)}$ = 6.93, P = 0.0059). When sex and the interaction term were further entered into the model, there were no significant effects of locomotor activity ($F_{(1, 18)}$ = 0.74, P = 0.40), sex ($F_{(1, 18)}$ = 3.44, P = 0.080), or their interaction ($F_{(2, 18)}$ = 0.74, P = 0.49). These results demonstrate that 10-day, but not 3-day, REPOPS reduces anxiety-related behavior independently of general locomotor activity. We also performed the sucrose preference test. No statistically significant differences were observed among groups in sucrose or water intake or in the percentage of sucrose preference (Extended Data Fig. 5d), suggesting that REPOPS does not alter anhedonia-like behavior. Thus, REPOPS increased locomotor activity and reduced depression-like and anxiety-related behaviors, whereas anhedonia-like behavior remained unchanged. This dissociation may reflect the regional specificity of DG-targeted REPOPS, as the DG and its associated circuits are implicated in exploratory/anxiety-related and approach–avoidance behaviors (Kheirbek et al., *Neuron*, 2013; Fournier, *Neuron*, 2013; Yeates et al., *Hippocampus*, 2020), whereas hedonic tone is primarily supported by mesolimbic reward circuitry (Russo & Nestler, *Nat Rev Neurosci*, 2013).

[Original (1st revision): Supplementary Results 1]

Supplementary Results 1. Behavioral analysis of optogenetically stimulated mice (related to Extended Data Figs. 5 and 6)

To further examine whether REPOPS alleviates depression-related behavior, we performed the novelty-suppressed feeding test, a well-established assay for antidepressant-like effects

that is less dependent on locomotor activity[6]. In this paradigm, food-deprived mice are placed in a brightly lit open field with a food pellet in the center, and the latency to begin feeding is recorded. Feeding latency differed significantly among the three groups (log-rank Mantel–Cox test: $\chi^2 = 11.86$, $P = 0.0027$). Post hoc pairwise comparisons revealed that feeding latency was significantly shorter in the Stim×10 group than in both the No Stim ($\chi^2 = 5.09$, $P = 0.024$) and Stim×3 groups ($\chi^2 = 10.20$, $P = 0.0014$), whereas no significant difference was detected between the No Stim and Stim×3 groups ($\chi^2 = 0.25$, $P = 0.62$; Extended Data Fig. 5c). To determine whether this effect was attributable to differences in general activity levels, ANCOVA controlling for locomotor activity confirmed that the main effect of stimulation group on feeding latency remained significant ($F_{(2, 18)} = 6.93$, $P = 0.0059$). When sex and the interaction term were further entered into the model, there were no significant effects of locomotor activity ($F_{(1, 18)} = 0.74$, $P = 0.40$), sex ($F_{(1, 18)} = 3.44$, $P = 0.080$), or their interaction ($F_{(2, 18)} = 0.74$, $P = 0.49$). These results demonstrate that 10-day, but not 3-day, REPOPS reduces anxiety-related behavior independently of general locomotor activity. We also performed the sucrose preference test. No statistically significant differences were observed among groups in sucrose or water intake or in the percentage of sucrose preference (Extended Data Fig. 5d). These findings suggest that REPOPS does not alter anhedonia-like behavior, although the high baseline sucrose preference in wild-type mice may have limited the sensitivity of this test.

We also assessed the impact of REPOPS on memory function,…

[Revised (2nd revision): Supplementary Results 1] removed

Supplementary Results 1. Behavioral analysis of optogenetically stimulated mice (related to Extended Data Fig. 6)

---removed---

We assessed the impact of REPOPS on memory function,…

Comment 2

The abstract states "…observed ECT-like behavioral effects, including decreased depression-like behavior and increased locomotor activity". Although the authors cite a single study of 15 patients in which psychomotor activity was increased in patients treated with ECT who achieved remission, to frame hyperlocomotion as an accepted therapeutic effect of ECT is an overstatement.

Response:

We appreciate this comment. We agree that increased locomotor activity in our model should not be generalized as an accepted therapeutic outcome of ECT in humans. Accordingly, we revised the Abstract to avoid describing increased locomotor activity as an "ECT-like" behavioral effect. In addition, to place REPOPS-induced hyper-locomotor activity of mice in an appropriate clinical context, we expanded the Discussion and added two additional clinical citations beyond Winkler et al. 2014 (n=15): van Diermen et al. 2019 reported improvements in psychomotor symptoms and objective activity-related measures, in adults with MDD (n=73) undergoing an ECT course; Powell et al. 2020 also demonstrated in depressed inpatients undergoing ECT (n=12) that ECT improved objective indices of psychomotor retardation, including bradykinesia and immobility. Taken together, in the revised manuscript we clarify that the locomotor phenotype observed in REPOPS mice should not be interpreted as a "therapeutic effect" of ECT per se, but rather more conservatively as a phenomenon related to stimulation-induced behavioral activation and/or changes in psychomotor activity measures.

Change:

[Original (1st revision): L179]

These results indicate that repeated neuronal activation causes a significant increase in baseline locomotor activity, which persists for more than one month. Notably, in humans, effective antidepressant treatment is accompanied by increased spontaneous motor activity (Volkers et al., Eur. Neuropsychopharmacol., 2002; Winkler et al., J. Psychiatr. Res., 2014; Spulber et al., Transl. Psychiatry, 2022)[51–53], supporting the relevance of the enhanced activity observed in our model.

[Revised (2nd revision): L213] Revised

These results indicate that repeated neuronal activation causes a significant increase in baseline locomotor activity, which persists for more than one month. Notably, while it has not been an accepted therapeutic effect, ECT in humans is reported to be accompanied by increased spontaneous motor activity (Winkler et al., J. Psychiatr. Res., 2014; van Diermen et al., J. Psychiatr. Res., 2019; Powell et al., J. Psychiatr. Res., 2020)[51–53], supporting the relevance of the enhanced activity observed in our model.

Change:

[Original (1st revision): L37]

Here, we modeled ECT with repeated optogenetic neuronal stimulation in the mouse dentate gyrus, and observed ECT-like behavioral effects, including decreased depression-like behavior and increased locomotor activity.

[Revised (2nd revision): L37] Revised

Here, we modeled ECT with repeated optogenetic neuronal stimulation in the mouse dentate gyrus, and observed ECT-relevant behavioral changes, including decreased depression-like behavior and increased locomotor activity.

Comment 3

Extended Data Figure 17b-

the mice that died soon after behavioral assessments should be excluded from statistical analysis rather marked on the graph as the 3 in the REPOPS group exhibit a marked reduction in locomotor activity and are likely artificially reducing the group average.

Response:

We thank the reviewer for this important comment. We agree that mice that exhibited severe CORT-related debilitation and died shortly after the open field test could bias the interpretation of results. We have performed an analysis excluding these mice (CORT n=2; CORT/Stim×10 n=3), and the CORT/Stim×10 group showed significantly greater distance traveled than the CORT group (P = 0.0166). To make this clear, we revised the legend of Extended Data Fig. 17b and Supplementary Results 3 to explicitly report both analyses (including all mice and excluding the debilitated mice).

Change:

[Original (1st revision): Extended Data Fig. 17, legend]

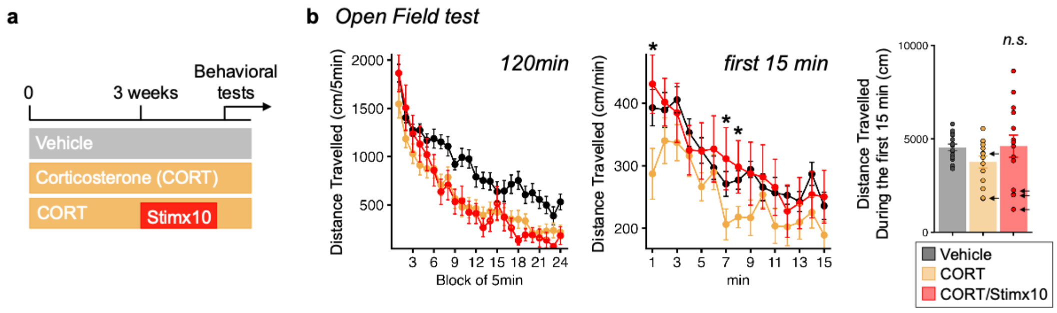

Extended Data Figure 17. REPOPS induces anti-depressive behavioral changes in chronic corticosterone-treated mice.

b, Distance traveled in the open field test during 120 min and in the first 15 min. Two-way repeated-measures ANOVA with Bonferroni correction; $^*P < 0.05$. Vehicle: 16 mice; CORT: 14 mice; CORT/Stim×10: 14 mice. Arrows indicate mice that became debilitated during CORT administration and died after the experiment.

[Revised (2nd revision): Extended Data Fig. 17, legend] revised

Extended Data Figure 17. REPOPS induces anti-depressive behavioral changes in chronic corticosterone-treated mice.

b, Distance traveled in the open field test. Time-course data (120 min) were analyzed by two-way repeated-measures ANOVA with Bonferroni correction; $^*P < 0.05$. Vehicle: 16 mice; CORT: 14 mice; CORT/Stim×10: 14 mice. Total distance traveled in the first 15 min was compared by one-way ANOVA followed by Tukey's HSD test. Mice that became debilitated during CORT administration and died shortly after the open field test are indicated by arrows (CORT n=2, CORT/Stim×10 n=3); results excluding these mice are reported in Supplementary Results 3 (P = 0.0166).

[Original (1st revision): Supplementary Results 3]

Supplementary Results 3. REPOPS reduces depression-like behavior in chronic corticosterone-treated mice (related to Extended Data Fig. 17).

…

In the open field test, mice in the CORT and CORT/Stim×10 groups exhibited markedly reduced locomotor activity compared with controls (Extended Data Fig. 17b). Immediately after exposure to the open field, mice in the CORT/Stim×10 group showed higher locomotor activity than in the CORT group at 1, 7, and 8 min of the test (Extended Data Fig. 17b). There was no significant difference in total distance traveled among the three groups. However, after excluding mice that showed severe CORT-related debilitation and died shortly after the open field test (arrows in Extended Data Fig. 17b), locomotor activity in the CORT/Stim×10 group was significantly greater than in the CORT group ($P = 0.0166$). These results suggest that REPOPS increased locomotor activity under CORT-induced conditions (Extended Data Fig. 17b).

…

[Revised (2nd revision): Supplementary Results 3] revised

Supplementary Results 3. REPOPS reduces depression-like behavior in chronic corticosterone-treated mice (related to Extended Data Fig. 17).

…

In the open field test, mice in the CORT and CORT/Stim×10 groups exhibited markedly reduced locomotor activity compared with controls (Extended Data Fig. 17b). Immediately after exposure to the open field, mice in the CORT/Stim×10 group showed higher locomotor activity than in the CORT group at 1, 7, and 8 min of the test (Extended Data Fig. 17b). There was no significant difference in total distance traveled in the first 15 min among the three groups. However, after excluding mice that showed severe CORT-related debilitation and died shortly after the open field test (arrows in Extended Data Fig. 17b; CORT n=2, CORT/Stim×10 n=3), locomotor activity in the CORT/Stim×10 group was significantly greater than in the CORT group (*P* = 0.0166; one-way ANOVA followed by Tukey's HSD test). These results suggest that REPOPS increased locomotor activity under CORT-induced conditions (Extended Data Fig. 17b).

…

%%%%% end of file %%%%%

Response to Reviewers

Manuscript: NCOMMS-25-35773B

"Repetitive Neuronal Activation Regulates Cellular Maturation State via Nuclear Reprogramming"

Murano et al.

Thank you for the favourable decision and for the helpful editorial guidance accompanying it. We have addressed all editorial requests detailed in the Author Checklist, and our point-by-point responses to those editorial requests are provided in the completed checklist file submitted with this revision.

Below we respond to the remaining reviewer comment.

Reviewer #2 (Remarks to the Author)

Comment:

> *"The authors have done an excellent job in incorporating all feedback and I have no additional comments or concerns."*

Response:

> We sincerely thank Reviewer #2 for the positive evaluation of our revised manuscript and for the constructive feedback provided throughout the review process.
